# Spatiotemporal dynamics of the cardioimmune niche during lesion repair

**DOI:** 10.1038/s44161-025-00739-6

**Published:** 2025-11-03

**Authors:** Andy Shing-Fung Chan, Joachim Greiner, Lisa Marschhäuser, Tomás A. Brennan, Stefanie Perez-Feliz, Ankit Agrawal, Helene Hemmer, Katrin Sinning, Jennifer Wing Lam Cheung, Zafar Iqbal, Alexander Klesen, Tamara Antonela Vico, Julieta Aprea, Ingo Hilgendorf, Thomas Seidel, Martin Vaeth, Eva A. Rog-Zielinska, Peter Kohl, Franziska Schneider-Warme, Dominic Grün

**Affiliations:** 1 Würzburg Institute of Systems Immunology, Julius-Maximilians-Universität Würzburg, Würzburg, Germany; 2 Institute for Experimental Cardiovascular Medicine, University Heart Center Freiburg-Bad Krozingen, University Medical Center and Faculty of Medicine, University of Freiburg, Freiburg, Germany; 3 Institute of Cellular and Molecular Physiology, Friedrich-Alexander-University of Erlangen-Nürnberg, Erlangen, Germany; 4 Department of Congenital Heart Defects and Paediatric Cardiology, University Heart Center Freiburg-Bad Krozingen, University Medical Center and Faculty of Medicine, University of Freiburg, Freiburg, Germany; 5 Department of Cardiology and Angiology, University Heart Center Freiburg-Bad Krozingen, University Medical Center and Faculty of Medicine, University of Freiburg, Freiburg, Germany; 6 DRESDEN-concept Genome Center, c/o CMCB Center for Molecular and Cellular Bioengineering, Technology Platform of the TUD Dresden University of Technology, Dresden, Germany; 7 CIBSS Centre for Integrative Biological Signalling Studies, University of Freiburg, Freiburg, Germany; 8 CAIDAS - Center for Artificial Intelligence and Data Science, Julius-Maximilians-Universität Würzburg, Würzburg, Germany

**Keywords:** Gene expression analysis, Systems analysis, Inflammation, Cardiovascular biology

## Abstract

The heart is one of the least regenerative organs in humans, and ischemic heart disease is the leading cause of death worldwide. Understanding the cellular and molecular processes that occur during cardiac wound healing is an essential prerequisite to reducing health burden and improving cardiac function after myocardial tissue damage. Here, by integrating single-cell RNA sequencing with high-resolution spatial transcriptomics, we reconstruct the spatiotemporal dynamics of the fibrotic niches after cardiac injury in adult mice. We reveal a complex multicellular network that regulates cardiac repair, including fibroblast proliferation silencing by *Trem2*^high^ macrophages to prevent excessive fibrosis. We further discovered a rare population of progenitor-like cardiomyocytes after lesion, promoted by myeloid and lymphoid niche signals. Culturing non-regenerative mouse cardiomyocytes or human heart tissue with these niche factors reactivated progenitor gene expression and cell cycle activity. In summary, this spatiotemporal atlas provides valuable insights into the heterocellular interactions that control cardiac repair.

## Abstract (web summary)

Chan et al. generate a high-resolution spatiotemporal atlas of healing hearts and reveal cellular networks of lesion repair, including macrophage–fibroblast interactions that control late-stage fibrosis and immune niches that induce cardiomyocyte de-differentiation.

## Main

Myocardial infarction (MI) is the leading cause of death globally, accounting for 16% of all deaths^1^. Due to the limited regenerative capacity of adult hearts, post-MI patients frequently suffer from impaired cardiac output. Fewer than 1% of adult human cardiomyocytes (CMs) can proliferate^2^. In mice, neonatal hearts can fully regenerate through CM de-differentiation and proliferation^3–5^. This capacity is lost within about 7 days after birth^4^ and CMs respond to cardiac injury by developing hypertrophy, characterized by an increased cell size, a shift to prolipid metabolism and upregulation of specific genes, including *Nppa, Nppb* and *Xirp2* (refs. ^4,6^). As the adult heart cannot regenerate, formation of a permanent scar must be tightly regulated to avoid impairment of cardiac function.

Fibrotic scar formation involves a complex, time-dependent communication network of different cell types. Following MI, myeloid cells infiltrate the tissue, secrete proinflammatory cytokines such as interleukin (IL)-6, IL-1β and tumor necrosis factor (TNF)^7^, and clear necrotic myocardium in the damaged region^8^. Ly6C^high^ CCR2^high^ monocyte-derived macrophages (mo/MPs) transition from a proinflammatory to a proreparative state and activate conversion of fibroblasts (FBs) into myofibroblasts (myoFBs)^2^. myoFBs migrate into the ischemic zone (IZ) and deposit extracellular matrix (ECM) proteins, forming a fibrotic scar^9^. While scar tissue is essential for wound closure and mechanical stability^10^, excessive fibrosis can lead to impaired electrical conduction, reduced ejection function and heart failure^11^. Finetuning FB activation and ECM deposition is therefore crucial for optimal healing outcomes. Despite some insights into signaling interactions driving cardiac wound healing^2^, morbidity and mortality due to adverse left ventricle (LV) remodeling remain high: 20% of patients develop heart failure within 12 months after MI^12^.

To improve our understanding of the molecular and cellular processes underlying fibrotic scar formation, we here establish a single-cell resolution spatiotemporal atlas of post-lesion mouse hearts by integrating single-cell RNA-seq (scRNA-seq) and high-resolution spatial transcriptomics. We infer changes in cell states and tissue architecture during the major stages of wound healing (early inflammation, scar formation and maturation). The single-cell spatial resolution reveals the niche composition of the lesion, exposing local cell state dependencies and signaling interactions during scar formation. We discovered a dynamic macrophage–fibroblast crosstalk during the late healing stages that may prevent excessive fibrosis. Furthermore, we describe the signaling niche of a rare population of de-differentiating CMs at earlier healing stages, suggesting that a remnant partial regenerative response may persist in adult hearts. All data can be interactively explored and visualized online at https://www.wuesi.medizin.uni-wuerzburg.de/cardiac_spatiotemporal_atlas/.

## Results

### A spatiotemporal atlas of cardiac scar formation at single-cell resolution

**Fig. 1:**
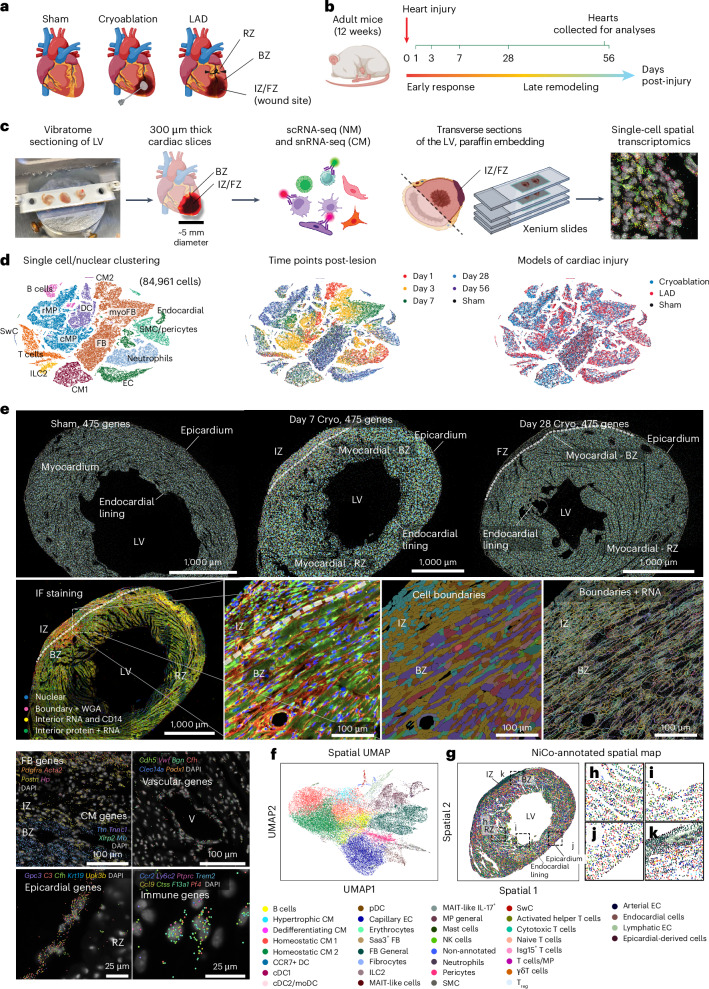
Collection of post-lesion cardiac cells for spatiotemporal single-cell transcriptomic characterization. **a**–**c**, Schematic diagrams of surgery models and responses post-lesion (**a**), experimental design (**b**) and heart sample processing procedure (**c**) (Methods). **d**, *t*-SNEs of cells (NMs) and nuclei (CMs) from all time points and lesion models, composed of 84,961 cells per nuclei. Major cell types; center, time points; right, surgery models (left). **e**, Spatial data from sham day 7 (sham), Cryo day 7 (day 7) and day 28 (day 28) transverse cardiac sections. All detected transcripts from 475 genes (top left). Magnified regions of the day 7 heart, where selected epicardial, vascular, immune, FB and CM genes are shown (right). Cellular counterstaining for cell boundary segmentation (bottom left). Three experiments were performed per condition. **f**,**g**, Spatial expression Uniform Manifold Approximation and Projection for Dimension Reduction (UMAP) (**f**) and spatial location map (**g**) of day 7 heart highlighting cell type annotations (simplified annotation scheme, Methods) by NiCo algorithm. **h**–**k**, zoom-in regions from **g**, representing RZ (**h**), endocardial lining (**i**), epicardium (**j**) and wound region (**k**), respectively. Source data

To explore the spatiotemporal dynamics of cardiac lesion formation at single-cell resolution, we utilized two common mouse models to introduce ventricular lesions: ligation of the left anterior descending coronary artery followed by reperfusion^13^ (LAD) and cryoablation of the LV^14^ (Cryo). LAD leads to an ischemic wound in the LV that resembles acute, reperfused MI in the human heart. During Cryo, tissue is frozen with a cryoprobe to induce severe local lesioning of the LV free wall. Although the Cryo wound does not originate from ischemic damage, its size and location are more consistent across animals compared to the LAD intervention (Fig. 1a).

**Extended Data Fig. 1:**
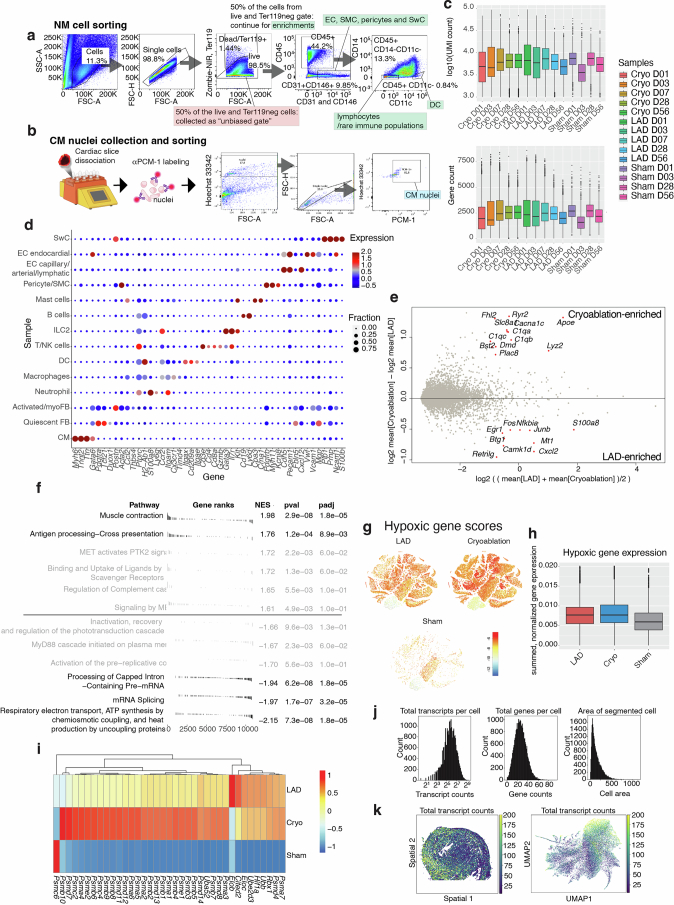
Cell type enrichment, gene expression quantifications and comparison of lesion models. **a**, FACS enrichment and sorting of NM cells. The same gating and collection strategy has been applied across all time points post-lesion to ensure comparable cell type ratios. For each of the collected NM samples for 10x GEM generation, half of the cells were collected from the Zombie-NIR and Ter119-negative gate (highlighted in red), and the other half of the cells were collected from the enrichment gates (highlighted in green). **b**, Preparation and FACS enrichment of CM nuclei. Cardiac slices were dissociated by a gentleMACS dissociator with the protocol “Protein_01.01 M tube”, followed by washing and DAPI and αPCM1 antibody labeling. Labeled nuclei then underwent FACS sorting to enrich for DAPI + PCM1+ nuclei, followed by 10x GEM generation. **c**, Boxplots showing transcript (UMI) and gene counts across cells for each sample (CM + NM). Cryo, cryoablation. From left to right, n = 9179, 10876, 13036, 9353, 8237 (Cryo), 4963, 11327, 4831, 4376, 4817 (LAD), 808, 788, 1000, 1338 (Sham) cells. **d**, Dot plot showing expression z-scores of marker genes for major cell types. Dot size indicates the fraction of cells of the respective cell type expressing the gene. **e,f**, Differentially expressed genes (negative binomial Benjamini–Hochberg adjusted *p* < 0.05, red) (**e**) and differentially enriched pathways (Gene Set Enrichment Analysis^57^ adjusted *p* < 0.05, black) (**f**) in Cryo and LAD samples. **g–i**, Comparison of hypoxia-related genes across LAD and Cryo samples. **g**, *t*-SNEs showing summed expression of hypoxic genes in LAD, Cryo and Sham cells and nuclei. **h**, Quantified normalized sum of hypoxic gene expression across the 3 conditions. n = 30,314 (LAD), 50,681 (Cryo) and 3,966 (Sham) cells. **i**, Heatmap comparing the enrichment of each hypoxic gene across the 3 condition (-1 < score < 1). **j** and **k**, Spatial transcriptomic data transcript quantifications. **j**, Total transcript counts, gene counts (unique transcript counts) and area per segmented cell. **k**, Total transcript count per cell, shown in the spatial map and spatial UMAP, respectively. Box center, median. Box upper and lower bounds, 25 and 75 percentiles. Whisker maxima, 75 percentile + 1.5 interquartile. Whisker minima, 25 percentile - 1.5 interquartile.

Post-lesion-induced LVs were vibratome-cut into tissue slices of 300-µm thickness and ~5-mm radius, centered on the IZ (corresponding to days 1 to 7 of wound healing) or the fibrotic zone (FZ; corresponding to days 28 to 56 of wound healing), and surrounded by border zone (BZ) tissue (Fig. 1a,b). For scRNA-seq, CMs and nonmyocytes (NMs) were isolated from cardiac slices obtained at 1, 3, 7, 28 and 56 days post-surgery (Fig. 1b,c) by enzymatic digestion, followed by antibody labeling and FACS sorting (Extended Data Fig. 1a). We also isolated 4,6-diamidino-2-phenylindole (DAPI)^+^PCM1^+^ nuclei for single-nucleus RNA sequencing (snRNA-seq) of CMs (Extended Data Fig. 1b and Methods). In parallel, spatial transcriptomics using in situ sequencing at single-molecule resolution (10x Xenium) was performed on 5-µm sections of paraffin-embedded LV.

Our single-cell transcriptome atlas contains 84,961 NM cells and CM nuclei from all time points (Extended Data Fig. 1c). Using established marker genes, we annotated major cell populations as CMs, FB/myoFBs, capillary endothelial cells (ECs), endocardial ECs, smooth muscle cells (SMCs)/pericytes, neutrophils, circulatory macrophages (cMPs), resident macrophages (rMPs), B cells, T cells, innate lymphoid cells type 2 (ILC2s) and Schwann cells (SwCs) (Fig. 1d, Extended Data Fig. 1d and Supplementary Tables 1 and 2). Some of these cell types (for example MPs and FB/myoFBs) showed pronounced heterogeneity across different time points. It is known that ejection fraction (EF) sharply declines within the first week after MI and stabilizes thereafter^15,16^. Consistent with this, pathways enriched at days 1–3 (during EF decline) were related to immune activation, RNA metabolism and stress responses, whereas at day 7 (EF stabilization), pathways shifted toward ECM remodeling, energy metabolism and ion channel activity. These associations suggest inflammatory stress during EF loss and partial restoration of CM function during stabilization (Supplementary Fig. 1). Cells obtained from LAD and Cryo were well mixed in the *t*-distributed stochastic neighbor embedding (*t*-SNE) but were separated from sham, suggesting that the reparative processes of the two lesion models are generally similar (Fig. 1d). This was supported by the presence of only 22 differentially expressed genes (DEGs; *P* adjusted <0.05) when comparing LAD and Cryo lesions at the pseudobulk level (Extended Data Fig. 1e). Gene set enrichment analysis revealed condition-enriched pathways related to muscle contraction and antigen processing for Cryo, and respiratory electron transport/ATP synthesis and mRNA splicing for LAD (Extended Data Fig. 1f); however, hypoxia-related genes such as *Hif1a, Ubb* and *Psm*-family genes were upregulated in both models compared to sham (Extended Data Fig. 1g–i). Hence, both lesion types represent physiological MI models with similar molecular responses. Nevertheless, slight transcriptomic differences were detected among early-responding MPs and myoFBs, as discussed below.

We chose an early (day 7) and a late (day 28) time point of the Cryo model along with a day 7 post-sham-surgery control sample for spatial analysis of 475 genes selected based on cell type marker genes derived from the sequencing data (Fig. 1e, Methods and Supplementary Table 3). For each sample, the field of view covered >14,000 cells (Extended Data Fig. 1j,k). To validate the reliability of transcript detection and gene decoding, marker genes of epicardial cells, immune cells, FBs, CMs and vascular cells were visualized (Fig. 1e). Epicardial genes such as *Gpc3* and *Upk3b* were restricted to the epicardial region. Vascular genes such as *Cdh5* and *Vwf* were localized in vessels, whereas immune markers (such as *Ccr2*, *Ly6c2*, *Trem2* and *F13a1*) and FB genes (such as *Pdgfra*, *Acta2* and *Postn*) were enriched in the IZ/FZ. CM genes (such as *Ttn*, *Tnnc1* and *Xirp2*) were restricted to the BZ and remote zone (RZ) (Fig. 1e). We annotated cell types in the spatial data by label transfer from the sequencing data using the NiCo algorithm^17^ (Fig. 1f,g, Supplementary Fig. 2 and Supplementary Tables 4 and 5).

### Dynamic waves of immune cell populations during scar formation

**Fig. 2:**
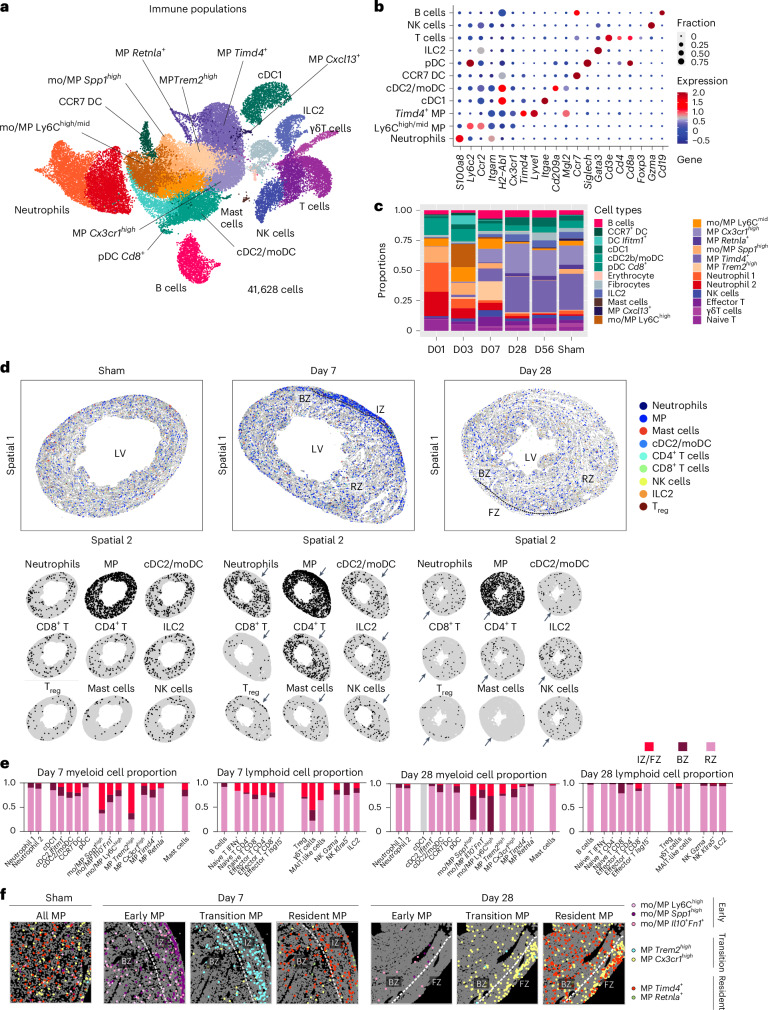
Spatiotemporal cell type dynamics of the immune compartments. **a**, UMAP of scRNA-seq data of immune populations from day 1–56 post-lesion. Color code of cell types is also indicated in **c**. **b**, DEGs for each immune population. Dot size indicates fraction of cells expressing the gene. Dot color indicates normalized expression level. **c**, Immune cell type proportions across days 1–56 and sham. **d**,**e**, Spatial maps of immune cells for sham, day 7 and day 28 (**d**), with quantification of myeloid/lymphoid cell frequencies across IZ/FZ, BZ and RZ for day 7 and day 28 samples under the detailed annotation scheme (**e**). Spatial maps of individual cell types are shown (**d**, bottom). Arrows point to the IZ area. **f**, Spatial distribution of different mo/MP subtypes in the wound regions (or the edge of LV for sham). Dotted lines separate IZ/FZ and BZ.

**Extended Data Fig. 2:**
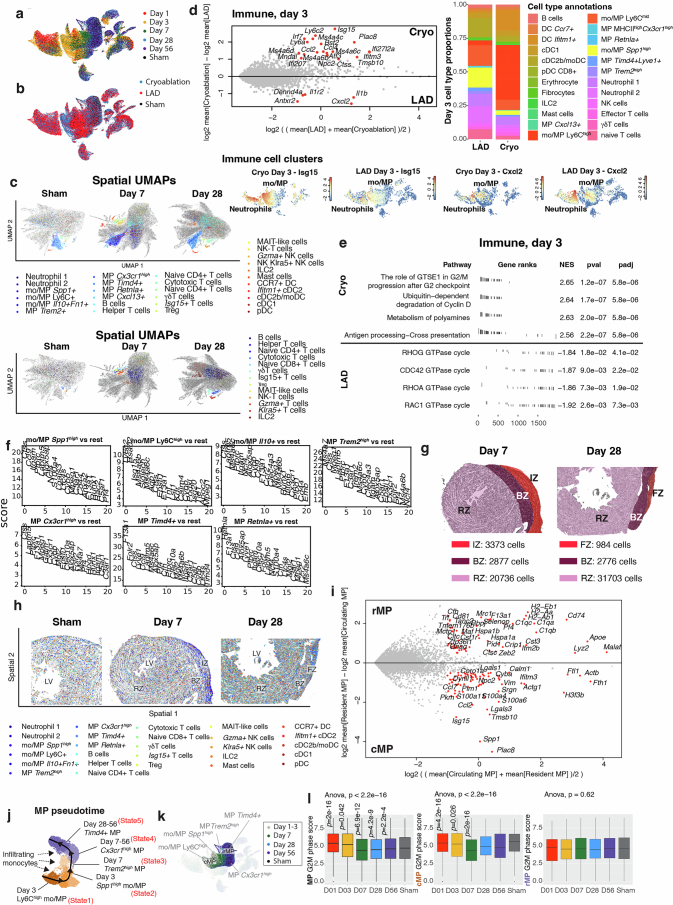
Macrophage scRNA-seq and spatial gene expression profiles. **a and b**, UMAP of immune cell populations highlighting time point post-lesion (**a**) or surgery type (**b**). **c**, Spatial UMAP showing all NiCo-annotated immune cell (sub-)populations (upper panel) or lymphocytes only (lower panel). **d**, Upper left panel, differential gene expression analysis (adjusted *p* < 0.05 and log_2_-foldchange > 1, red) between day 3 Cryo and LAD immune cells. Upper right panel, immune cell type proportions on day 3 LAD and Cryo. Lower panels, gene expression UMAPs of *Isg15* and *Cxcl2* in day 3 Cryo and LAD immune cells, respectively. **e**, Gene set enrichment analysis (GSEA) top enriched pathways in day 3 Cryo and LAD immune cells. Statistics, Gene Set Enrichment Analysis^67^ adjusted *p*. **f**, Top 20 DEGs of NiCo-annotated mo/MP cell types in the spatial data. **g**, Definition of tissue regions for day 7 and day 28 samples, that were used for spatial cell type proportion analysis. **h**, Spatial maps of all immune (sub-)populations in sham, day 7, and day 28 (detailed annotation scheme). **i**, Differential gene expression analysis (negative binomial Benjamini–Hochberg adjusted *p* < 0.05, red) between rMP and cMP. **j** and **k**, Inferred MP cell fate transition (**i**) and changes in enriched cell states across different time points (**k**). **l**, summed expression of G2M cell cycle genes per cell (Methods) in all MP (left), cMP (middle) and rMP (right) across all time points post-lesion, and sham. One-way ANOVA for multiple comparisons was performed. ns, not significant. From left to right, n = 1979, 5502, 4035, 3502, 2926, 748, 1912, 5110, 2261, 156, 120, 105, 54, 353, 1723, 3316, 2791 and 641 cells. Box center, median. Box upper and lower bounds, 25 and 75 percentiles. Whisker maxima, 75 percentile + 1.5 interquartile. Whisker minima, 25 percentile - 1.5 interquartile.

The combination of unbiased sampling and enrichment of rare cell populations yielded a comprehensive spatiotemporal atlas of immune cell type dynamics during scar formation (Fig. 2a–c and Extended Data Fig. 1a; see Methods for cell enrichment protocol). Most myeloid cell types, including neutrophils, MPs, dendritic cells (DCs) and mast cells, were highly enriched on days 1–7; this was followed by expansion of lymphoid populations on day 7, and restoration of sham-like cell type proportions on days 28 and 56 (Fig. 2c and Extended Data Fig. 2a–c). Despite limited global expression differences between Cryo and LAD (Fig. 1g–i), 15 out of 22 DEGs belong to immune (such as *C1qa*, *C1qb*, *Nfkbia* and *S100a8*) and stress (*Fos* and *Junb*) categories, suggesting that model differences mainly arise from the immune compartment. Moreover, minor differences in immune cell type proportions were identified between the two lesion models, predominantly observed among day 3 mo/MPs. A higher proportion of the *Spp1*^*high*^ subtype was detected in LAD, whereas more of the Ly6C^high/mid^ subtype was detected in Cryo (Extended Data Fig. 2b,d). This difference was also reflected in gene expression profiles, where 18 genes were significantly higher during Cryo, mostly composed of cell cycle, metabolic and antigen- presentation genes (Extended Data Fig. 2d,e), whereas higher expression of inflammatory genes such as *IL1b* and *Cxcl2* was detected in immune cells during LAD; however, despite the observed differential expression, all these genes are expressed in both models, among neutrophils and MPs (Extended Data Fig. 2d). Furthermore, these overall limited differences in MP and neutrophil responses between the two modes of injury were resolved at mid–late time points (Supplementary Fig. 3), suggesting a more generic subsequent wound healing process.

NiCo recovered all major immune cell populations in the spatial modality (Fig. 2d and Extended Data Fig. 2c,f). The immune compartment was dominated by MP, localized to the IZ on day 7, whereas the immune cell density was globally reduced on day 28 (Fig. 2d and Extended Data Fig. 2g,h).

Throughout the process of scar formation, dynamic turnover of the circulatory (cMPs) and cardiac resident MPs (rMPs) was observed (Extended Data Fig. 2i). Consistent with previous findings^7,18,19^, the lesion was dominated by *Spp1+* monocyte/macrophages (mo/MPs) and *Trem2*^*high*^ MPs on day 7, whereas *Cx3cr1*^*high*^ MPs became more prevalent on day 28 (Fig. 2e,f). It is reported that Ly6C^high/mid^ mo/MPs can differentiate first into *Spp1*^*high*^, followed by *Trem2*^*high*^*Gdf15*^*high*^ MPs^19^, whereas another study showed that Ly6C^high/mid^ mo/MPs can differentiate into *Cx3cr1*^*high*^ rMPs instead^18^. Consistently, our pseudotime analysis suggested a lineage path that connects all these states in a sequential manner, from Ly6C^high/mid^ and *Spp1*^*high*^ cMP states, to the *Trem2*^*high*^ and *Cx3cr1*^*high*^ rMP state. The day 7-enriched *Trem2*^*high*^ population^19^ may represent a transitory state between cMPs and rMPs (Extended Data Fig. 2j,k). Notably, the cMP population undergoes active proliferation after tissue infiltration (days 1–3), which is suppressed by day 7. In contrast, proliferation of rMPs does not change over time (Extended Data Fig. 2l). The lower cell numbers of the cMPs at mid-to-late stages of lesion repair could be attributed to cell cycle suppression and phenotypic conversion into rMPs, whereas the expansion of the rMP population most likely results from phenotypic conversion of cMPs.

**Extended Data Fig. 3:**
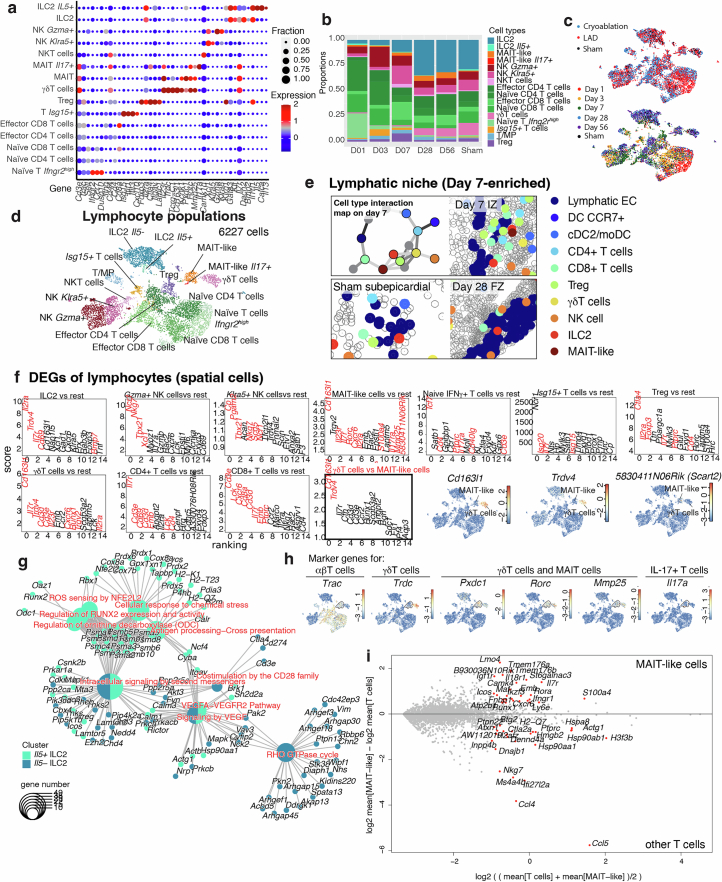
Spatiotemporal cell type dynamics of the lymphocyte populations. **a–d**, scRNA-seq of lymphocyte populations (without B cells) for LAD, Cryo and sham on days 1 to 56 post-lesion. **a**, Dot plot showing marker genes for each population. Dot size represents the fraction of cells of the cell type expressing the gene. Dot color indicates normalized expression level. **b**, Barplot showing cell type proportion dynamics of lymphocyte populations across days 1–56 and sham. **c** and **d**, UMAPs of lymphocyte populations highlighting surgery type and time point post-lesion (**c**), and cell type (**d**). **e**, Spatial maps of local lymphocyte clusters with lymphatic EC, found in sham sub-epicardial region, day 7 IZ and day 28 FZ (detailed annotation scheme). Upper left panel, inferred cell type interaction network focusing on lymphocyte populations. **f**, Top 20 DEGs of NiCo-annotated lymphocyte cell types in the spatial data. Known marker genes for the corresponding cell types are highlighted in red. Lower panels, DEG plot and gene expression UMAPs of lymphocytes showing genes that are used to differentiate between γδT cells and MAIT-like cells in the spatial data. **g**, Gene-concept network (Methods) comparing differential pathways between *Il5+* and *Il5-* ILC2. Pathways are highlighted at the center node of each cluster, while the corresponding genes are shown as connected smaller nodes. Sizes of pathway nodes correspond to the number of contributing genes (from the ReactomePA database, differential gene expression log_2_-foldchange > 0.3). **h**, UMAP showing gene expression of marker genes of αβT, γδT, MAIT-like, and *ll17a*^ +^ T cells. Highlighted by dotted line: MAIT-like cells. **i**, Differential gene expression analysis (negative binomial Benjamini–Hochberg adjusted *p* < 0.05, red) between MAIT-like cells and other T cells.

Our dataset also revealed pronounced time-dependent heterogeneity of lymphocyte populations comprising conventional and unconventional T cells, natural killer (NK) cells and ILC2 (Extended Data Figs. 2c and 3a–f). This includes an early (day 1) wound-responding *Il5*^+^ ILC2 population (Extended Data Fig. 3g), populations of γδT cells and *Il17a*^*+*^ mucosal-associated invariant T (MAIT)-like cells (Extended Data Fig. 3h,i), with poorly studied roles in cardiac healing. Most lymphocyte populations expanded and peaked between day 7 and 28 (Extended Data Fig. 3b,c). Spatially, distinct lymphocyte populations were differentially localized. On day 7, T cells were detected in all tissue regions including the IZ. The majority of the CD4^+^ T cells that entered the IZ acquired a regulatory T (T_reg_) phenotype (a subset of CD4^+^ T cells), in accordance with previous finding^20^ (Fig. 2d). Interspersed clusters of lymphocytes dominated by CD4^+^ and CD8^+^ T cells together with lymphatic EC structures were also found in the IZ (Extended Data Fig. 3e and Supplementary Fig. 4), suggesting recruitment via lymphatic vessels. On day 28, lymphatic vessels remained in the scar but the majority of lymphocytes were cleared from the FZ, while persisting in the BZ and RZ (Fig. 2e and Extended Data Fig. 3e).

### Spatiotemporal dynamics and phenotypic conversion of fibroblasts post lesioning

**Fig. 3:**
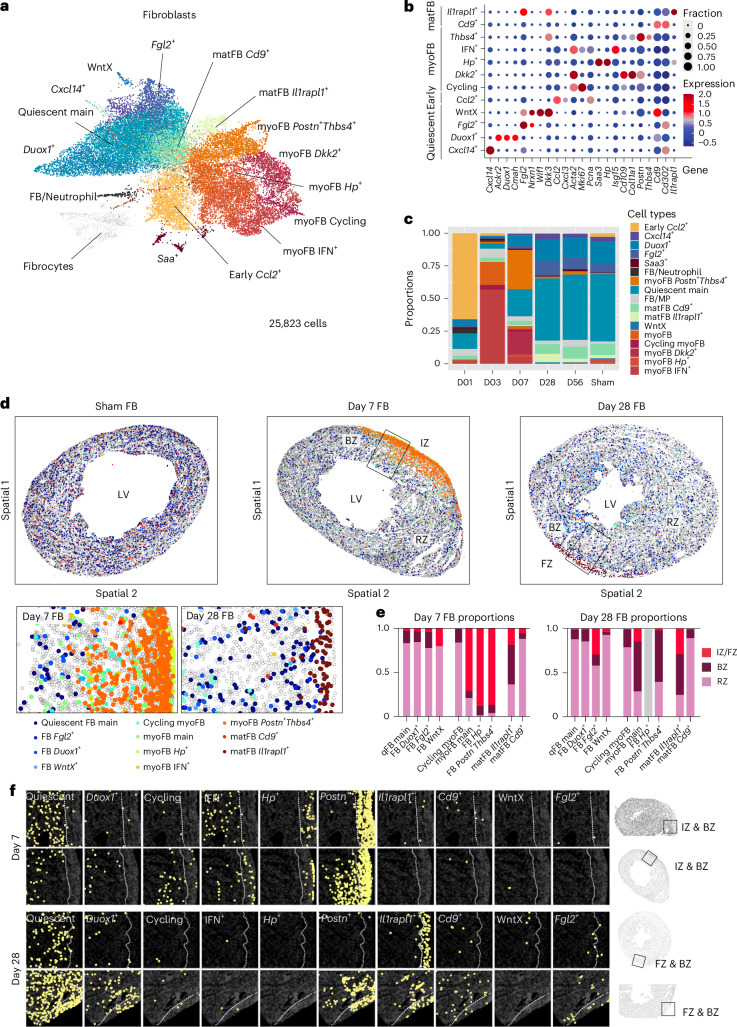
Spatiotemporal cell type dynamics of the fibroblast compartments. **a**, UMAP of scRNA-seq data for FB subtypes from day 1–56 post-lesion. Color code of cell types is indicated in **c**. **b**, DEGs for each FB subtype, grouped into ‘Quiescent’, ‘Early’, ‘myoFB’ and ‘matFB’ states. Dot size indicates fraction of cells expressing the gene. Dot color indicates normalized expression level. **c**, Cell type proportion of FB subtypes across days 1–56 and sham. **d**, Annotated spatial map showing distribution of FB subtypes in day 7 and 28 hearts. **e**, Quantification of FB cell state frequencies across IZ/FZ, BZ and RZ for day 7 and 28 samples. **f**, Spatial maps showing distribution of individual FB subtypes in the wound regions of day 7 and 28 hearts.

FBs are one of the major responders to lesion induction and exhibit a heterogeneous cell state composition (Fig. 3a,b). The main clusters segregated into quiescent FBs, early proinflammatory *Ccl2*^+^ FBs^21^, myofibroblasts (*Acta2*^+^; myoFBs) and matrifibrocytes (*Col8a1*^*+*^*Comp1*^+^*Cd9*^+^*/Il1rapl1*^+^; matFBs)^22^. Quiescent FBs and matFBs were dominant in sham, day 28 and 56 hearts, whereas *Ccl2*^+^ FBs and myoFBs were more abundant on days 1, 3 and 7, respectively (Fig. 3c).

**Extended Data Fig. 4:**
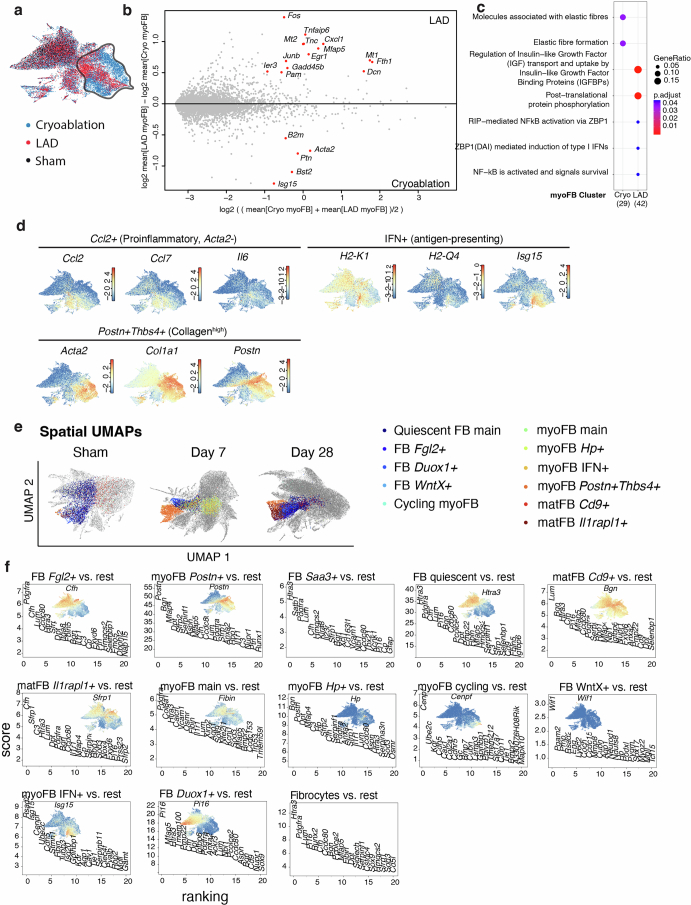
Gene expression profiles of FB activation. **a–c**, Comparison of LAD and Cryo FB populations. **a**, UMAP of FB populations indicating the surgery type. Highlighted cells are myoFB. **b**, Differential gene expression analysis (negative binomial Benjamini–Hochberg adjusted *p* < 0.05, red) between LAD and Cryo. **c**, Pathway enrichment analysis (from the ReactomePA database, differential gene expression log_2_-foldchange > 0.4) comparing between LAD and Cryo. Statistics, Benjamini–Hochberg adjusted hypergeometric test. **d**, FB UMAP showing marker genes for early proinflammatory *Ccl2*^+^ FB, IFN^+^ myoFB and *Postn*^*+*^*Thbs4*^*+*^Collagen^high^ myoFB. **e**, Spatial UMAP showing all NiCo-annotated FB subtypes **(**detailed annotation scheme). **f**, Top 20 DEGs of NiCo-annotated FB subtypes in the spatial data. In each plot, expression of one or more DEG(s) is also plotted in the corresponding scRNA-seq UMAPs showing enrichments of the same DEGs in the corresponding cell subtype across scRNA-seq and spatial data, indicating confident annotation of these cell subtypes. Plots without any scRNA-seq UMAP indicate that the DEGs for this annotation do not match the corresponding cell subtype in the scRNA-seq data, and thus these annotations were not used for downstream analyses.

Minor transcriptomic differences were detected between myoFBs responding to Cryo and LAD, such as upregulation of genes related to AP-1, MAPK kinases and IL-17 signaling pathways in LAD-responding myoFBs (Extended Data Fig. 4a–c); however, no major differences were found between post-LAD and post-Cryo. myoFBs can be further subdivided into interferon (IFN)-responsive antigen-presenting (IFN^+^)^23^, cycling (day 3-enriched), *Hp*^+^, *Dkk2*^+^ and *Postn*^+^*Thbs4*^+^ (refs. ^23,24^) subtypes, with gradual increase in the expression of collagen genes such as *Col1a1*. The IFN^+^ subtype, in particular, showed higher expression of antigen-presenting genes for presenting antigens to CD4^+^ T cells^25^ (Extended Data Fig. 4d and Supplementary Fig. 5). Except for the day 1-enriched *Ccl2*^+^ FBs, all FB states were also observed in the day 7 and 28 spatial data (Extended Data Fig. 4e,f). Quiescent FBs were only found in the BZ and RZ on day 7 post-lesion (Fig. 3d,e). myoFBs were detected both inside and outside of the lesioned region. While IFN^+^ myoFBs were restricted to the BZ, collagen^high^ *Hp*^*+*^ and *Postn*^*+*^*Thbs4*^*+*^ states were highly enriched in the IZ (Fig. 3d,e) suggesting a microenvironment-dependent differentiation of FBs concomitant with their migration toward the IZ. On day 28, both myoFB populations were depleted from the FZ and were replaced by high numbers of matFBs and quiescent FBs, indicating a transition toward a mature and nonproliferative state (Fig. 3d–f).

### Vascular cells and Schwann cells mount an IFN response at early stages post-lesion

**Extended Data Fig. 5:**
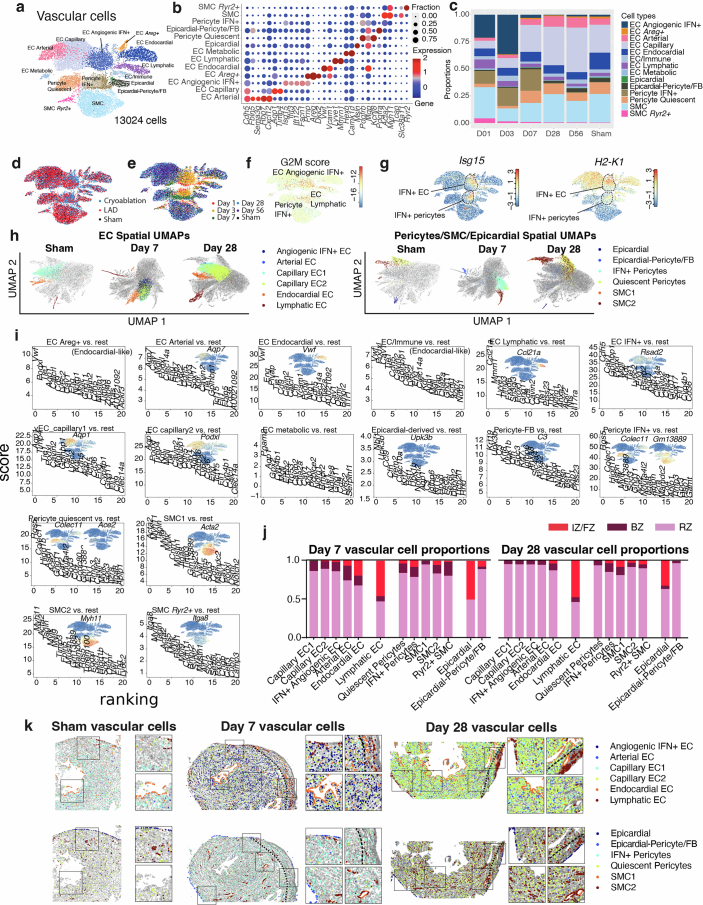
Spatiotemporal cell type dynamics of the vascular populations. **a**, UMAP of scRNA-seq data for vascular populations from day 1 to 56 post-lesion. **b**, DEGs for each vascular subtype. Dot size indicates fraction of cells expressing the gene. Dot color indicates normalized expression level. **c**, Cell type proportion of vascular subtypes across day 1–56 and sham. **d-g**, UMAP of vascular populations highlighting surgery types (**d**), time points post-lesion (**e**), cell cycle (G2M) score (**f**), and expression of IFN pathway gene *Isg15* and antigen-presenting gene *H2-K1*. **h**, Spatial UMAP showing all NiCo-annotated vascular cell types **(**detailed annotation scheme). **i**, Top 20 DEGs of NiCo-annotated vascular cell subtypes in the spatial data. In each plot, expression of one or more DEG(s) is also plotted in the corresponding scRNA-seq UMAPs showing enrichments of the same DEGs in the corresponding cell subtype across scRNA-seq and spatial data, indicating confident annotations of these cell subtypes. Plots without any scRNA-seq UMAP indicate that the DEGs for this annotation do not match the corresponding cell subtype in the scRNA-seq data, and thus these annotations were not used for downstream analyses. **j**, Spatial distribution of vascular subtypes on day 7 and 28. **k**, Spatial maps of EC subtypes (upper panels), epicardial cells, pericytes and SMC (lower panels) for day 7 and 28 hearts.

In addition to the FB response, vascular cells and SwCs also play key roles in post-lesion remodeling. Vascular cells can be generally grouped into ECs, pericytes and SMCs. ECs can be further subclassified into arterial^23^, capillary, endocardial, lymphatic, angiogenic IFN^+^ (proliferative) and *Areg*^+^ states (Extended Data Fig. 5a–g). IFN^+^ ECs/pericytes were abundantly found in the BZ and RZ on day 7, but were less frequent in the IZ. Instead, day-28 ECs mostly comprise quiescent phenotypes in all regions (Extended Data Fig. 5e,h–k). An overall increase of EC density on day 28 indicated global angiogenesis (Extended Data Fig. 5j,k). Lymphatic ECs were already abundant in the IZ on day 7, and exhibited increased density in the FZ on day 28, suggesting prolonged lymphangiogenesis that establishes and maintains lymphatic vessels in the scar region, consistent with previous findings^26^ (Extended Data Fig. 5j,k).

**Extended Data Fig. 6:**
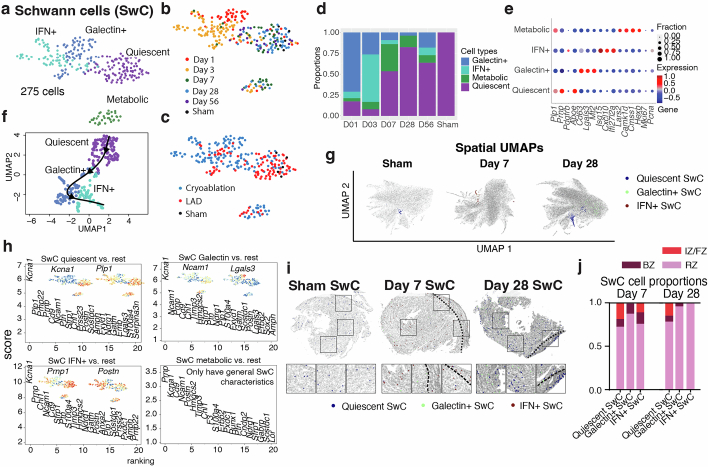
Spatiotemporal cell type dynamics of the SwC populations. **a–c**, UMAP of SwC subtypes highlighting cell states (**a**) surgery types (**b**) or time points post-lesion (**c**). **d**, Barplot showing cell type proportion dynamics of SwC subtypes across days 1–56 and sham. **e**, Dot plot showing DEGs in each SwC subtypes. **f**, Pseudotime analysis of SwC. **g**, Spatial UMAP showing all NiCo-annotated SwC subtypes according to the detailed annotation scheme. **h**, Top 20 DEGs of NiCo-annotated SwC subtypes in the spatial data. In each plot, expression of one or more DEG(s) is also plotted in the corresponding scRNA-seq UMAPs showing enrichments of the same DEGs in the corresponding cell subtype across scRNA-seq and spatial data, indicating confident annotations of these cell subtypes. Alternatively, plots without any scRNA-seq UMAP indicate that the DEGs for this annotation do not match the corresponding cell subtype in the scRNA-seq data, and thus these annotations are not used for downstream analyses. **i**, Annotated spatial maps of SwC subtypes. **j**, Spatial distributions of SwC subtypes in day 7 and 28 hearts.

Despite being a rare cell type in the LV, SwCs (a neural cell type) displayed pronounced temporal and spatial heterogeneity post lesioning (Extended Data Fig. 6a–e). In sham, only a quiescent SwC state was detected (Extended Data Fig. 6d). Upon wounding, SwCs became activated into a Galectin^+^ state (*Lgals3*^+^*Cd63*^+^) on day 1, which upregulated cell cycle, mRNA metabolism and ubiquitin-related pathway genes (Supplementary Fig. 6). This state further transitioned into an IFN^+^ state in the IZ by day 3, similar to the IFN response seen in ECs and pericytes (Extended Data Fig. 6f–j). These states gradually disappeared after day 7, and were replaced by a quiescent state by day 28. The transient activation of SwCs and their spatial distribution across BZ and IZ suggest that, alongside vascular cells, they may contribute to the IFN response and wound healing dynamics during post-lesion remodeling.

### Substantial rewiring of the niche architecture during scar formation

**Fig. 4:**
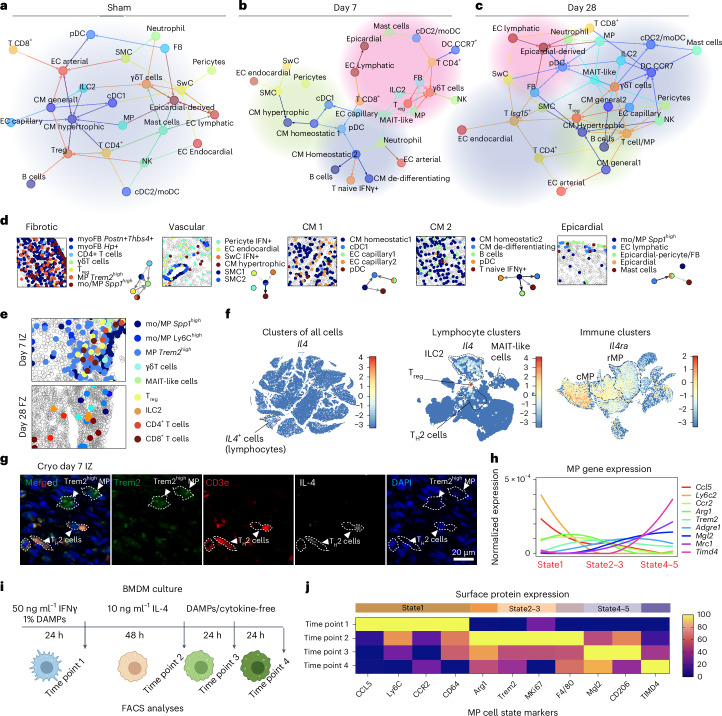
Spatial cell type interactions within immune-vascular niches. **a**–**c**, NiCo spatial interaction network predictions for sham (**a**), day 7 (**b**) and day 28 (**c**) hearts. The thickness of the edges indicates the interaction strength. Arrowheads point from niche cell to central cell. Red shading, IZ/FZ-enriched; green shading, BZ-enriched; blue shading; RZ-enriched. **d**, Representative spatial maps showing day7 spatial niches identified in **b**. **e**, Representative day7 and 28 MP–lymphocyte niches in IZ and FZ. **f**, *Il4* expression in all cells (left) and lymphocyte populations (middle) and *Il4ra* expression among immune populations (right). **g**, Representative IF staining images of *Trem2*^*high*^ MP and IL-4^+^ T_H_2 cells (IL-4^+^CD3e^+^) in the day 7 Cryo IZ region. Three independent experiments were performed. **h**, Expression of cell surface marker genes associated to different MP states. The corresponding surface proteins are quantified in **i**. **i**, Schematic diagram of BMDM culture mimicking the time-dependent microenvironmental changes that infiltrating MPs are exposed to (Methods). **j**, FACS analysis of BMDM culture, showing MP state-associated protein expressions. Source data

The cell state of individual cell types can be affected by heterocellular interactions within their local neighborhoods. We analyzed the spatial data with NiCo^17^ to identify niche interactions. NiCo trains a logistic regression classifier to predict cell type identity from the frequency enrichments of all cell types within the niche across all instances of a cell type. Positive regression coefficients indicate preferential interactions, and NiCo derives a global cell type interaction map from these coefficients for sham, day 7 and day 28 (Fig. 4a–c). Compared to sham and day 28, more distinct spatial interaction domains were observed on day 7. A ‘fibrotic niche’ was dominated by *Postn*^+^*Thbs4*^+^ and *Hp*^+^ myoFB subsets, colocalizing with *Spp1*^*high*^ and *Trem2*^*high*^ MP (as observed previously^27^) and T lymphocytes such as effector CD4^+^ T cells, γδT cells and T_reg_ cells (Fig. 4d). These lymphocytes further connect with NK cells, ILC2, MAIT-like cells and DCs (cDC2/moDCs and CCR7^+^ subtypes), forming clusters of antigen-presenting hubs nearby the IZ lymphatic structures (Fig. 4b, Extended Data Fig. 3e and Supplementary Fig. 4). In the BZ, abundant hypertrophic CM colocalized with vascular cells and SwCs (Fig. 4d). Other CM subtypes formed an interaction hub with capillary ECs, naive T cells, B cells and DCs (including pDCs and cDC1 subtypes) in the RZ (Fig. 4d). At the outer surface of the heart, epicardial cells co-clustered with epicardial derived pericytes/FBs, mast cells, lymphatic vessels and *Spp1*^*high*^ mo/MPs (Fig. 4d). Most of these interaction hubs were largely resolved on day 28.

### Coordinated regulation of mo/MP cell fates by lymphocytes in the IZ over time

**Extended Data Fig. 7:**
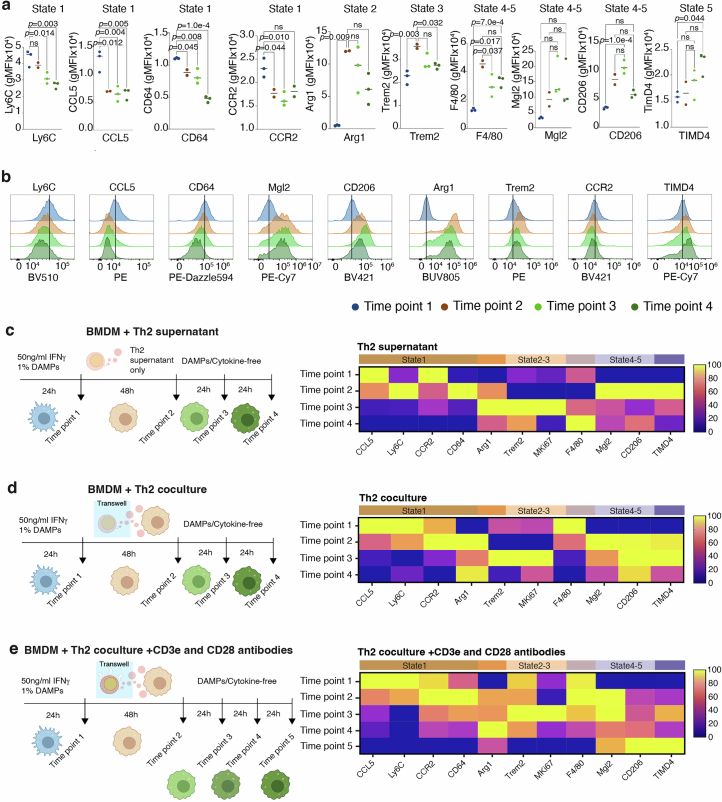
Transient DAMP and IL-4 exposure drives MP state-associated protein expression. **a**, Representative FACS histograms plotting the area-normalized expression of different MP cell state-associated surface proteins across 4 culturing time points. Time point legend colors are indicated in (**b**). Statistics, one-way ANOVA with Tukey’s multiple comparisons test. ns, not significant. Error bars, standard deviation centered at mean. n = 3 biological replicates. **b**, Quantification of cultured MP surface protein expression levels by geometric mean fluorescence intensity (gMFI) from the histogram plots in represented in (**a**). **c-e**, strategies of the BMDM-T_H_2 coculturing system. Left, schematic diagram of culturing and harvesting time points for FACS analysis. Right, FACS analysis readout of normalized MP state marker expression at different time points. Source data

Ly6C^high/mid^, *Spp1*^*high*^ and *Trem2*^*high*^ MPs colocalized with multiple lymphocyte populations on day 7, including ILC2, MAIT-like and effector CD4^+^ T cells (T_H_2) (Fig. 4e). These lymphocytes were the only cells expressing *Il4*, a cytokine known to induce an anti-inflammatory MP polarization^28^ (Fig. 4f). Such high proximity between IL-4^+^ T_H_2 cells and *Trem2*^*high*^ MPs in the day 7 IZ was confirmed by IF staining (Fig. 4g). On day 28, the majority of these lymphocytes were cleared, whereas MPs acquired a resident *Timd4*^+^/*Cx3cr1*^*high*^ phenotype (Figs. 2f and 4e). To determine whether such transient MP–lymphocyte interactions via IL-4 signaling drive the inferred sequential MP cell state changes (Extended Data Fig. 2j), we modeled IZ/FZ MP–lymphocyte niche conditions over time. Bone-marrow-derived macrophages (BMDMs)^19^ were cultured with cardiac damage-associated molecular patterns (DAMPs), necrotic cells and IFNγ, for 24 h to mimic the early post-lesion microenvironment (time point 1). To mimic the day 3–7 MP–IL-4^+^ lymphocyte interactions, the BMDMs were then exposed to IL-4 for 48 h (time point 2), followed by cytokine-free medium for 48 h (time points 3 and 4), mimicking the clearance of lymphocytes in day 28 FZ. At each time point, BMDMs were collected for FACS of surface markers identified from the pseudotime-gene expression profiles (Methods and Fig. 3h,i). DAMP-treated BMDMs upregulated surface markers of the Ly6C^high/mid^/proinflammatory phenotype (state 1). Subsequent exposure to IL-4 was associated with a reduction in Ly6C^high/mid^ markers and increased proportion of *Spp1*^*high*^, *Trem2*^*high*^ MPs expressing the proliferative marker Mki67 (states 2 and 3). After IL-4 removal, cells had decreased *Spp1*^*high*^/*Trem2*^*high*^ MP markers and low Mki67, but upregulated rMP markers (states 4 and 5) (Fig. 4j and Extended Data Fig. 7a,b). To determine whether this IL-4-driven effect on mo/MPs is indeed mediated by T_H_2 cells, the culturing system was modified. Instead of direct exposure to IL-4, DAMP-treated BMDMs were exposed to either the supernatant collected from a separate T_H_2 cell culture, co-cultured (separated by Transwells) with T_H_2 cells or co-cultured with hyperactivated T_H_2 cells (by CD3e and CD28 antibody stimulation) (Extended Data Fig. 7c–e). In all cases, the phenotypic transition of BMDMs resembled the trend observed in IL-4 culture, with an earlier upregulation of resident marker genes, potentially induced by other cytokines such as IL-10 (Extended Data Fig. 7c–e). In summary, our data support a model where transient T_H_2 cell IL-4 signaling induces a transition of proinflammatory mo/MPs into proliferative *Trem2*^*high*^ states, followed by progression into slow-cycling resident phenotypes.

### Mutual control of proliferation by molecular crosstalk of macrophages and fibroblasts in the lesion

**Fig. 5:**
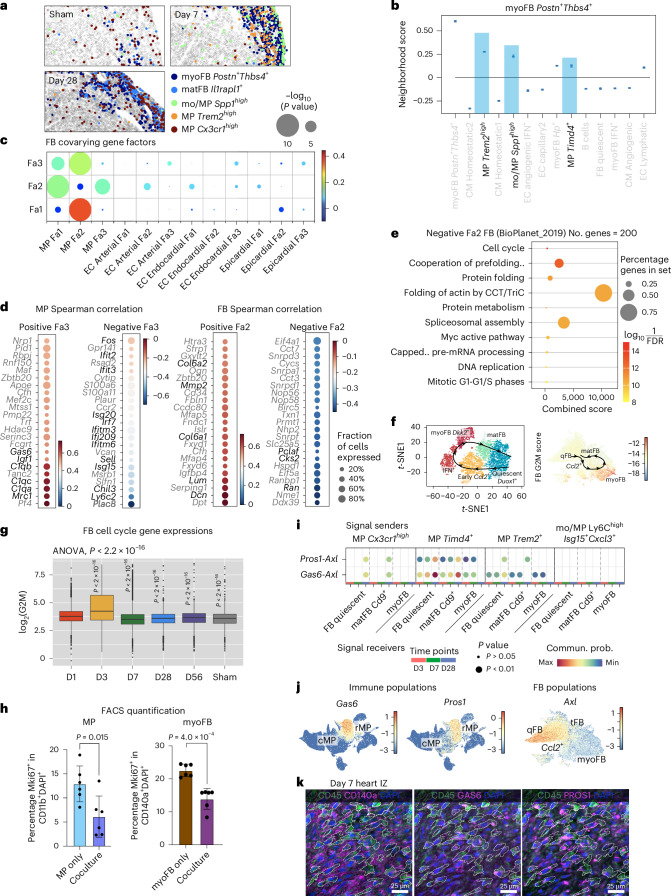
Spatial neighborhoods of the fibrotic niche. **a**, Spatial maps showing MP and FB subtypes in the wound (LV edge in sham). **b**, Day 7 neighborhoods (NiCo interaction scores) surrounding *Postn*^*+*^*Thbs4*^*+*^ myoFBs, with MPs highlighted in blue. **c**, Spatial latent factor (Fa) covariations of MP neighborhood in day 7 heart. Circle size scales linearly with −log_10_(*P* value), and circle color indicates ridge regression coefficients. The multivariate regression *P* value was derived from two-tailed *t*-statistics. **d**, Genes positively and negatively correlated with MP Fa3 (left) and FB Fa2 (right). Highlighted genes for MP Fa3, secreted ligands (positive) and early proinflammatory/IFN pathway (negative). Highlighted genes for FB Fa2, ECM genes (positive) and cell cycle genes (negative). **e**, Pathways enriched in the top 200 genes negatively correlated with FB Fa2 (BioPlanet 2019 database). **f**, Inferred cell fate transition of FB states (top), together with UMAP showing cell cycle G2M score of these states (bottom). **g**, Boxplot comparing log_2_-transformed aggregated G2M gene expression of FB over time (Methods). Statistics used was one-way analysis of variance (ANOVA), *P* < 2.2 × 10^−16^, *n* = 2,759, 5,780, 5,144, 3,078, 4,165 and 1,664 cells. Box center, median. Box upper and lower bounds, 25 and 75 percentiles. Whisker maxima, 75 percentile + 1.5 interquartile. Whisker minima, 25 percentile − 1.5 interquartile. **h**, FACS analysis of the co-cultured MP (CD11b^*+*^) and cardiac myoFBs (CD140a^*+*^) (Methods). Quantification of the proliferation rates in these two cell types was performed and compared between individual culture and co-culture settings. Statistics used were unpaired two-tailed *t*-tests. NS, not significant. Error bars show s.d. centered at mean. *n* = 6 biological replicates. **i**, CellChat ligand–receptor interaction prediction between MP (senders) and FB subtypes (receivers) across days 3–28. Statistics used were permutation tests. **j**, Immune cell UMAP showing *Gas6* and *Pros1* expression, and FB UMAP showing *Axl* expression. **k**, Representative images of multiplexed IF staining of day 7 post-LAD IZ. Dotted lines highlight CD45^*+*^GAS6^*+*^PROS1^*+*^ immune cells contacting myoFBs (CD140a^*+*^). *n* = 3 experiments. Source data

**Extended Data Fig. 8:**
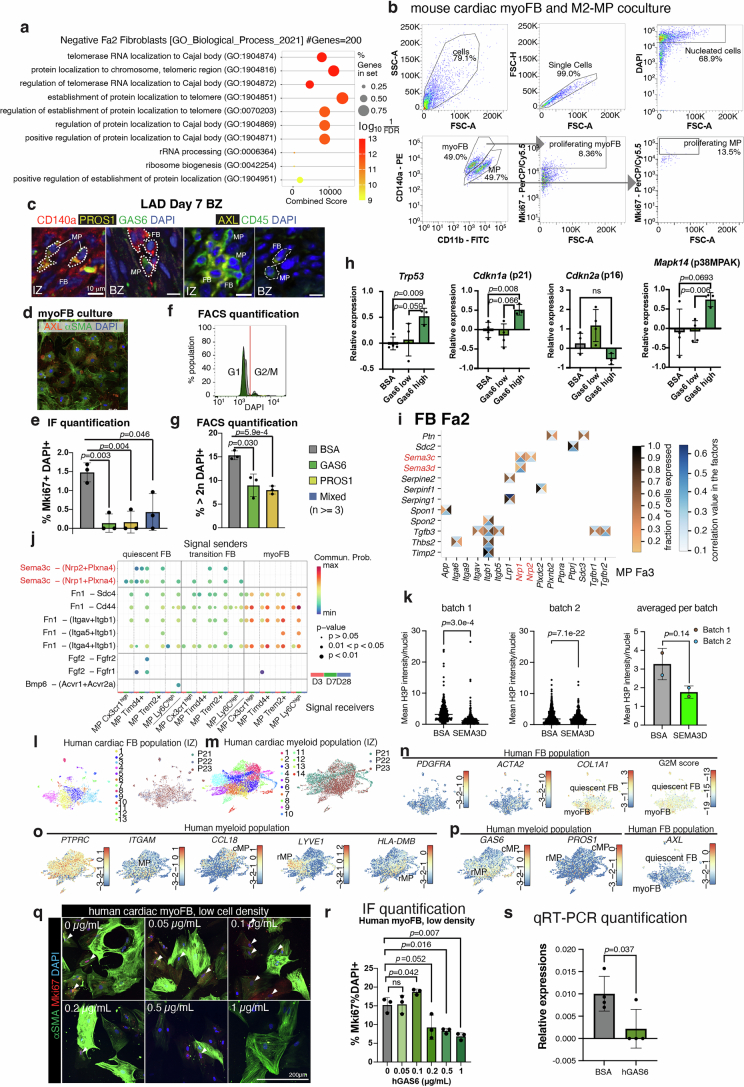
MP-FB interactions in mouse and human hearts post-lesion. **a**, Pathways enriched in the top 200 genes negatively correlated to FB Fa2 (GO Biological Process 2021 database). **b**, Schematic diagram of the FACS analysis gating strategy for the M2 MP and myoFB co-culture experiment (Methods). **c**, IF staining of day 7 post-LAD IZ and BZ of cardiac slices. Dotted lines in left panels highlight MP (CD140a^−^ PROS1^+^ GAS6^+ ^) and FB (CD140a^+^ ). Dotted lines in the right panels highlight cells resembling MP (CD45^+^ AXL^−^) and FB (CD45- AXL + ). n = 2 experiments. **d**, IF staining of AXL in myoFB (αSMA^+^ ) cell culture. n = 2 experiments. **e-h**, Mouse cardiac myoFB culture experiments. Cardiac myoFB exposed to recombinant mouse GAS6, PROS1, GAS6 + PROS1 (mixed), or BSA were harvested for IF Mki67^+^ nuclei quantification (**e**), FACS quantification of the percentage of nuclei with > 2n DNA content, indicated by G1 and G2M peaks of DAPI intensity (**f**,**g**), and qRT–PCR quantification of senescence and cell cycle arrest marker genes (**h**). For (**e**), n = 3 biological replicates. For (**h**) n = 4 biological replicates. **i**, Predicted ligand–receptor interactions, where the ligand and receptor correlate to FB Fa2 and MP Fa3, respectively. Boxes depict correlation with factors (blue) and fraction of cells expressing the gene (brown) **j**, CellChat ligand–receptor interaction prediction from days 3–28. Signal senders are FB subtypes, signal receivers are MP subtypes. Statistics, permutation test. In (**i**) and (**j**), Sema3-Nrp interactions are highlighted in red. **k**, Mouse BMDM culturing. BMDM were exposed to BSA/SEMA3D for 72 hours, then harvested for H3P intensity quantification. Mean H3P intensity per area of nucleus is shown for each experimental batch, where each data point represents a single nucleus (left and mid panels), and the overall average intensity in each group is shown in the right panel. Left panel, BSA n = 516 cells. SEMA3D n = 378 cells. Middle panel, BSA n = 271 cells, SEMA3D n = 401 cells. Right panel, n = 2 experiments. **l-p**, Reanalysis of published snRNA-seq data from 3 MI patients (P21, 22 and 23), isolated from IZ ^6^. **l** and **m**, Human cardiac FB (**l**) and myeloid (**m**) populations. UMAPs highlighting clusters (left panels) and patient identities (right panels). **n-p**, UMAP plots showing expression of FB marker genes and G2M score (**n**), MP marker genes (**o**), and expression of *GAS6*, *PROS1* and *AXL* in these two populations (**p**). Quiescent FB were defined by *PDGFRA+ ACTA2*^*low*^ *COL1A1* ^*low*^ and lower G2M score. myoFB were defined by *PDGFRA*^*+*^ *ACTA2*^*high*^ *COL1A1*^*high*^ and higher G2M score. cMP were defined by *PTPRC*^+^ *ITGAM*^+^ *CCL18*^+^ *HLA-DMB*^*low*^ *LYVE1*^*−*^. rMP were defined by *PTPRC*^+^ *ITGAM*^+^ *CCL18*^*−*^*HLA-DMB*^*high*^*/LYVE1*^+^. **q-s**, Purified human primary cardiac FB culture exposed to recombinant hGAS6 protein. **q**, Representative images of myoFB cultures, stained with αSMA and Mki67 antibodies. **r**, Percentages of proliferating cells (Mki67^+^ DAPI^+^ ) under exposure to different hGAS6 concentrations, determined by IF staining quantification. n = 3 biological replicates. **s**, qRT-PCR quantifications of *MKI67* in myoFB culture. n = 4 biological replicates. Statistics for (**e,g,h,k,r,s**), unpaired two-tailed t-test for two experimental groups. ns, not significant. Error bars, standard deviation. Source data

FB and immune cells are key determinants of cardiac wound healing. These two cell types intensively communicate during the early phase post-injury (day 1–7), via TNF, PDGF, IL-1β and IFNγ signaling^21,25,29–31^, leading to increased energy consumption, hypoxic response and the induction of several inter-dependent FB cell states, which have been confirmed in our spatiotemporal data, pseudotime analysis and in vitro culture (Supplementary Fig. 7). On day 7, *Spp1*^*high*^ mo/MPs, *Trem2*^*high*^ MPs and *Postn*^*+*^*Thbs4*^*+*^ myoFBs are densely enriched in the IZ. The (myo)FBs remain colocalized with *Cx3cr1*^*high*^ MPs until day 28 but acquired an *Il1rapl1*^*+*^ matFB state (Fig. 5a,b). To gain more insights into the effects of cell–cell communication on the cellular states within local neighborhoods, we applied NiCo covariation analysis, which quantifies covariation of cell type-specific latent gene programs, termed factors (Fa), capturing intracell type variability. Covariation of different factors in colocalized cell types indicates the downstream effect of cell–cell communication on the activity of the corresponding gene programs. Inspecting the factor-correlating genes reveals ligands, receptors or pathways that are positively or negatively associated with a factor. In the *Trem2*^*high*^ MP–*Postn*^+^*Thbs4*^*+*^ myoFB neighborhood, NiCo covariation analysis showed that FB and MP latent factors (Fa3 and Fa2, respectively) covary significantly (Fig. 5c). Among the top correlating genes for MP Fa3, we identified the secretory ligand *Gas6* (ref. ^32^), along with complement factors, the mature MP marker *Mrc1*, and growth factor *Igf1* (Fig. 5d). Genes anti-correlating with this factor are associated with stress response (*Fos*), IFN pathways (*Ifitm3* and *Isg15*) and comprise the proinflammatory marker *Ly6c2* (Fig. 5d). The covarying FB Fa2 positively correlates with ECM-remodeling genes such as *Mmp2*, *Lum* and *Dcn*, and anti-correlates with gene sets for cell cycling (Fig. 5d,e) and localization of telomerase RNA to Cajal bodies (Extended Data Fig. 8a), suggesting a reduction in both cell cycle and telomerase activities. Reassuringly, the same FB–MP covariation pattern was consistently detected in all spatial sections (Supplementary Fig. 8–10). Moreover, our scRNA-seq data exhibit high expression of cell cycle genes in FBs on day 3 and downregulation from day 7 onwards (Fig. 5f,g). Co-culture of cardiac myoFB and BMDM-derived M2 MPs (*Trem2*^*high*^ MPs belong to an M2 state derived from the bone marrow mo/MPs) validated mutual suppression of cell cycle activity (Fig. 5h and Extended Data Fig. 8b). Next, we sought to identify the ligand–receptor interactions controlling myoFB cell cycle reduction. Consistent with our spatial data, which revealed GAS6 as a potential ligand, our scRNA-seq ligand–receptor analysis by CellChat predicted *Gas6*–*Axl* and *Pros1*–*Axl* interactions between *Trem2*^*high*^, *Cx3cr1*^*high*^/*Timd4*^*+*^ MPs and myoFB/matFBs, preferentially on day 7 and 28 (Fig. 5i,j). Taken together, these data suggest that the interaction between *Trem2*^high^*/Cx3cr1*^*high*^ MPs and FBs entails silencing of MP inflammation and proliferation, as well as suppression of FB proliferation.

**Fig. 6:**
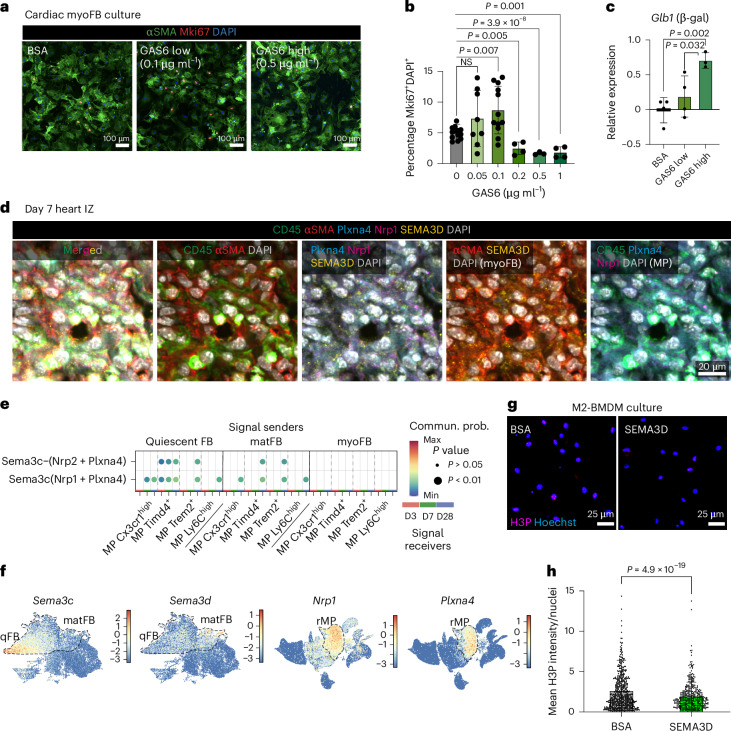
Mutual silencing of MP and myoFB at late stage of fibrosis. **a**–**c**, Mouse cardiac myoFB culturing experiment (Methods). Cultured myoFBs were collected for IF staining with αSMA and Mki67 (**a**), quantification of % Mki67^+^ nuclei (DAPI^+^) in αSMA^+^ cells (myoFB) (**b**), as well as qRT–PCR quantification of senescence marker gene *Glb1* (β-gal) (**c**). *n* = 13, 7, 12, 4, 3 and 4 biological replicates (**b**); *n* = 4 biological replicates (**c**). **d**, Representative images of multiplexed IF staining of day 7 post-Cryo IZ. myoFB are marked by αSMA^+^SEMA3D^+^, MP are marked by CD45^+^Nrp1^+^Plxna4^+^. *n* = 3 experiments. **e**, CellChat prediction for FB (senders) and MP subtypes (receivers), across days 3–28. Statistics used were permutation tests. **f**, UMAPs of FB (left) and immune (right) clusters showing SEMA3 ligand and receptor gene expression, respectively. **g**,**h**, Mouse BMDM culturing. BMDMs were exposed to BSA/SEMA3D for 72 h, then collected for H3P IF (**g**) with intensity quantification (**h**). BSA, *n* = 787 cells; SEMA3D, *n* = 779 cells. Statistics used for **b**,**c**,**h** were unpaired two-tailed *t*-tests. NS, not significant. Error bars show s.d. centered at mean. Source data

We validated ligand–receptor colocalization on the protein level by multiplexed immunofluorescence (IF) in day 7 LAD hearts. Consistent with our spatial data, large numbers of myoFBs (CD140a^*+*^) and MPs (CD45^*+*^GAS6^*+*^PROS1^*+*^) were closely packed in the IZ (Fig. 5k), but not in the BZ and RZ (Extended Data Fig. 8c). IF of TGFβ-activated myoFB (αSMA^*+*^), from FBs isolated from 6-week-old mice, showed protein expression of the GAS6/PROS1 receptor AXL (Extended Data Fig. 8d). To functionally validate this interaction, we performed in vitro culture of myoFBs with recombinant mouse GAS6 ligand and assessed proliferation by Mki67^*+*^ nuclei staining (Fig. 6a,b). Exposure of myoFB to low concentrations of GAS6 led to a slight increase of proliferation, whereas the opposite effect was observed when GAS6 exceeded 0.1 µg ml^−1^ (Fig. 6a,b), indicating a concentration-dependent regulation of myoFB proliferation. A similar effect was observed for high concentration of PROS1 (Extended Data Fig. 8e–g). As our spatial NiCo analysis suggested a reduction of telomerase activity genes alongside with cell cycle genes as a result of the day 7 MP–myoFB interaction, we assessed expression of senescence and cell cycle arrest markers such as *Glb1* (β-galactosidase), *Trp53* (p53) and *Cdkn1a* (p21) in the in vitro myoFB culture. Consistently, exposure to GAS6 at high concentrations induced most of these genes (Fig. 6c and Extended Data Fig. 8h). Hence, abundant GAS6 can trigger both myoFB cell cycle arrest and cellular senescence.

We next aimed to identify myoFB-expressing ligands mediating suppression of proliferation of neighboring *Trem2*^*high*^ MPs, as suggested by our MP–myoFB co-culture data (Fig. 5h), and the anti-correlation of MP Fa3 with activation and proinflammatory phenotypes (Fig. 5d). Expression of the Semaphorin family ligand gene *Sema3d* correlates to FB Fa2 (enriched in qFB and matFB), whereas its receptor genes *Nrp1* and *Plxna4* (ref. ^33^) were upregulated in rMPs; this interaction was also predicted by CellChat (Fig. 6d–f and Extended Data Fig. 8i,j). By performing IF analysis of the day 7 hearts, we confirmed the presence of local clusters of myoFB and MP expressing SEMA3D and Nrp1/Plxna4, respectively in the IZ, supporting the spatial cell–cell interaction data (Fig. 5d). In vitro culture of BMDMs with recombinant mouse SEMA3D ligand validated its proliferation-suppressive function, as demonstrated by a lower mean nuclear phospho-histone 3 (H3P) intensity (Fig. 6g,h and Extended Data Fig. 8k).

Finally, we asked whether this mutual myoFB–MP suppression mechanism is conserved in human hearts. We reanalyzed a recent human MI snRNA-seq dataset^6^ and focused on the IZ cells, which comprise a large population of myoFBs (*PDGFRA*^*+*^*ACTA2*^*+*^*COL1A1*^*high*^) with high cell cycle gene expression (Extended Data Fig. 8l,m). The myeloid population, which mostly consists of *PTPRC*^*+*^*ITGAM*^*+*^ MPs, contains both infiltrating *CCL18*^*+*^ and resident *LYVE1*^*+*^ phenotypes. Similar to our observations in mice, *GAS6* expression is enriched in resident versus circulatory MPs, whereas *AXL* expression is expressed in all FBs (Extended Data Fig. 8o–p). Exposure of primary human cardiac TGFβ-activated myoFBs to different concentrations of recombinant human GAS6 ligand (hGAS6) resulted in a similar trend of proliferation changes as seen in mouse cardiac myoFBs, determined by IF Mki67^*+*^ nuclear quantification and *Mki67* expression quantitative PCR with reverse transcription (qRT–PCR) (Extended Data Fig. 8q–s). Taken together, our findings suggest a conserved regulation of cardiac FB proliferation by GAS6.

### Synergistic induction of cardiomyocyte de-differentiation by their niche

**Fig. 7:**
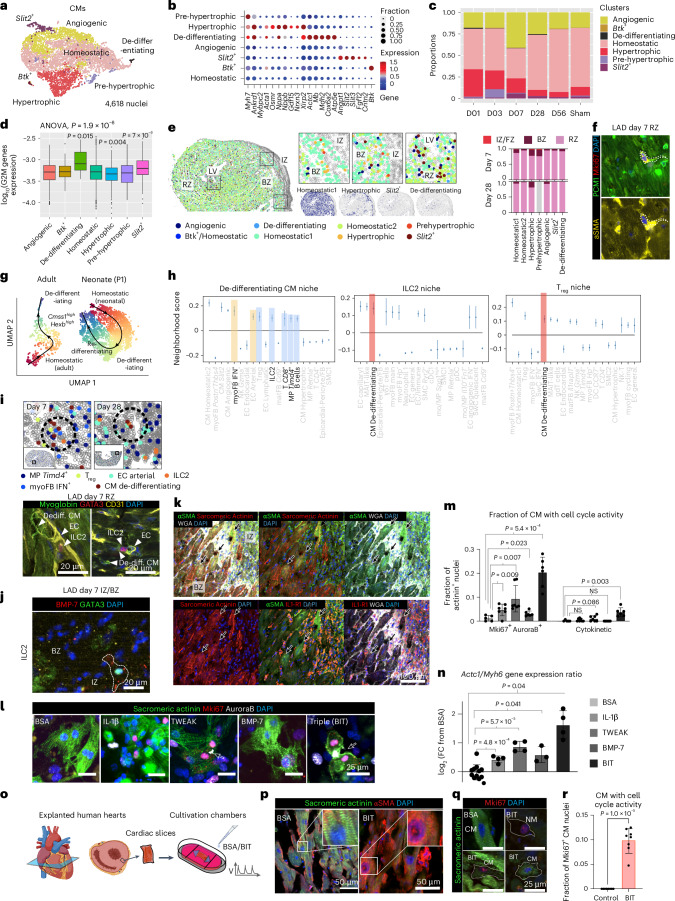
Spatiotemporal interactions of the myocardial niches. **a**, UMAP of scRNA-seq data for CM subtypes from day 1–56 post-lesion. Post-lesion enriched subtypes are highlighted in bold. **b**, DEGs for each subtype. Dot size indicates fraction of cells expressing the gene. Dot color indicates normalized expression level. **c**, CM subtype proportion across days 1–56 and sham. **d**, Quantification of aggregated G2M gene expression across cells (Methods) on logarithmic scale. Statistics used were one-way ANOVAs. Box center, median. Box upper and lower bounds, 25 and 75 percentiles. Whisker maxima, 75 percentile + 1.5 interquartile. Whisker minima, 25 percentile − 1.5 interquartile. *n* = 1,170, 14, 16, 2,260, 452, 87 and 139 nuclei. **e**, Annotated spatial maps of CM subtypes on day 7 with detailed annotation scheme, and quantifications across days 7 and 28. **f**, Representative IF staining images of LAD day 7 heart RZ sections with PCM1, Mki67 and αSMA. Mki67 labels proliferating nuclei, and αSMA labels de-differentiating CMs. *n* = 2 experiments. **g**, Comparison of pseudotime trajectory from homeostatic to the de-differentiated state in adult versus neonatal CMs post-lesion^4^. **h**, Spatial neighborhoods (NiCo interaction scores) of de-differentiating CMs, ILC2 and T_reg_ cells on day 7. Neighborhood scores, NiCo regression coefficients. Error bars, s.e. of the coefficient estimates. **i**, De-differentiating CM niche visualization on days 7 and 28, visualized by the spatial transcriptomic data (top) and IF staining (bottom, LAD day 7 RZ), respectively. For the IF staining, de-differentiating CMs (Myoglobin^*+*^), ECs (CD31^*+*^) and ILC2 (Gata3^*+*^) were detected. *n* = 2 experiments. **j**,**k**, IF of day 7 LAD in IZ-BZ for ILC2 (KIT^*+*^GATA3^*+*^BMP-7^*+*^) (**j**) and de-differentiating CMs (sarcomeric actinin^*+*^ aSMA^*+*^) (**k**). *n* = 2 experiments. WGA labels cell membranes. Arrows point to IL-1R1^*+*^ de-differentiating CMs. **l**, Primary P7-CM culturing (Methods). Cells were exposed to ligands for 48 h. Representative IF images of ligand-exposed CMs. CMs (sacromeric actinin^*+*^) with Mki67^*+*^AuroraB^*+*^DAPI^*+*^ nuclei exhibit cell cycle activity. White arrows point to cytokinetic morphology indicating CM cell division. **m**, Image quantifications of CMs with cell cycle activity (left) and undergoing cytokinesis (right) for data in **l**. **n**, qRT–PCR of *Actc1* (progenitor marker) and *Myh6* (mature marker) of cultured CMs. *Actc1*-to-*Myh6* expression ratios (as a progenitor state score) are shown. From left to right, *n* = 5, 7, 7, 6 and 6 biological replicates. **o**, Schematic diagram of human cardiac slice culture (Methods). **p**, Representative images showing loss of sacromeric structures in the BIT-exposed CMs for human cardiac slice culture. *n* = 2 experiments. **q**, Representative images showing Mki67^*+*^ CMs and NMs in BIT-treated slices, but not in BSA-treated slices. White dotted lines highlight cell boundaries. *n* = 2 experiments. **r**, Quantification of Mki67^*+*^DAPI^*+*^ nuclei in CMs (sarcomeric actinin^*+*^ cells). *n* = 8 biological replicates. Statistics used were unpaired two-tailed *t*-tests for two experimental groups (**m**,**n**,**r**). NS, not significant. Error bars show s.d. centered at mean. Source data

**Extended Data Fig. 9:**
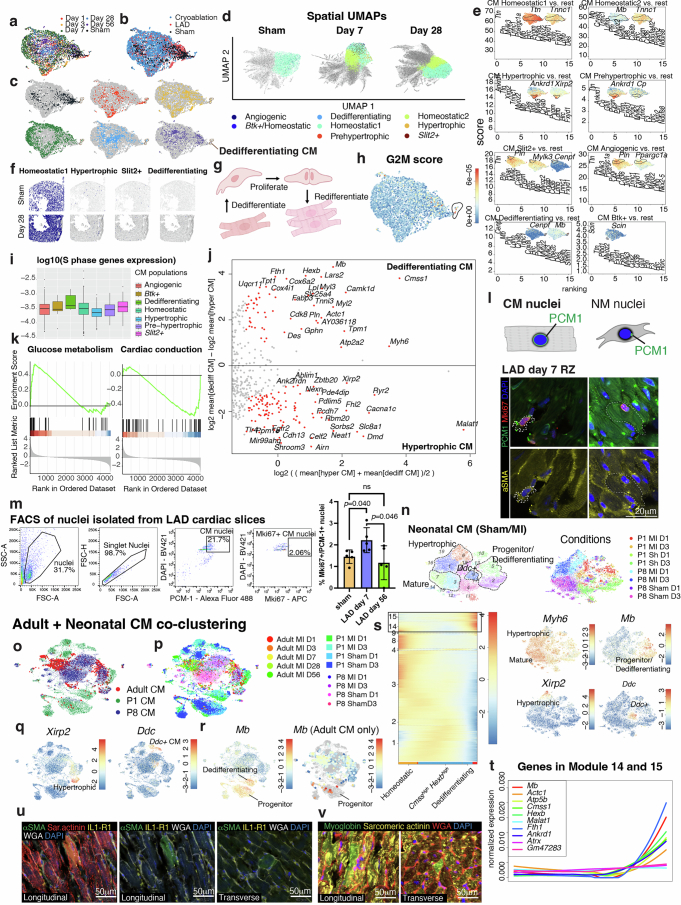
Comparison of dedifferentiating and hypertrophic CM. **a and b**, UMAP of CM populations highlighting time point post-lesion (**a**) or surgery type (**b**). **c**, UMAP of CM populations at separate time points post-lesion. **d**, Spatial UMAP showing all NiCo-annotated CM subtypes. **e**, Top 15 DEGs of NiCo-annotated CM subtypes in the spatial data. In each plot, expression of one or more DEG(s) is also plotted in the corresponding scRNA-seq UMAP(s), showing enrichments of the same DEGs in the same cell subtype across scRNA-seq and spatial data, indicating confident annotations of these cell subtypes. **f**, Spatial maps of major CM subtypes in day 28 and sham hearts. **g**, Schematic diagram showing de-differentiation-proliferation-re-differentiation trajectory of CM upon MI. **h**, UMAP of CM population showing G2M scores. Circled cells are the de-differentiating CM. **i**, Quantification of log_1__0_-transformed summed S phase cell cycle gene expression (Methods) across all CM subtypes. One nucleus/point. Box center, median. Box upper and lower bounds, 25 and 75 percentiles. Whisker maxima, 75 percentile + 1.5 interquartile. Whisker minima, 25 percentile - 1.5 interquartile. From left to right, n = 1170, 14, 16, 2260, 452, 87 and 139 nuclei. **j**, Differential gene expression analysis between de-differentiating and hypertrophic CM. Significant genes are indicated in red (negative binomial Benjamini–Hochberg adjusted *p* < 0.05). **k**, Gene set enrichment analysis comparing de-differentiating and hypertrophic CM. **l**, Upper panel, schematic diagram showing the localization of PCM1 signal in CM versus NM nuclei. PCM1 specifically labels the nuclear membrane of CM but not NM. Lower panels, IF staining of LAD day 7 heart RZ sections with PCM1, Mki67 and αSMA. Mki67 labels proliferating nuclei, and αSMA labels de-differentiating CM. n = 2 experiments. **m**, FACS analysis of the isolated nuclei from day 3 sham, day 7 LAD and day 56 LAD cardiac tissues. Quantification of the percentage of proliferating CM (DAPI^+^ PCM1^+^ Mki67^+^ nuclei) against all CM is plotted in the right panel. Statistics, unpaired two-tailed t-test for two experimental groups. ns, not significant. Error bars, standard deviation centered at mean. n = 5 biological replicates. **n**, Reanalysis of the published neonatal post-MI CM datasets from Cui et al. (2020)^4^. Left, UMAP showing clustering of CM subtypes. Right, UMAP showing condition of CM samples. D1, day 1 post-MI/sham. D3, day 3 post-MI/sham. Bottom, expression UMAPs of gene markers for mature (*Myh6*^*high*^), progenitor/dedifferentiating (*Mb*^*high*^), hypertrophic (*Xirp2*^+^ ) and Ddc^+^ (*Ddc*^+^ ) CM subtypes. **o**, UMAP showing co-clustering of our adult CM dataset (labeled in red) with neonatal post-MI CM datasets. P1 hearts are regenerative (green) while P8 hearts are non-regenerative (blue). **p**, UMAP showing detailed CM conditions. **q**and **r**, Marker genes annotating hypertrophic (*Xirp2*^+^ ) and *Ddc*^+^ CM, which do not contribute to heart regeneration (**q**), and progenitor and de-differentiating CM (*Mb*^*high*^), which are the main contributors to heart regeneration (**r**). Right panel in (**r**), same UMAP where only adult CM are shown. Adult de-differentiating CM (*Mb*^*high*^) co-cluster with P1 CM progenitor cells. **s**, FateID pseudotime expression profiles (Methods). Heatmap showing gene expression profiles grouped into 14 modules. Highlighted modules (14 and 15) are genes with gradually increasing expression across the de-differentiation trajectory. **t**, Pseudotime expression profiles of example genes from modules 14 and 15. **u**and **v**, IF staining of day 7 LAD cardiac slices in the RZ. De-differentiating CM are identified by Sarcomeric actinin^+^ aSMA^+^ and IL-1R1^+^ (**u**) or Sarcomeric actinin^+^ Myoglobin^+^ (**v**). WGA labels cell membranes. n = 2 experiments. Source data

Hearts without the ability to regrow lost CMs are unable to fully regenerate. The regenerative capacity of CMs is limited in adult mammals, resulting in hypertrophy of the remaining CMs and fibrosis after wounding. Our CM snRNA-seq data (Fig. 7a–d and Extended Data Fig. 9a–c) encompass large proportions of pre-hypertrophic (*Myh7*^*+*^*Ankrd1*^*+*^) and hypertrophic (*Xirp2*^*+*^*Nppa*^+^*Nppb*^+^*Ankrd1*^+^*Myh7*^*+*^) CM states on days 1 and 3, followed by a transient increase in angiogenic and *Slit2*^*+*^ states on day 7, expressing angiogenic ligands, growth factors and patterning signals such as *Angpt1*, *Fgf12*, *Cntn2*, *Slit2* and *Slit3*. In the spatial data, the homeostatic and (pre-)hypertrophic states were recovered (Extended Data Fig. 9d–f). Most of these annotated states were distributed across the BZ and RZ (but excluded from the IZ/FZ), with enrichment of hypertrophic CMs in the BZ (Fig. 7e). Our data also revealed a rare population, annotated as de-differentiating CMs, which was absent in sham (Extended Data Fig. 9c) and shared several markers with hypertrophic CMs, for example, *Myh7* and *Ankrd1*; however, this population upregulated progenitor genes (*Actc1*, *Mb* and *Mdh2*), metabolic genes (*Cox6a2* and *Atp5e*) and cell cycle genes, indicating a progenitor-like, proliferation-active state (Fig. 7a–d and Extended Data Fig. 9g–i). Unlike hypertrophic CMs, de-differentiating CMs were sparsely present yet not highly enriched in the BZ, but predominantly localized toward the endocardial region (Fig. 7e). They exhibit increased glucose metabolism, but decreased cardiac conduction gene expression (Extended Data Fig. 9j,k). IF staining of the day 7 LAD heart with the CM nuclear membrane marker PCM1, the proliferation marker Mki67 and the CM progenitor marker αSMA^4^, confirmed the presence of de-differentiating and proliferating CMs in the RZ. Most of these cells exhibited smaller cell volumes and more elongated morphology than surrounding CMs (Fig. 7f and Extended Data Fig. 9l). To determine the temporal dynamics of CM proliferation, the percentage of CMs (PCM1^*+*^ nuclei) with proliferative activity (Mki67^*+*^) in sham, LAD day 7 and day 56 were quantified by FACS analysis. Compared to sham, day 7 LAD CMs showed increased proliferative activity, which returned to basal levels at day 56, suggesting a transient response (Extended Data Fig. 9m).

Neonatal mice on postnatal day 1 (P1) can regenerate their myocardium after MI, with the emergence of progenitor or de-differentiating CM subtypes^4^ represented by a *Mb*^*high*^*Myh6*^*low*^ population specific to P1 but absent from P8 hearts post-MI (Extended Data Fig. 9n). By integrating our data with this neonatal CM dataset, we confirmed the resemblance of our adult de-differentiating CM to neonatal progenitor CM (Extended Data Fig. 9o–r). Pseudotime analysis of neonatal post-MI CMs revealed transient activation into a de-differentiated state, followed by re-differentiation/conversion of these cells back to the homeostatic state (Fig. 7g). In contrast, adult CMs entered the de-differentiated state via a *Cmss1*^*high*^ *Hexb*^*high*^ state, but the re-differentiation path is absent (Fig. 7g and Extended Data Fig. 9s,t), suggesting that adult CMs may only partially de-differentiate without the full capacity to give rise to mature CMs.

**Extended Data Fig. 10:**
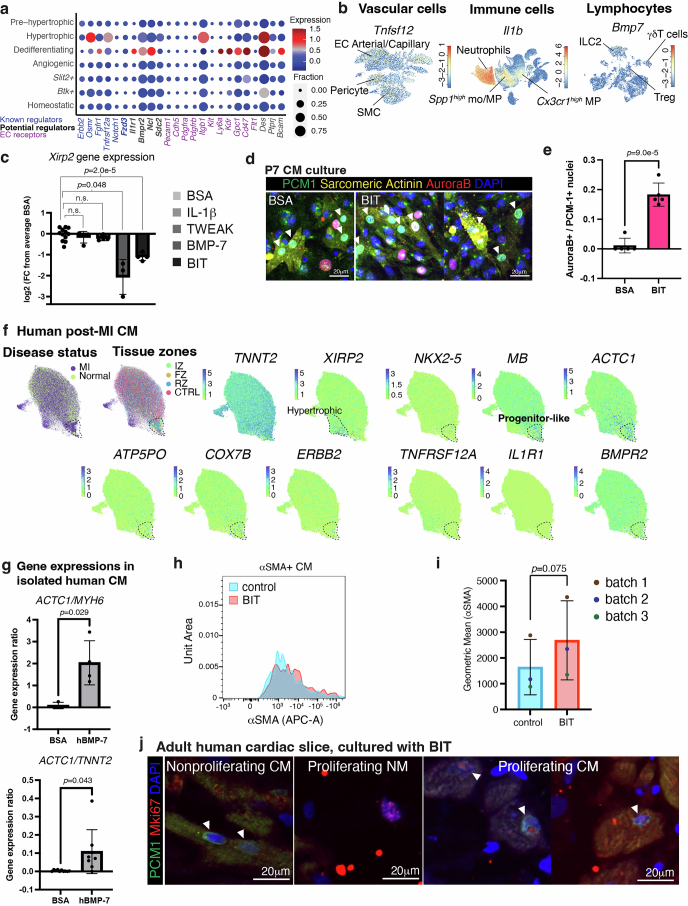
Gene program and activation of mouse and human CM dedifferentiation. **a**, Dot plot showing expression of candidate de-differentiation-promoting receptor genes in CM subtypes. Dot size indicates fraction of cells expressing the gene. Dot color indicates normalized expression level. **b**, UMAPs showing expression of candidate de-differentiation-promoting ligand genes in vascular cells (left), all immune cells (middle) and lymphocytes (right). **c**, qRT-PCR of *Xirp2* (hypertrophic CM marker gene) in cultured CM after ligand exposure. log_2_-transformed relative gene expression fold changes against BSA control are plotted. From left to right, n = 12, 4, 4, 3 and 4 biological replicates. Unpaired two-tailed t-test was performed in each group against BSA control. **d**, Primary P7-CM culturing (Methods). Cells were exposed to ligands for 48 hours. Representative IF images of ligand-exposed CM. CM (Sacromeric actinin+ and nuclear PCM1^+^) with AuroraB^+^ DAPI^+^ nuclei exhibit cell cycle activity. White arrows point to CM nuclei. **e**, Image quantification of CM with cell cycle activity. n = 5 biological replicates. **f**, Human post-MI CM UMAP from snRNA-seq data by Kuppe et al. (2022)^6^ deposited in CELLxGENE. Marker genes for general CM (*TNNT2*), hypertrophic CM (*XIRP2*), and dedifferentiating CM (*NKX2-5, MB, ACTC1, ATP5PO* and *COX7B*) are plotted. Receptor genes identified in the mouse CM de-differentiation niche are also plotted (*TNFRSF12A, IL1R1* and *BMPR2*). **g**, qRT-PCR of *ACTC1* (CM progenitor marker gene), *MYH6* and *TNNT2* (mature CM marker genes) in primary human CM progenitor-like cells after hBMP-7 exposure. *ACTC1*-to-*MYH6* and *ACTC1*-to-*TNNT2* gene expression ratios (as a progenitor state score) were compared against BSA control. *ACTC/MYH6*, n = 4 biological replicates. ACTC1/TNNT2, n = 8 biological replicates. **h**and **i**, αSMA expression in human cardiac slice CM. Representative FACS histogram showing the intensities of αSMA in control (BSA-treated) and BIT-treated CM (sarcomeric actinin^+^) (**h**), and the quantifications of the signal intensities (**i**), n = 3 biological replicates. **j**, Human cardiac slice culture (Methods). Representative images of BIT-exposed CM undergoing cell cycle re-entry, as determined by the presence of PCM1^+^Mki67^+^ nuclei. n = 2 experiments. Statistics for (**c**,**e**,**g**,**i**), unpaired two-tailed t-test for two experimental groups. ns, not significant. Source data

Next, we investigated potential niche drivers of CM de-differentiation. All of our day 7 spatial samples revealed proximity of de-differentiating CMs to ILC2, T_reg_ cells, CD8^+^ T cells, B cells, *Timd4*^+^ rMPs, IFN^+^ myoFBs and arterial ECs (Fig. 7h,i and Supplementary Figs. 8 and 9). IF confirmed the presence of niches containing de-differentiating CMs (sarcomeric actinin^+^ αSMA^+^/myoglobin^high^), ECs (CD31^+^) and ILC2 (BMP-7^+^GATA3^+^) in both the BZ and RZ (Fig. 7i–k, and Extended Data Fig. 9u,v). These neighboring cell types may provide the ligands to drive CMs toward de-differentiation. Although individual roles of some ligands in promoting the CM cell cycle were reported in different animal models, their cellular origins as well as their combined effects have remained unknown^34–36^. Arterial ECs express the TNFRSF12A ligand TNFSF12 (TWEAK), ILC2 specifically express BMP-7 binding to BMPR2, and neutrophils, which were not captured in the spatial data but were highly abundant in the day-1 scRNA-seq data, express IL-1β together with mo/MPs that bind to IL-1R1 expressed in de-differentiating CMs (Extended Data Fig. 10a,b and Supplementary Fig. 11). IF confirmed IL-1R1 protein expression in de-differentiating CMs (Fig. 7k and Extended Data Fig. 9u).

To functionally assess the role of these ligands in CM cell fate determination, primary CMs isolated from P7 mouse LV were exposed to recombinant mouse IL-1β, TWEAK and BMP-7. As CMs are mostly nonregenerative in P7 hearts^4^, CM proliferation was almost absent when primary CMs were exposed to bovine serum albumin (BSA) as a negative control (<2% actinin^+^ nuclei were Mki67^+^AuroraB^+^). In comparison, exposure to IL-1β, TWEAK or BMP-7 individually induced a significant (two- to fivefold) increase in CM cell cycle activity (Fig. 7l,m). Additionally, qRT–PCR revealed a significantly higher expression of the CM progenitor marker *Actc1* relative to the mature CM marker *Myh6* (Fig. 7n), and sarcomeric actinin structures showed a tendency to disassemble upon de-differentiation and cell cycle. Lower expression of the hypertrophic marker *Xirp2* was observed when exposed to BMP-7 (Extended Data Fig. 10c), suggesting induction of de-differentiation and suppression of hypertrophy. Notably, although the individual effect of each of these ligands on promoting the full CM cell cycle was marginal, simultaneous exposure of CMs to all three ligands (BIT) resulted in synergistic induction of *Actc1* and proliferation with a tenfold increase in Mki67^+^AuroraB^+^ CM nuclei and a significant increase in cytokinetic CMs (*P* < 0.01) (Fig. 7m,n). To further ensure that the proliferating cells are indeed CMs, the CM nuclear marker PCM1 was stained together with sarcomeric actinin, AuroraB and DAPI. Quantification of the AuroraB nuclear signal confirmed a significant increase of cycling CMs upon BIT exposure, consistent with our data with sarcomeric actinin alone as a CM marker (Extended Data Fig. 10d,e). Taken together, these data suggest that the interaction of CMs within a niche hosting ILC2, ECs and neutrophils could potentially induce de-differentiation by cosignaling via the BMP-7–BMPR2, IL-1β–IL-1R1 and TWEAK–TNFRSF12A axes.

To further elucidate whether the role of these ligands is conserved in humans, we inspected the presence of de-differentiating CMs in human post-MI snRNA-seq data^6^. Indeed, a small fraction of CMs, found in the RZ and IZ, expressed progenitor-like genes, such as *MB*, *ACTC1*, *NKX2-5*, *ATP5PO* and *COX7B*, as in our adult mouse data. These cells are transcriptionally distinct from hypertrophic CMs (*XIRP2*^+^) and are almost absent in the control group, supporting the occurrence of de-differentiation (Extended Data Fig. 10f). For the candidate receptors, human CMs in general express low levels of *TNFRSF12A* and *IL1R1*, but higher levels of *BMPR2*. Culturing primary human CM progenitor-like cells (isolated from ventricles of failing adult human donor heart) with recombinant hBMP-7 ligand strongly induced the progenitor marker *ACTC1* (Extended Data Fig. 10g). To test whether our ligands can induce CM de-differentiation in native human myocardium, we utilized an ex vivo culturing system for cardiac slices excised from explanted hearts of adult human transplant recipients. The slices were cultured in biomimetic chambers and exposed to both preload and regular electrical stimulations. BSA (control) or the candidate ligands (BIT) were added to the culture, and slices were collected after 6 days (Fig. 7o). Exposure to BIT ligands led to reduction in actinin structures (Fig. 7p) and increased αSMA expression in CMs (Extended Data Fig. 10h,i), as determined by IF and FACS, respectively. While the control slices contained no Mki67^+^ proliferative cells, BIT-exposed slices exhibited cell proliferation events for CMs, as determined by the presence of sarcomeric actinin^+^/PCM1^+^Mki67^+^ nuclei (Fig. 7q,r and Extended Data Fig. 10j). In conclusion, CM de-differentiation niche factors could serve conserved functional roles in both mouse and human myocardium, potentially driving CM cellular plasticity upon cardiac injury.

## Discussion

After MI in the adult heart, replacement of infarcted myocardium with scar tissue achieves a balance between mechanical support to prevent tissue dilatation on the one hand and the inevitable impairment of cardiac physiological function due to the loss of electromechanically functional myocardium on the other hand.

Achieving this balance involves complex cellular communication networks that coordinate the proper responses of multiple cell types across tissue locations and stages of remodeling. Currently available cardiac single-cell sequencing and spatial datasets provide insights into cell type-specific responses^6,37–39^, but typically lack coverage of the dynamics of interacting cell types in the lesion. Moreover, most murine MI studies have collected the entire LV for sequencing, diluting the representation of relevant cell types from the lesion area in the dataset. Available spatial transcriptomic data were generated with low-resolution sequencing-based techniques. Although deconvolution can be applied to infer the cell type composition of each pixel^40^, more subtle cell state modulation and covariation of gene expression between cell types within the same spot cannot be inferred. Thus, there is a need for a comprehensive post-lesion cardiac atlas that maps spatiotemporal dynamics of cell type architecture in the wound and thereby supports identification of the key heterocellular interactions that shape the outcome of cardiac remodeling.

By densely sampling all stages of post-lesion remodeling, collecting tissue from the lesion area and enriching rare cell populations, we were able to overcome previous limitations and create an atlas with unprecedented resolution of the cell types involved in cardiac tissue repair. We not only captured the behavior of cell types known to play a critical role in scar formation (such as FBs and MPs), but we also uncovered the dynamics of poorly characterized rare cell types, such as unconventional T cells, innate lymphocytes, SwCs and de-differentiating CMs. These discoveries open the door to more in-depth studies that will improve our understanding of the role of these cell types after lesioning or in homeostatic hearts. In this study, we also compared the cellular responses across the two common cardiac injury models that mimic human MI, Cryo and LAD, and found similar cell type dynamics and gene expression responses in our single cell and nucleus data. While spatial analysis was restricted to Cryo we provide evidence for the presence of the key cell–cell interactions highlighted in this study in LAD from published LAD low-resolution spatial data (Visium)^41^ (Supplementary Fig. 12). The similar damage response we observed for Cryo and LAD suggests that both models are suitable for investigating the role of these cell types in cardiac healing.

**Fig. 8:**
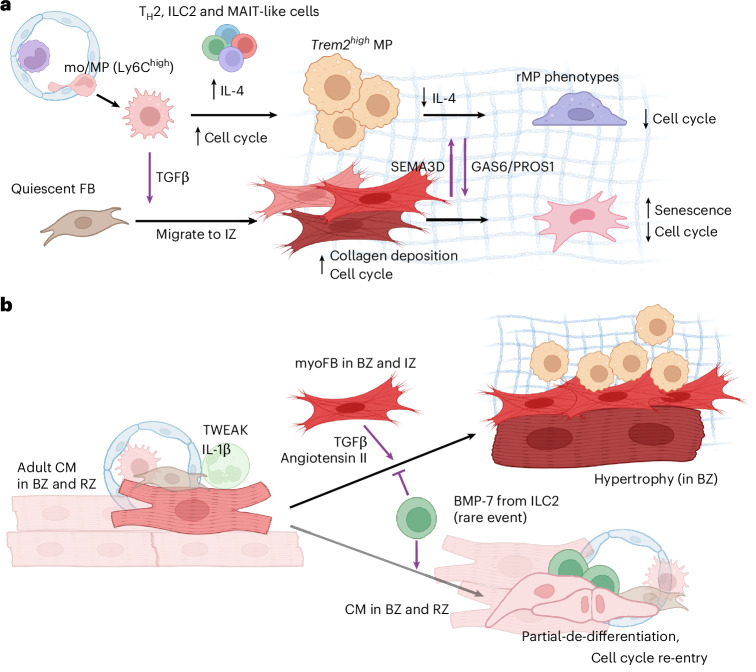
Spatiotemporal niches in cardiac wound healing. **a**, Time-dependent interactions of MPs, lymphocytes and FBs in the fibrotic niche. At days 1–7 post-lesion, cMPs promote differentiation of quiescent FBs (qFB) and *Ccl2*^+^ FB into myoFBs via TGFβ. On day 7, cMPs gradually acquire an rMP phenotype via a transitional *Trem2*^*high*^ state, through the transient exposure to IL-4 secreted from lymphocytes, for example T_H_2, ILC2 and MAIT-like cells. Transitional MPs and rMPs suppress proliferation of myoFBs through GAS6 and PROS1. Reciprocally, myoFBs dampen proliferation of rMPs through SEMA3D. **b**, Niche of hypertrophic and de-differentiating CMs. In the BZ early after lesion, adult CMs colocalize with migrating myoFBs, which induce CM hypertrophy through TGFβ1. Rare synergistic interaction of CMs with myeloid cells, ILC2 and ECs/FBs, which provide IL-1β, BMP-7 and TWEAK, respectively, results in CM de-differentiation. Individually, BMP-7, IL-1β and TWEAK provide marginal pro-proliferative effects. BMP-7 further prevents CMs from acquiring a hypertrophic phenotype. The synergistic effect of these interactions promotes CM de-differentiation and cell cycle re-entry.

The integration of sequencing data with single-cell resolution spatial transcriptomics through our NiCo algorithm^17^ enabled the identification of signaling interactions in lesion niches and their downstream effects on cell states. Our spatiotemporal analysis identified an immune-fibrotic niche in the IZ, composed of multiple mo/MP subtypes, myoFBs and lymphocytes. T_H_2, ILC2 and MAIT-like cells produce IL-4 on day 3–7, which promotes M1 (Ly6C^high/mid^) MP transdifferentiation into *Spp1*^*high*^*/Trem2*^*high*^ states. At later stages, consistent with decreased lymphocyte numbers, IL-4 signaling is not required for further transdifferentiation of *Trem2*^*high*^ MPs into resident-like phenotypes. Additionally, both the intermediate (*Trem2*^*high*^) and resident (*Cx3cr1*^*high*^) MPs interact with myoFBs in the IZ/FZ, promoting mutual suppression of proliferation in the maturing scar through GAS6/PROS1 and SEMA3 signaling (Fig. 8a).

GAS6 and PROS1 are vitamin K-dependent proteins that bind TAM receptors, including TYRO3, AXL and MER^42^. GAS6 is generally known as a mitogenic ligand^32,43^, whereas an opposing role has been reported for PROS1 (ref. ^44^). Consistently, we observed that GAS6 confers proliferation activation at low concentrations; however, both ligands mediated cell cycle suppression at high concentrations in vitro, in accordance with reduced FB proliferation in vivo on day 7 and beyond. This mechanism could limit fibrosis in the mature scar, while allowing non-activated FB replenishment in the distal zones, where the FB–MP interaction is sparse.

Our data further revealed niche interactions that support CM de-differentiation (Fig. 8b), a poorly understood process with great potential to promote functional recovery of the heart. Unlike hypertrophic CMs, which are highly enriched in the BZ, de-differentiating CMs were sparse throughout the myocardium, consistent with previous studies^5,45,46^. While angiotensin II and TGFβ from myoFBs is known to induce CM hypertrophy^47,48^, our data indicate that the simultaneous activation of multiple signaling pathways (BMP-7, TWEAK and IL-1β) is required to synergistically push CMs into the de-differentiated state. ILC2 are the main source of BMP-7 in the de-differentiation niche but are very rare in the adult myocardium. γδT and T_reg_ cells also express BMP-7 but occur at low frequency in the adult myocardium, and do not show significant spatial interactions with CMs. Our findings reveal that remnant regenerative mechanisms persist in adult mammals and underscore the critical role of lymphocytes, particularly unconventional subsets, in supporting potential cardiac regeneration.

Of note, understanding how to promote effective CM de-differentiation may confer substantial therapeutic potential for the restoration of adult cardiac function post-MI in the future. Through our experimental data on isolated human primary CMs and ex vivo cultured human cardiac slices, we have demonstrated that our identified niche ligands may promote human CM de-differentiation.

We acknowledge that the absence of direct systolic function measurements represents a limitation of our study. While our correlative analysis of EF dynamics and pathway activity provides supportive insights, it cannot establish causality. The causal relationship between our identified pathways and the systolic function could be addressed in future studies.

In conclusion, this spatiotemporal cell type atlas for cardiac injury identifies key heterocellular interactions in the repair process, and constitutes a valuable resource for future studies, leading to identification of additional therapeutic targets to improve post-MI recovery in humans.

## Methods

### Animal experiments

All animal experiments were carried out according to the guidelines in Directive 2010/63/EU of the European Parliament on the protection of animals used for scientific purposes; they were approved by the local authorities in Baden–Württemberg, Germany (Regierungspräsidium Freiburg, G21-129) and by the animal welfare officer of the Centre for Experimental Models and Transgenic Services-Freiburg (X21-03R; X23-03R). All animals were kept on a 12-h day–night cycle (6:00 to 18:00), temperature at 20–24 °C and humidity at 45–46%. Holding conditions were approved by the local authorities in Freiburg, Germany, in accordance with the German animal welfare regulations.

### Mouse cardiac surgery

LV cryoablation (Cryo), ischemia-reperfusion injury after LAD ligation and sham surgery were performed on female wild-type C57BL/6J mice at 12 weeks of age. For each injury model and sham, at least five mice were performed per time point of collection. Surgery and Cryo were performed as previously described^49^. In brief, mice were anesthetized by intraperitoneal injection of 80–100 µl of anesthesia solution (20 mg ml^−1^ ketamine (Ketaset; Zoetis), 1.4 mg ml^−1^ xylazine hydrochloride (0.12% Rompun; Bayer), in 114 mM NaCl (0.67% m/v; B. Braun Melsungen)). After induction of deep anesthesia, eye ointment (Bepanthen containing 50 mg ml^−1^ dexpanthenol; Bayer) was applied, 500 µl glucose solution (278 mM glucose, 5% (m/v); B. Braun Melsungen) was injected intraperitoneally and 250 µl of analgesia solution (10 µg ml^−1^ buprenorphine (Temgesic; Indivior) in 154 mM NaCl (0.9% m/v, B. Braun Melsungen)) was injected subcutaneously into the neck. Mice were shaved on the left side of the thorax (precordial region) and on the right leg, placed on a warming platform of a small animal physiology monitoring system (Harvard Apparatus) and the front extremities were fixed with tape. A rectal thermometer was inserted to allow regulation of the heating platform to ensure a body temperature of 37 °C. Thermometer and tail were fixed with tape. Mice were intubated for positive pressure ventilation (Kent Scientific; 40% O_2_, 120 breathing cycles per min). Isoflurane was supplied at 5% until the animal stopped spontaneous respiratory movements, and then reduced to 2.0–2.5%. An infrared blood oximeter was attached to the right leg to track hemoglobin oxygen saturation, ventilation was adjusted if needed. The surgical field was disinfected using Softasept N (B. Braun Melsungen). Skin and muscles were cut along the third intercostal space, and a rib spreader was used to separate the ribs. The pericardium was cut, and the epicardial surface was dry-blotted using a cellulose pad.

For mice undergoing ventricular cryoablation, a hexagonal metal probe (stainless steel, 2.5-mm edge-normal distance) was prechilled in liquid nitrogen and applied for 8–10 s to the free LV mid-wall, positioned to avoid major coronary vessels. After retraction of the probe, the time until the tissue regained a deep-red color was recorded (consistently within 5–10 s). For mice in which the ischemia-reperfusion injury was performed, the LAD was identified and ligated with an 8-0 suture, while a narrow plastic tubing was inserted below the suture. Successful ligation was indicated by the infarcted area changing in color, from red to gray. After 30–45 min, the tubing and suture were removed. For sham surgery, application of the metal probe and ligation was not performed. The rib spreader was removed, and the thorax closed using a 6-0 silk suture around the third and fourth ribs (4–5 single knots). Before final closure, any remaining air was removed from the thorax using a small cannula. Isoflurane application was stopped, and the skin was closed with a 4-0 silk suture. Once the mouse started breathing spontaneously, intubation and fixation were terminated, and the mouse was transferred to a heated and oxygenated wake-up chamber. Analgesia was maintained for 72 h post-surgery via twice-daily subcutaneous injection of 250 µl of buprenorphine (10 µg ml^−1^ in 154 mM NaCl, injected in the morning and late afternoon). During the night, buprenorphine was also supplied via the drinking water (10 µg ml^−1^ buprenorphine (Subutex lingual tablets, Indivior) in 20 mM glucose solution).

### Tissue processing and cell isolation

The tissue collection and slicing protocol was adapted from a previously published protocol^49,50^. In brief, mice received sodium–heparin solution (16 U g^−1^ body weight) by intraperitoneal injection, before being sacrificed by cervical dislocation 5 min later. Their chests were then opened and their hearts were excised. To wash out the blood from the heart, hearts were cannulated and flushed with cold ‘cutting solution’ containing 138 mM NaCl, 0.33 mM NaH_2_PO_4_, 5.4 mM KCl, 2 mM MgCl_2_, 10 mM HEPES, 10 mM glucose, 0.5 mM CaCl_2_ and 30 mM 2,3-butanedione 2-monoxime (BDM) and the pH was adjusted to 7.3 with 1 M NaOH at 37 °C, osmolality 330 ± 10 mOsm l^−1^. Tissue blocks of the LV free wall containing the post-Cryo or post-LAD lesion center (identified as a region appearing mostly white), BZ and adjacent myocardium (or corresponding tissue areas from sham animals) were excised and embedded in low-melting-point agarose (4% (m/v)) at 37 °C and then put on ice. Agarose blocks were glued to the stage of a precision vibratome and cut into 300-µm thick slices (60 Hz cutting frequency and 1.5 Hz amplitude; model 7000smz-2; Campden Instruments). The resulting tissue slices were subjected to chemical fixation, cryopreservation or cell isolation.

For chemical fixation, slices were exposed for 30 min to 4% paraformaldehyde (PFA)-containing phosphate-buffered saline (PBS) at room temperature (RT), washed three times in PBS and then stored in PBS at 4 °C.

To cryopreserve tissue, each slice was placed flat in the bottom center of a cryomold (Tissue-Tek, 4566), immersed in a thin layer of optimal cutting temperature compound (Tissue-Tek, 4583). To fix the orientation and keep the slice flattened, an additional cryomold of the same size was placed on top of the tissue, before snap-freezing in liquid nitrogen. Frozen slices were stored at −80 °C until cryosectioning.

For cell isolation, tissue slices were collected from five different LVs of the same surgery model and time point, and were stored at 4 °C in cutting solution and warmed to RT before initiating cell isolation. Tissue slices were transferred to ‘enzymatic solution’ containing 20 mM KCl, 10 mM KH_2_PO_4_, 2 mM MgCl_2_, 20 mM Taurin, 10 mM glucose, 100 mM L-glutamic acid potassium salt monohydrate and 30 mM BDM, at an osmolality of 310 ± 10, then the pH was adjusted to 7.3 with KOH at 37 °C. Tissue was digested for 12 min with 0.5 mg ml^−1^ proteinase K (Sigma-Aldrich) and further digested with 0.23 mg ml^−1^ Liberase TL Research Grade with 5 μM CaCl_2_(Hoffmann-La Roche) for up to 45 min. To remove large tissue fragments, the cell suspension was filtered through a nylon mesh (pore size, 1 × 1 mm).

For spatial transcriptomics experiments, following flushing with cold cutting solution, hearts were fixed in 4% PFA at 4 °C for 24 h. Tissues were then transported in 70% ethanol for at least 24 h and subjected to stepwise rehydration to PBS. This was followed by tissue clearance and dehydration as per the standard formalin-fixed paraffin-embedding protocol. Paraffin-embedded tissues were stored at 4 °C.

### NM antibody labeling and FACS sorting

FACS sorting was performed using a BD FACS Symphony S6 or FACSAria III Cell Sorter (BD BioSciences). The same gating and collection strategy was applied across all time points post lesioning to ensure comparable cell type ratios. For each of the collected NM sample for 10x GEM generation, isolated cells were first blocked by the TruStain FcX Fc blocking antibody (BioLegend, 101319) for 15 min at 4 °C. Cells were labeled for 30 min at 4 °C in the dark with fluorophore-conjugated antibodies against various surface markers, including CD45-Pacific Blue (BioLegend, 103125), Ter119-APC/Cy7 (BioLegend, 116223), CD31-PE (BioLegend, 102507), CD146-PE (BioLegend, 134703), CD14-FITC (BioLegend, 123307) and CD11c-APC (BioLegend, 117309), together with the Zombie-NIR viability dye (BioLegend, 423105), which labels dead cells. All antibodies were diluted 1:100 in FACS buffer (PBS with 10% fetal bovine serum (FBS; VWR, MDTC35-016-CV) containing 1% penicillin–streptomycin solution (P/S; 100 U ml^−1^, Gibco, 15140122)). When performing FACS gating, half the cells were collected from the Zombie-NIR and Ter119-negative gate (unbiased) and the other half were collected from the enrichment gates (Extended Data Fig. 1a). Cells were sorted into FACS buffer-coated 1.5-ml tubes, each with 40 μl of FACS buffer with 1:1,000 diluted Murine RNase Inhibitor (New England Biolabs, M0314L). After collection, cell counting was performed. In each sample, 10,000 cells from the unbiased tube and 10,000 cells from the enrichment tube were pooled together for 10x GEM generation.

### Single nucleus isolation from mouse hearts

For nucleus isolation, vibratome-cut cardiac slices were placed in MACS M-tubes (Miltenyi Biotec, 130-093-236), containing 1 ml of nuclear staining buffer (PBS with 1% BSA (Miltenyi Biotec, 130-091-376) and 1:500 diluted (0.2 U μl^−1^) Murine RNase Inhibitor, pre-cooled to 4 °C). The tube was placed into a gentleMACS Octo dissociator (Miltenyi Biotec, 130-096-427), and then dissociated using the included ‘Protein_01.01 M-tube’ protocol. The M-tubes then underwent pulse-spinning, were flushed with 1 ml of nuclear staining buffer, and were filtered through a 40-μm cell strainer (Falcon, 1172689). The eluates were collected in 50-ml Falcon tubes, which were then centrifuged for 5 min at 500*g*, 4 °C and brake 5. Supernatants were removed and pellets were resuspended in 500 μl of nuclear staining buffer.

### Nuclei antibody labeling and FACS sorting

Isolated nuclei from different time points were first labeled with 1:100 diluted αPCM1 antibody (MERCK, HPA023370) in nuclear staining buffer for 5 min at 4 °C (not on ice), then with 1:100 diluted fluorophore-conjugated anti-rabbit IgG secondary antibody (BioLegend, 406419) for an additional 25 min at 4 °C in the dark. Samples then underwent centrifugation (5 min, 500*g*, 4 °C and brake 5), followed by multiplexing with CellPlex reagents according to the manufacturer’s instruction (10x Genomics, PN-1000261), resulting in time point-specific labeling in each sample. After labeling, nuclei were washed with 2 ml nuclear staining buffer and centrifuged (5 min, 500*g*, 4 °C and brake 5). Labeled nuclei from all time points were then pooled together in 1 ml nuclear staining buffer containing 10 μg ml^−1^ Hoechst 33,342 (Thermo Fisher Scientific, H3570) and underwent FACS sorting to enrich for Hoechst^+^PCM1^+^ nuclei. Enriched nuclei were collected in a 1.5-ml tube precoated with nuclear staining buffer, containing 150 μl of nuclear staining buffer with 1:100 diluted of Murine RNase Inhibitor. Within the collected nuclei, 10 μl was stained with Trypan Blue solution (Thermo Fisher, 15250061) at a ratio of 1:1, and was visually inspected under a light microscope (20x objective) to assess the nuclei shape and morphology. If the majority of nuclei seemed to be intact (with no visible disruption of nuclear membrane), this was followed by nuclear counting. A total of 20,000 nuclei were used for 10x GEM generation.

### Sample processing for sequencing

In each sample, up to 20,000 sorted cells were loaded into GEM Chip (10x Genomics, PN-1000127) and encapsulated into emulsion droplets using the Chromium Controller (10x Genomics) according to the manufacturer’s instruction. cDNA library generation and amplification were performed using the Chromium Next GEM Single Cell 3ʹ Reagent kits v.3.1 (Dual Index) protocol according to manufacturer’s instructions (10x Genomics, PN-1000268 and PN-1000190). The prepared cDNA library was then sequenced on the Nextseq2000 platform (Illumina). The Cell Ranger v.6.1.1. pipeline was used to generate a digital gene expression matrix starting from raw data. For alignment and quantification of gene expression the reference transcriptome has been built using the mouse mm10-2020-A as reference genome. De-multiplexing of CellPlex barcodes into individual samples was performed by Seurat^40^.

### scRNA-seq and snRNA-seq data analysis

Single-cell clustering and data analysis were performed using VarID2 (ref. ^51^). Each sample condition was initially represented by one raw gene count matrix. Cells within each matrix were labeled with their condition. The labeled matrices were merged and processed into a single cell object via the SCseq function. Only cells with more than 1,000 unique molecular identifier (UMI) counts were considered for clustering and analysis. Mitochondrial genes, ribosomal genes and predicted genes with Gm-identifier were filtered (FGenes argument in filterdata function). Analysis of *k*-nearest neighbors was performed using the ‘pruneknn’ function. For the clustering combining all cells across all conditions, batch effect correction was performed using the harmony package^52^. In summary, the following settings were used: large=TRUE, regNB=TRUE, knn=25, no_cores=8, seed=12345, FSelect=TRUE, batch=batches, bmethod=harmony. To define clusters, the ‘graphCluster’ function was applied, with Leiden clustering^53^. Settings were as follows: pvalue = 0.01, use.weights=TRUE, use.leiden=TRUE, leiden.resolution=1. Gene expression values were normalized by correcting variability associated with total transcript count per cell by a negative binomial regression^51^, which could be visualized in heatmaps of gene expression Uniform Manifold Approximation and Projection (UMAP). For Boxplot gene expression representation, UMI counts were normalized by dividing transcript counts in each cell by the total transcript count per cell before multiplying by the minimum total transcript count across all cells. For cell type annotations, two schemes were applied throughout this study. The first scheme defined all identified cell types from the main clusters (of all cell types) in detail, and annotated all subtypes and states within each major cell types (such as all lymphocyte populations and all FB cell states), provided that each annotated cell (sub)type had distinct marker gene expressions. Known cell (sub)types were annotated according to published names, and newly identified types were annotated based on their enriched marker gene(s). This is referred to as the ‘detailed annotation scheme’ and yielded 78 cell types in total (Supplementary Table 1). Alternatively, a ‘simplified annotation scheme’ was also defined, where some cellular subtypes without distinctive separations in UMAPs were pooled together to form one major type, resulting in only 38 cell types (Supplementary Table 1). The simplified annotation scheme was only used for the spatial neighborhood interaction analysis. To analyze specific cell types in a higher resolution, cell identities from clusters of CMs, FBs, vascular cells, immune cells and SwCs were collected. The raw UMI counts from these cells were extracted from the count matrices and underwent re-clustering, with the same parameters described above. Within the immune cell subclusters, cells from all lymphocyte clusters (except B cells) were collected, and further re-clustered into a lymphocyte-only subcluster.

### Differential gene expression analysis

Differential expression analysis was computed with the ‘diffexpnb’ function of the RaceID3 (v.0.2.5) algorithm. Detection of DEGs between specific groups of cells was performed with a method similar to that previously reported^54^. In brief, a negative binomial distribution, which captures the gene expression variability for each group of cells, was inferred based on a background model of the expected transcript count variability estimated by RaceID3 (ref. ^55^). Based on the inferred distributions, a *P* value for the significance of the transcript counts between the two groups of cells was estimated and multiple testing was corrected for using the Benjamini–Hochberg method.

### Pathway enrichment analysis and gene-concept network plots

Pathway enrichment analysis was performed with the ‘enrichPathway’ function from the ReactomePA R package^56^, with a *P* value cutoff of 0.05. Inputs were ENTREZ gene IDs of genes selected by differential expression analysis or otherwise specified. Gene-concept networks were constructed using the enriched pathways from the two cell populations under comparison. Pathways were located at the center node of each cluster, while the corresponding genes were shown as connected smaller nodes. The size of each pathway node corresponded to the number of contributing genes. For the comparison between *Il5*^+^ ILC2 and other ILC2 subtypes, differential gene expression analysis was first computed with the ‘diffexpnb’ function of the RaceID3 (v.0.2.5) algorithm. Genes of log_2_-foldchange > 0.3 were collected and matched against the enrichPathway database. The ‘cneplot’ function was applied to construct the plot.

### Differentially enriched pathways analysis

DEGs (adjusted *P* < 0.05) across the two groups (LAD and Cryo) were obtained for the analysis. Differential expression analysis was computed with the ‘diffexpnb’ function of the RaceID3 (v0.2.5) algorithm. Pathway comparison was then performed using the fast gene set enrichment analysis package^57^. Selected genes were used to match against pathways from the ‘examplePathways’ or ‘REpathways’ reference databases. Maximum and minimum pathway gene number (maxSize and minSize) were 15 and 500, respectively. For plotting, the top 15 pathways from each group are displayed.

### Cell cycle gene activity analysis

S phase and G2M phase gene lists are given in VarID2 (cc_genes$s and cc_genes$g2m, respectively). The summed expressions of the S or G2M phase genes in the desired condition were calculated and visualized in barplots.

### Lineage trajectory inference

Transition probabilities between two clusters were calculated as the geometric mean of the individual link probabilities connecting the two clusters. To order cells pseudo-temporally, the Slingshot method^58^ was applied on a desired dimensionally reduced RaceID object (selected cell clusters). Using the FateID package, pseudotime expression profiles were derived by self-organizing maps and grouped into modules.

### Ligand–receptor interaction analysis

To predict potential ligand–receptor interactions using the single-cell and nucleus datasets, the CellChat package^59^ was applied. In brief, this tool first identified enriched ligand and receptor genes across each manually annotated cell type (*P* < 0.05). A detailed annotation scheme (as defined above) was used for interaction analysis. Using CellChat’s own ligand–receptor database, predicted interactions between defined cell types, conditions (time points) and pathways were visualized via the ‘netVisual_bubble’ function. This dot plot shows the communication probability (indicated by the color of each dot) and the corresponding *P* value (the size of each dot) for each predicted interaction.

### Reanalysis of published human MI snRNA-seq datasets

Seurat objects from three post-MI patient IZ samples were downloaded from Zenodo at https://zenodo.org/records/6578047 (ref. ^60^) and annotated as ‘IZ_P21_ext1’, ‘IZ_P22_ext2’ and ‘IZ_P23_ext3’. After clusters of myeloid cells and FB were confirmed by marker genes, corresponding cells were re-clustered by VarID2 (ref. ^51^) with the same parameters as above. For the FB subtype UMAP construction (compumap), the parameters used were spread=6 and min_dist=0.5. For the myeloid cell subtype UMAP construction, the parameters used were spread=1 and min_dist=0.5.

### snRNA-seq data comparison with published neonatal mouse MI datasets

The neonatal MI CM atlas^4^ was downloaded from NCBI Gene Expression Omnibus, with accession number GSE130699. In brief, the count matrices from all time points (P1 mice MI, 1 and 3 days; P1 mice sham, 1 and 3 days; P8 mice MI, 1 and 3 days; and P8 mice sham, 1 and 3 days) were collected, with cells from each condition being labeled. The resulting matrices, together with our adult CM post-cryoablation count matrices, underwent clustering by VarID2 (ref. ^51^), with the same settings and parameters as described above. For the UMAP construction (compumap), the parameters used were min_dist=3.5 and spread=4.

### Spatial transcriptomics gene panel design

The Xenium Mouse Tissue Atlassing Panel (379 genes) was used and topped up with 96 custom genes based on DEGs of each cell (sub)type identified in our scRNA-seq data. The probes had two complementary sequences to bind the target RNA and contained a third region with a gene-specific barcode. This allowed the ends to ligate into a circular DNA probe for in situ amplification, ensuring high specificity by preventing ligation during off-target binding.

### Spatial transcriptomics sample processing, imaging and preprocessing

All experimental steps followed the guidelines of the Xenium workflow from 10x Genomics^61^ (CG000580, CG000749 and CG000584). In brief, 5-μm formalin-fixed paraffin-embedded tissue sections (transverse plane of the LV) were mounted on a Xenium slide (PN-1000465) within the desired imaging region. After deparaffinization, slides were placed in Xenium cassettes (PN 3000951) and messenger RNA was exposed by decrosslinking. A gene panel, as described above, was applied to the sample in 10 nM, targeting mRNA molecules of the 475 target genes. In addition to the gene panel, two types of negative control probes were included to assess nonspecific binding and to ensure that the observed signals originated from RNA, not genomic DNA. Hybridization was performed at 50 °C overnight for 20 h, followed by a washing step to remove unhybridized probes.

Ligation was performed at 37 °C for 2 h to circularize the stably bound probes (ligating the 5’ and 3’ ends), followed by rolling circle amplification (RCA) amplification at 30 °C for 2 h. This resulted in amplification of the gene-specific barcodes in each RNA-bound probe, enhancing the signal-to-noise ratio. After post amplification wash, samples were sequentially treated with ethanol 70%, 100%, 70% and PBS-Tween, blocked for 1 h at RT with ‘Block and Stain Buffer’ (PN 2001083) and incubated overnight (18 h) with ‘Multi-Tissue Stain Mix’ from the manufacturer’s cell segmentation add-on kit to label cell boundaries (ATP1A1, E-cadherin and CD45), as well as intracellular proteins (αSMA, Vimentin) and 18S rRNA marker. After PBS-Tween washes and stain enhancement (PN 2000992), background fluorescence was chemically quenched followed by nuclear staining with 4′,6-diamidino-2-phenylindole (DAPI).

Slides were loaded onto the Xenium Analyzer (software v.3.2.1.2) for region selection, with each region corresponding to one tissue section. The instrument combines a high numerical aperture and a fast area scan camera with a low read noise sensor (achieving a lateral resolution of 200 nm per pixel). After selection, images were acquired in sequential hybridization-imaging cycles. In each cycle, unique fluorescently labeled secondary probes targeting RCA-amplified probe-RNA complexes were hybridized, and visualized in combination with DAPI staining. After imaging, secondary probe was stripped, and subsequent rounds of hybridization and imaging were performed. Z-stacks were acquired at a 0.75-μm step size across the tissue thickness.

Images were pre-processed with the built-in analysis software Xenium v.3.2.0.7, in which low quality signals were filtered out, and the transcript identities were decoded using the Xenium codebook. For quality control, each decoded transcript was assigned a Phred-style Q-Score reflecting confidence in transcript identity. Q-Scores were calibrated using negative control codewords, probes targeting non-biological sequences, and unassigned codewords. Only transcripts with a Q-Score ≥20 were included in downstream analyses. Cell type-specific analysis has shown that our spatial data has higher transcript capturing efficiency than sc/snRNA-seq (ranging from 1.09–6.4 folds), especially among CMs, FBs and immune compartments (Supplementary Fig. 13).

### Spatial transcriptomics post-processing of samples

After completion of spatial transcriptomics experiments, tissue sections were transported in glycerin under a coverslip at 4 °C. Upon arrival, tissues were incubated in Hanks’ Balanced Salt Solution (HBSS, 14025-100, Thermo Fisher), after which the coverslip was removed. After quencher removal, tissues were then blocked in 3% BSA in HBSS for 30 min at RT to minimize nonspecific binding. To enhance CM segmentation (Supplementary Fig. 14), tissues were stained with the following wheatgerm agglutinin (WGA, dilution 1:40, BOT-29026-1, Biozol), DAPI, mouse CD14 (dilution: 1:400, 60253-1-IG, Proteintech), rabbit RPS9 (dilution 1:100, PA5-104493, Invitrogen), anti-mouse Alexa Fluor 568 (dilution 1:1,000, A11077, Invitrogen), anti-rabbit Alexa Fluor 488 (dilution 1:1,000, A11055, Invitrogen). The Vector TrueVIEW kit (Vector, VEC-SP-8500, Biozol) was used for quenching autofluorescence followed by mounting. Tissues were imaged with the IXplore SpinSR Olympus super resolution imaging system (Evident) with a ×20 objective.

### Spatial transcriptomics integration of multiplexed immunofluorescence images and cell segmentation

We used the elastix based package WsiReg2D for image registration via affine and solid transformation based on the DAPI staining of the cell segmentation staining of the Xenium workflow and our customized staining panel. A combined composite image was used for finetuning the pre-trained light microscopy model of MicroSAM. The resulting model was used for cell segmentation. Xenium ranger was used for integration of cell segmentation with the spatial transcriptomics data.

### Spatial transcriptomics data analysis

We utilized the NiCo algorithm^17^to perform integrative analysis of spatial transcriptomics and scRNA-seq/snRNA-seq data.

#### Spatial cell type annotation

We used all the default parameters for the cell type annotation task, except for the spatial guiding Leiden clustering resolution, which was set to 0.35 or 0.8, to maximize the number of annotated cell types.

#### Spatial neighborhood composition analysis

For NiCo-based neighborhood analysis, the cellular ‘niche’ is defined as a local neighborhood comprising a center cell and its spatially adjacent cells of immediate contact (radius *R* = 0). This effectively captures direct physical neighbors in the tissue, approximating biological multicellular units such as microenvironments or cellular clusters. For interaction analysis, NiCo predicts over-representation of colocalizing cell types, normalized by their overall cellular frequencies in the tissue. In more detail, analysis was conducted for each cell type with the NiCo interaction module, determining colocalization scores for all niche cell types using a logistic regression classifier. A simplified cell type annotation scheme was applied for plotting. An edge weight threshold value of 0.14 was used to plot the cell type niche interaction networks.

#### Niche covariation analysis

Analysis was conducted for each cell type independently with the covariation module of NiCo using integrated NMF to identify cell type-specific latent factors. NiCo can be used to conduct ridge regression between the latent factors of the central cell type as dependent variables and latent factors of colocalized neighborhood cell types as independent variables. Significant regression coefficients reflect statistical dependence, or covariation, of latent factors belonging to colocalized cell types. We used the default parameters for the identification of ligand–receptor pairs associated with covarying cell type-specific latent factors, and performed pathway enrichment analysis for all genes correlating to each latent factor to infer the associated gene programs.

### Mouse primary CM culturing

Neonatal mouse hearts were collected from P7 mice (at least six hearts were collected for a single experiment) and were transferred to a 6-cm Petri dish (VWR, 734-0006, 353003) containing PBS. Remaining blood was pumped out of the hearts by compression, using forceps to apply gentle pressure. Remaining nonventricular tissue was removed and the ventricles were minced into small fragments using dissecting scissors. For tissue digestion, a Neonatal Heart Dissociation kit (Miltenyi Biotec, 130-098-373) was used. In brief, PBS was replaced by 2.5 ml of enzyme mix (prepared according to the manufacturer’s instruction). The Petri dish was then incubated at 37 °C with gentle agitation (65 rpm, Incu-Shaker Mini, Z763578-1EA) for 45 min. To enhance dissociation rate, the tissue-enzyme mix was pipetted 20 times through a wide-bore 1,000-µl pipette tube every 15 min. After completion of the digestion process, 5 ml FACS buffer was added and the mixture was then passed through a 70-µm cell strainer (Falcon, 352350). Filtered cells were collected in a 50-ml Falcon tube, which was centrifuged at 350*g* for 5 min at 4 °C. The supernatant was removed, and the pellet was resuspended with 2 ml of ACK lysis buffer (Biozym, 882090-FFM) for 5 min at 4 °C, followed by the addition of 3 ml of FACS buffer and re-centrifugation. To magnetically label NMs (not CMs), the pellet was resuspended with 100 µl of 1:10 FACS buffer-diluted neonatal cardiomyocyte isolation antibody cocktail (Miltenyi, 130-100-825) for 15 min at 4 °C in the dark. For magnetic selection, the mixture was topped up with 500 µl FACS buffer, and then loaded into an MS column (Miltenyi Biotec, 130-042-201) in a MACS magnetic separator (Miltenyi Biotec, 130-042-102), pre-loaded with FACS buffer. The column was washed with 3× 500 µl FACS buffer. All the flow-through was collected and combined. Collected cells (CMs) were counted. A total of 10,000 CMs per well were seeded onto a 96-well plate (Thermo Fisher, 164588) precoated with 0.1% Galectin (InSCREENex, INS-SU-1015-50ml) in 200 µl full IMDM medium (composed of IMDM (Gibco, 12440053) supplemented with 10% FBS, 1× MEM Non-Essential Amino Acids Solution (NEAA; Gibco, 11140050) and 1% P/S solution). After plating, CMs were incubated at 37 °C in a 5% CO_2_ cell culture incubator overnight. The next day, medium was replaced by full IMDM medium supplemented with 100 ng ml^−1^ of either Recombinant Mouse IL-1β (BioLegend, 575102), Recombinant Human TWEAK (BioLegend, 566402), Recombinant Mouse BMP-7 Protein (R&D Systems, 5666-BP-010/CF) or BSA. Cells were incubated at 37 °C, 5% CO_2_ for 48 h and were collected for downstream quantification.

### Mouse primary cardiac FB and myoFB culturing

Ventricles were collected from 6-week-old mice. Ventricular tissue was minced into small fragments using dissecting scissors. Heart extraction and cell isolation followed the adult heart dissociation protocol described above. To enrich cardiac FBs, MojoSort was performed. In brief, isolated cells were centrifuged (300*g*, 5 min at 4 °C), resuspended in 1:100 FACS buffer-diluted biotin-conjugated CD140a antibody (Thermo Fisher, 13-1401-82) and incubated for 15 min at 4 °C. Cells were then centrifuged (300*g*, 5 min at 4 °C), after which the supernatant was discarded and 100 µl of MojoSort Streptavidin Nanobeads (1:10 diluted in FACS buffer, BioLegend, 480016) was added to the mixture; this was then incubated for another 15 min at 4 °C in the dark. After incubation, the mixture was transferred to a 5-ml FACS tube, and inserted into a MojoSort Magnet (BioLegend, 480019), pre-cooled with ice, for 5 min. The supernatant was discarded. The tube was removed from the magnet, and the cells remaining in the tube were resuspended with 0.5 ml FACS buffer. After cell counting, the cell solution was diluted by adding full DMEM medium (DMEM (DMEM, high glucose, GlutaMAX Supplement and pyruvate; Gibco, 31966021), 10% FBS and 1% P/S solution) and seeded onto a 96-well plate, with 10,000 cells per well in 200 µl medium. After plating, cells were incubated at 37 °C in a 5% CO_2_ cell culture incubator overnight. The next day, the medium was removed and cells were washed twice with PBS, before being incubated with serum-free DMEM with Recombinant Mouse GAS6 protein (0.05–1,000 ng ml^−1^, R&D Systems, 986-GS-025/CF), Recombinant Mouse Protein S/PROS1 (100 ng ml^−1^, R&D Systems, 9740-PS-050/CF) or an equivalent amount of BSA. Cells were incubated at 37 °C in 5% CO_2_ for 48 h and were then collected for downstream quantification. For the myoFB culturing experiments, plated FBs were first activated for differentiation into myoFBs using 100 ng ml^−1^ TGFβ overnight in medium with 10% FBS, followed by recombinant mouse GAS6 or BSA exposure in serum-free and TGFβ-free medium for 48 h. Cells were then collected for downstream quantification, including IF, qPCR and FACS analysis.

### Human primary cardiac FB culturing

Human cardiac fibroblasts (HCFs) were purchased from Promocell (C-12375), having been isolated from the ventricles of adult human hearts and cryopreserved. Cells were defrosted, resuspended in full HCF medium (Growth Medium 3 with supplements, Promocell, C-23025) and allowed to grow in a T75 flask (Merck, C7231-120EA) until 80% confluence. HCFs were then trypsinized using 3 ml of trypsin–EDTA (0.25%) with phenol red (Gibco, 25200072) for 5 min at 37 °C, followed by centrifugation (300*g*, 5 min, 4 °C) and pellet resuspension by HCF medium. After cell counting, cells were plated in a 96-well plate, with 5,000 cells per well in 200 µl medium. Thereafter, cells were incubated at 37 °C in a 5% CO_2_ cell culture incubator overnight. For human cardiac myoFB generation, cells were exposed to 0.1 µg ml^−1^ of Recombinant Human TGF-β1 (BioLegend, 781802). The next day, the medium was removed. Cells were washed twice with PBS and then incubated with either serum-free DMEM, with the addition of Recombinant Human Gas6 protein (R&D Systems, 885-GSB-050) or the equivalent amount of BSA. Cells were incubated at 37 °C, 5% CO_2_ for 48 h and were collected for RNA extraction. For the HCF culturing assays that were used for IF imaging and quantification, HCFs were seeded at only 1,000 cells per well to maintain a low density throughout the culturing process. Activation of HCFs by TGF-β1 was carried as mentioned above. On the following day, the activated cells were cultured in serum-free DMEM with various concentrations of hGAS6 proteins (0, 0.0, 0.1, 0.2, 0.5 and 1 µg ml^−1^, respectively) for 2 days, followed by cell fixation and antibody staining for IF imaging.

### Human primary progenitor-like CM culturing

Human progenitor-like CM isolated from the ventricles of adult human hearts were purchased from Promocell (C-12810). Cells were defrosted, resuspended in full myocyte growth medium with supplements (Promocell, C-22070) and allowed to grow in 37 °C, 5% CO_2_ until 80% confluency. After medium removal, CMs were then trypsinized using 3 ml of trypsin–EDTA (0.25%) with phenol red (Gibco, 25200072) for 5 min at 37 °C, followed by centrifugation (300*g*, 5 min at 4 °C) and pellet resuspension in myocyte growth medium. After cell counting, cells were seeded into a 96-well plate, with 10,000 cells per well in 200 µl medium. After plating, cells were incubated at 37 °C in a 5% CO_2_ cell culture incubator overnight. The next day, cells were washed with fresh medium with 0.1 µg ml^−1^ recombinant human BMP-7 protein (BioLegend, 595601) or an equivalent amount of BSA. Cells were incubated at 37 °C, 5% CO_2_ for 48 h and then collected for downstream quantification.

### Generation of danger associated molecular patterns from murine heart

For the generation of DAMPs, murine hearts from C57BL/6 mice were collected and pulverized in a mortar under liquid nitrogen. The powder was then resuspended in PBS supplemented with protease inhibitor cocktail at a concentration recommended by the manufacturer (Sigma, 4693159001), using three hearts per ml. DAMPs were stored at −20 °C until further use.

### T cell activation and differentiation

The differentiation of T_H_2 cells was performed as previously described^62^. In brief, CD4^+^ T cells were isolated from lymph nodes and spleen by negative selection using the MojoSort Mouse CD4 T cells isolation kit (BioLegend, 480033). T cells were cultured in modified RPMI 1640 medium with physiological glucose concentration (100 mg dl^−1^) by diluting RPMI 1640 standard medium (Gibco, 11875093) with glucose-free RPMI medium (Roth, 9094.1). The medium was supplemented with 10% fetal calf serum (Sigma, 12133), 100 U ml^−1^ penicillin (Gibco, 15140122), 100 U ml^−1^ streptomycin (Gibco, 15140122) and 1% GlutaMAX-I (Gibco, 350050061). After isolation, cells were plated on a delta-surface plate (Nunc, 150628), which was precoated with 12 μg ml^−1^ polyclonal anti-hamster IgG (MP Biomedicals, 0856984) for a minimum of 2 h and washed once with PBS. On a 12-well plate, 1 × 10^6^ cells were activated with 0.5 μg ml^−1^ of anti-CD3 (clone 145-2C1, BioXCell, BE0001-1), 1 μg ml^−1^ anti-CD28 (clone 37.51, BioXCell, BE0015-1), 5 μg ml^−1^ anti-IFNγ (clone XMG1.2, Thermo Scientific, 315-05), 10 ng ml^−1^ rhIL-2 (Thermo Scientific, 200-02) and 50 ng ml^−1^ rmIL-4 (Thermo Scientific, AF-214-14) for 4 days, with replating and adjusting the concentration to 1 × 10^6^ cells per well at day 3. Then, 50% of medium was renewed every day and cells were kept at 37 °C in 5% CO_2_ throughout culturing.

### BMDM culture and polarization

Bone marrow was isolated as described previously^63^. In brief, bone-marrow cells were isolated as follows. The femur and tibia bones of C57BL/6 mice were flushed with cold PBS. Cells were centrifuged, and the pellets were resuspended in single-cell suspension in MP medium containing DMEM (Gibco, 41965-039) supplemented with 10% fetal calf serum (Sigma, 12133), 100 U ml^−1^ penicillin (Gibco, 15140122), 100 U ml^−1^ streptomycin (Gibco, 15140122), 1 mM L-glutamine (Gibco, 25030081), 1× non-essential amino acids (Gibco, 11140-035), 1× sodium pyruvate (Gibco, 11360-070) and 10–15% L929 supernatant (SN). L929 SN was generated as previously described^64^, with the necessary concentration determined via bioassays. Nonadherent progenitors of MPs were collected after 24 h of cultivation and seeded at a concentration of 0.5 × 106 ml^−1^ for 6 days in MP medium. Then, 50% of medium was renewed every other day.

For MP cell fate conversion experiments, BMDMs were polarized with 1% DAMPs and 50 ng ml^−1^ IFNγ in MP medium for 24 h. After 24 h, the medium was removed and exchanged with MP medium containing 10 ng ml^−1^ IL-4, T_H_2 cell cultured supernatant or T_H_2 cells separated by Transwells. After 48 h, the medium and/or T_H_2 cells were removed again, and cells were treated with un-supplemented MP medium for 24 or 48 h. Throughout culturing, cells were kept at 37 °C in 5% CO_2_.

### Flow cytometry analysis with Cytek

Flow cytometry analysis was performed as previously described^65^. Briefly, the cells were incubated on ice with Zombie-NIR (BioLegend, 423105) for 10 min in PBS. After washing with PBS containing 0.5% BSA, cells were stained with fluorophore-conjugated antibodies (Supplementary Table 6) for surface antigens for 35 min on ice in PBS containing 0.5% BSA. Cells were then fixed with Foxp3/TF staining buffer following the manufacturer’s recommendations (eBioscience, 00-5523-00) for 35 min on ice. For intracellular staining, cells were incubated with fluorophore-conjugated antibodies targeting intracellular antigens for 35 min at RT in 1× permeabilization buffer (eBioscience, 00-8333-56). Sample data acquisition was performed at the Aurora Flow Cytometer (Cytek), and analyzed with Tree Star (FlowJo) and Prism (GraphPad Software).

### SEMA3D assay

Upon differentiation, murine BMDMs were treated for 72 h with 100 ng ml^−1^ SEMA3D (R&D Systems, 9386-S3-025/CF) in PBS and PBS containing 0.5% BSA as a control, respectively. Cells were fixed in 4% PFA, washed, and treated with 1× ReadyProbes Mouse-on-Mouse IgG Blocking Solution (Invitrogen, R37621) according to the manufacturer’s instruction. Mouse anti-histone H3 antibody (Abcam, ab14955, 1:100) was then incubated overnight at 4 °C. Cells were washed and goat anti-mouse IgG Alexa Fluor 647 secondary antibody (Invitrogen, A32728, 1:1,000) was incubated for 1 h at RT. Nuclei staining was performed with Hoechst 33342 according to the manufacturer’s instructions. Fluorescence intensity measurements were performed on a Leica TCS SP8 X inverted confocal microscope (SCI-MED imaging facility).

### Human cardiac slices culture

Transmural LV human cardiac samples (*n* = 2) were obtained from failing hearts during LV assist device implantation (LVAD), according to protocols approved by the institutional ethics committees of the University of Erlangen-Nürnberg and the University of Leipzig. Studies followed the guidelines of the extended Declaration of Helsinki. All patients (or their legal guardians) gave their written informed consent.

Preparation and culture of LV slices was carried out as previously described^66,67^. M199 (Sigma, M4530) was used as a culture medium and supplemented with β-mercaptoethanol (50 μM), 1:100 P/S (Biochrom, A2213), cortisol (50 nmol l^−1^), insulin (10 ng ml^−1^), transferrin (5.5 μg ml^−1^) and selenite (6.7 ng ml^−1^). Cardiac slices were mounted into specialized culture chambers (MyoDish, MD-1.2, InVitroSys) and diastolic force was set to 1.5 mN. Slices were continuously paced at 0.5 Hz by electrical field stimulation. Medium was partially exchanged every 48 h. After 16 days of culture, slices were randomly divided into two groups. The control group (*n* = 5) was treated with 1% FBS. The BIT group (*n* = 6) was treated with a combination of human recombinant BMP-7, human recombinant TWEAK, and human recombinant IL-1β at a concentration of 0.1 µg ml^−1^ each. Accounting for 0.2 ml of evaporated volume, 1.6 ml of medium was removed every 48 h and 1.8 ml of fresh medium containing the three substances or FBS was added. After 6 days of treatment, the slices were fixed in 4% PFA solution for 45 min and then stored in PBS at 4 °C.

### Multiplexing immunohistochemistry and immunofluorescence staining

For IF staining of mouse cultured CM or cardiac FB cultures, cells on 96-well plates were first washed with PBS, then fixed with 4% PFA in PBS (Sigma-Aldrich, 252549) for 15 min at RT, followed by two 5-min washes with 0.3% Triton X-100 in PBS. Cells were then exposed to 70% ethanol at −20 °C for 1–2 h. Cells were then returned to RT, washed with PBS (twice, for 5 min each time) and blocked in blocking solution (3% BSA and 0.0025% Triton X-100 in PBS) for 1 h, followed by antibody labeling (1:200 diluted rat Mki67-FITC antibody; BioLegend, 151211). For FB staining, 1:100 diluted rat CD140a-PE (Thermo Fisher, 12-1401-81) and 1:250 diluted mouse αSMA primary antibody (Invitrogen, 14-9760-80) were added. For CM staining, 1:200 diluted human actinin (sarcomeric)-PE (Miltenyi, 130-123-996) and 1:400 diluted mouse AuroraB primary antibody (BD Bioscience, 611082) were added at 4 °C in the dark for 16–24 h. Cells were washed twice with PBS for 10 min, and then incubated with 1:400 goat anti-mouse IgG-AF647 (BioLegend, 405322) and DAPI solution for 2 h at RT in the dark, followed by two 10-min washes with PBS. For HCF staining, Mki67-FITC and αSMA primary antibody were used at the same concentrations. For IHC staining of post-lesion cardiac slices, mounted cryosections underwent similar fixation, wash, and antibody incubation steps as for IF. The following antibodies were used: mouse PDGFRa (1:100 dilution, R&D Systems, AF1062-SP), rat CD45 (1:200 dilution, BioLegend, 103108), rat CD11b (1:500 dilution, Invitrogen, MA5-17857), goat GAS6 (1:200 dilution, R&D Systems, AF986-SP), rabbit PROS1 (1:200 dilution Invitrogen, PA5-106880), goat AXL (1:60 dilution, R&D Systems, AF854-SP), mouse BMP-7 (1:200 dilution, NovusBio, NBP2-52425), rat GATA3 (1:100 dilution, Invitrogen, 14-9966-82) and goat KIT (1:40 dilution, R&D Systems, AF1356-SP). For multiplexing, tissues were first imaged after labeling with the first round of antibodies, followed by two rounds of antibody stripping by glycine–HCl buffer (0.1 M glycine hydrochloride (Merck, G2879-100G) and 0.1% Triton X-100, pH 2.2) for 15 min. Tissues were then washed with PBS for 5 min twice, and then were re-incubated with another round of antibodies.

### Spinning disk confocal imaging and analysis

IXplore SpinSR Olympus super resolution imaging system (Evident) was used for imaging of IF and IHC samples, with a 20x objective. Image acquisition was performed with the cellSens software (Evident). 405, 488, 561 and 640 nm lasers were used to excite DAPI, FITC, PE and AF647 fluorophores, respectively. The acquisition time was 200 ms for each color channel. After acquisition, images were analyzed using Fiji ImageJ, including adjustments to brightness and contrast, followed by cell density quantifications.

### Flow cytometry analysis with FACSCelesta for isolated cardiac FB

Isolated cells well placed in 1.5-ml tubes and centrifuged (350*g*, 5 min at 4 °C) to remove supernatants. Pellets were resuspended in 4% PFA in PBS, and were fixed for 10 min at RT, followed by centrifugation (800*g*, 5 min at 4 °C) to remove the fixative. Cells were washed with PBS containing 0.1% Triton X-100, recentrifuged and then resuspended in DAPI-containing solution (1 μg ml^−1^) for 10 min in the dark, recentrifuged and resuspended with PBS in FACS tubes (Falcon, 38025). Readout of DNA content was performed by BD FACSCelesta Cell Analyzer (BD BioSciences), where histograms of DAPI signal intensities across all cells were recorded. Quantitative analysis of the DNA content was performed with FlowJo v.10 software.

### RNA extraction, cDNA synthesis and quantitative PCR

RNA extraction and purification were performed with the PicoPure RNA Isolation kit (Applied Biosystems, KIT0204) according to the manufacturer’s instructions. cDNA synthesis was performed with RevertAid H Minus First Strand cDNA Synthesis kit (Thermo Scientific, K1632), with the following components: 4 μl RNA, 1 μl Random Hexamer, 7 μL H_2_O, 4 μl 5× Reaction Buffer, 1 μl RiboLock RNase Inhibitor, 2 μl dNTP mix and 1 μl RevertAid H Minus Reverse Transcriptase. The reaction was performed in thermocycler under the following conditions: 25 °C for 5 min; 42 °C for 60 min; 70 °C for 5 min; 4 °C on hold. qPCR of the cDNA samples were performed as follows: 2 μl 1:4 diluted cDNA, 5 μL iTaq Universal SYBR Green Supermix (Bio-Rad, 1725120), 0.6 μl 10 μM forward and reverse primers (Supplementary Table 7 provides primer sequences) and 2.4 μl nuclease-free H_2_O. For mouse and human cardiac FB samples, *Gapdh* was used as a reference gene. For human cardiac FB samples, *GAPDH* was used as a reference gene. For mouse CM samples, *Smchd1* was used as a reference gene as *Gapdh* exhibited variation in expression levels across different subtypes in the snRNA-seq data, whereas expression of *Smchd1* was more consistent across all CM subtypes. The qPCR reaction was performed with CFX Connect Real-Time PCR Detection System (Bio-Rad, 1855201) for 45 amplification cycles.

### Quantification and statistical analysis

All experiments were performed using randomly assigned mice. Statistical significance was calculated by unpaired two-tailed *t*-tests for two experimental groups or one-way analysis of variance for multiple comparisons. Statistical analyses and quantifications were performed in Excel (Microsoft Corporation), Prism 10 (GraphPad Software), FlowJo (FlowJo) and Fiji ImageJ. Figures display mean ± s.d. as indicated. *P* < 0.05 was considered statistically significant.

### Reporting summary

Further information on research design is available in the Nature Portfolio Reporting Summary linked to this article.

## Supplementary information

Supplementary Information Supplementary Figs. 1–14.

Reporting Summary

Supplementary Tables 1–7 Supplementary Table 1. Cell type-specific marker genes that are used for defining the cell types in the detailed and simplified cell type annotation schemes. Supplementary Table 2. Top ten differentially expressed genes of all annotated cell types (detailed annotation scheme) in the scRNA-seq data. Gene names, expression values, fold changes and *P* values are included. Supplementary Table 3. Xenium mouse panel for this study, with 475 genes, composed of ‘general’ and ‘custom’ probe sets. Supplementary Table 4. Differentially expressed genes of the spatial cell types at day 7 post-lesion, under the simplified annotation scheme. The top 25 differentially expressed genes for each NiCo-annotated cell type are displayed. Supplementary Table 5. Differentially expressed genes of the spatial cell types at day 7 post-lesion, under the detailed annotation scheme. The top 25 differentially expressed genes for each NiCo-annotated cell type are displayed. Supplementary Table 6. Cell surface antibodies used to characterize cell fates of MP in the cell fate conversion experiments. This includes the host species, source, identifier and catalog number. Supplementary Table 7. Primers for the qRT–PCR experiments. This includes the sequences, template strands, length, start and end sites of the target genes, GC content, melting temperature and degree of self-complementary.

## Source data

Source Data Fig. 1 Raw statistical quantifications of the Xenium spatial transcriptomic data.

Source Data Fig. 4 Raw statistical quantifications of the MP transdifferentiation FACS data.

Source Data Fig. 5 Raw statistical quantifications of the FB–MP co-culture.

Source Data Fig. 6 Raw statistical quantifications of FB GAS6 and MP SEMA3D cultures.

Source Data Fig. 7 Raw statistical quantifications of the CM culture and human cardiac slice experiments.

Source Data Extended Data Fig. 7 Raw statistical quantifications of the macrophage transdifferentiation experiment (FACS surface protein signal intensity across different time points).

Source Data Extended Data Fig. 8 Raw statistical quantifications of the FB GAS6/PROS1 culture experiments (IF and FACS quantifications), senescence marker quantifications and human cardiac fibroblast culture experiments.

Source Data Extended Data Fig. 9 Raw statistical quantifications of mouse post-LAD CM nuclei Mki67 expression over time.

Source Data Extended Data Fig. 10 Raw statistical quantifications of the mouse CM, human CM and human cardiac slices.

## Data Availability

Single-cell RNA-seq data have been deposited at the Gene Expression Omnibus under accession numbers GSE280373 and GSE280376. The sc/snRNA-seq and spatial transcriptomics datasets are available for exploration and visualization at https://www.wuesi.medizin.uni-wuerzburg.de/cardiac_spatiotemporal_atlas/.

## Code availability

Source code used in this study is available on GitHub at https://github.com/Andyson-Chan/myocardial_infarction/tree/main.
