# Supplementary material for: Spatiotemporal dynamics of the cardioimmune niche during lesion repair
Source: Nat Cardiovasc Res. 2025 Nov 3;4(11):1550–72. doi: 10.1038/s44161-025-00739-6 (PMC12611762; doi:10.1038/s44161-025-00739-6)
Supplement: Supplementary file 1 — Supplementary Information Supplementary Figs. 1–14. [file 44161_2025_739_MOESM1_ESM.pdf]

nature cardiovascular research

Supplementary information https://doi.org/10.1038/s44161-025-00739-6

Spatiotemporal dynamics of the cardioimmune niche during lesion repair

In the format provided by the authors and unedited

Table of content

Supplementary Figure 1....2
Supplementary Figure 2....3
Supplementary Figure 3....5
Supplementary Figure 4....8
Supplementary Figure 5....10
Supplementary Figure 6....11
Supplementary Figure 7....12
Supplementary Figure 8....14
Supplementary Figure 9....16
Supplementary Figure 10....18
Supplementary Figure 11....19
Supplementary Figure 12....20
Supplementary Figure 13....22
Supplementary Figure 14....23

Supplementary Figure 1

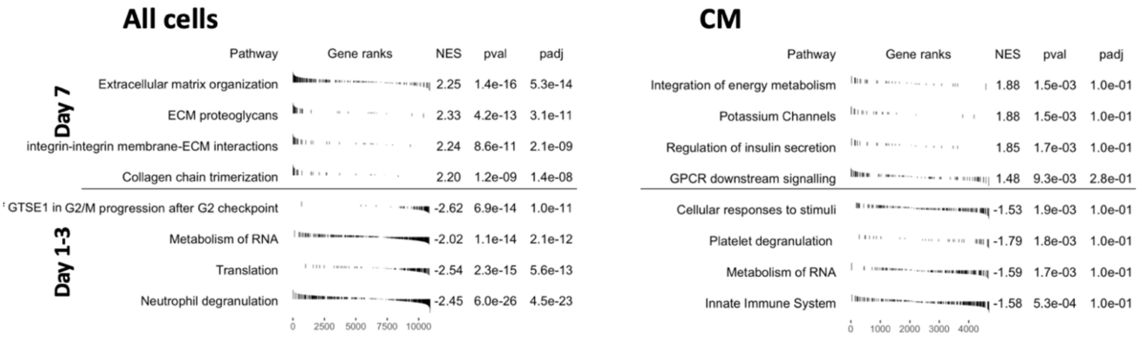

Supplementary Figure 1. GSEA of pathways enriched on day 1-3 versus day 7 post-lesion, comparing all cells (left) or CM only (right). Adjusted *p*-values were calculated by Benjamini-Hochberg method.

Supplementary Figure 2

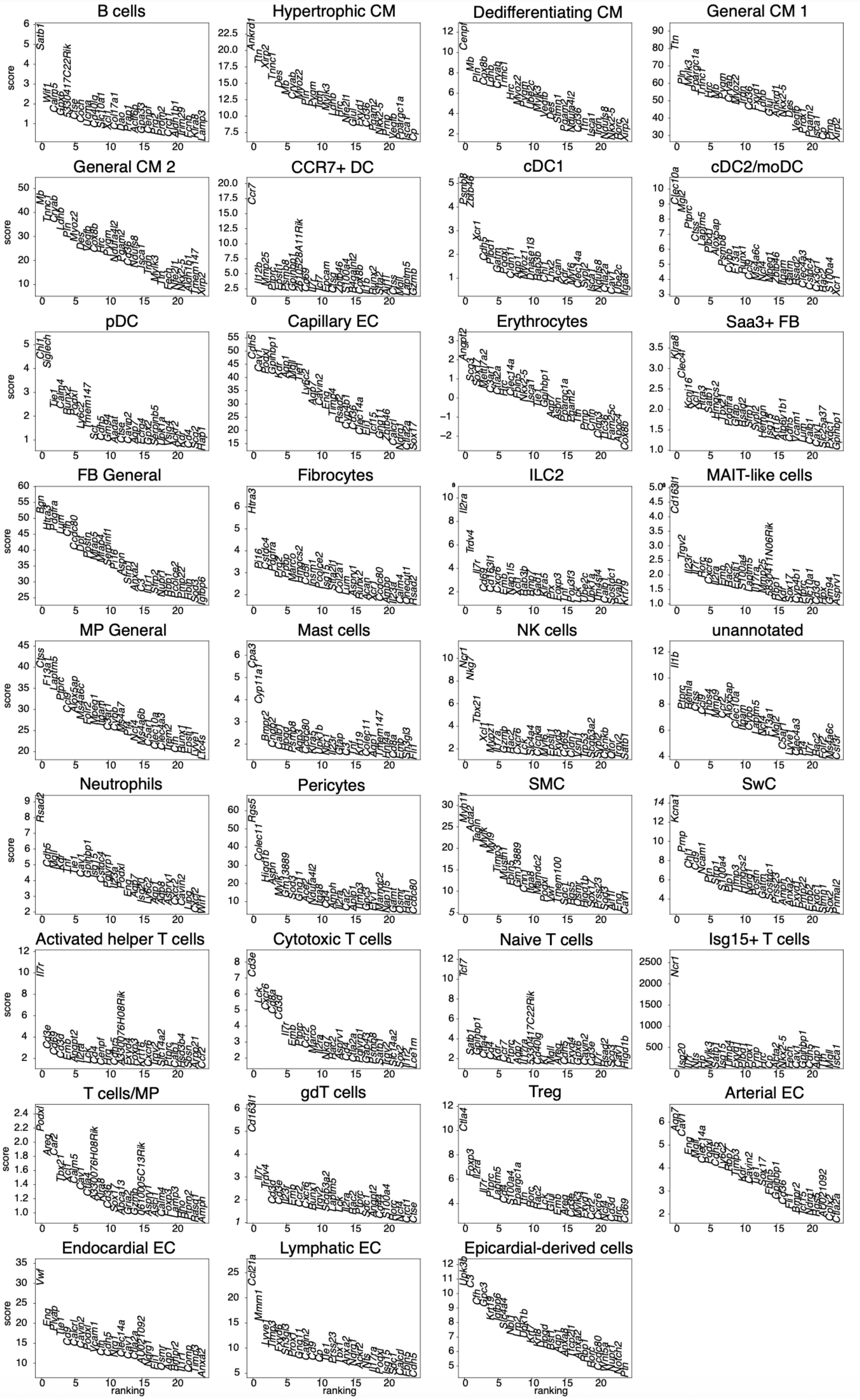

Supplementary Figure 2. **Gene expression of spatial-annotated cell types**. Top 25 differentially expressed genes (DEGs) of NiCo-annotated cell types for the Cryo day 7 spatial data.

Supplementary Figure 3

a

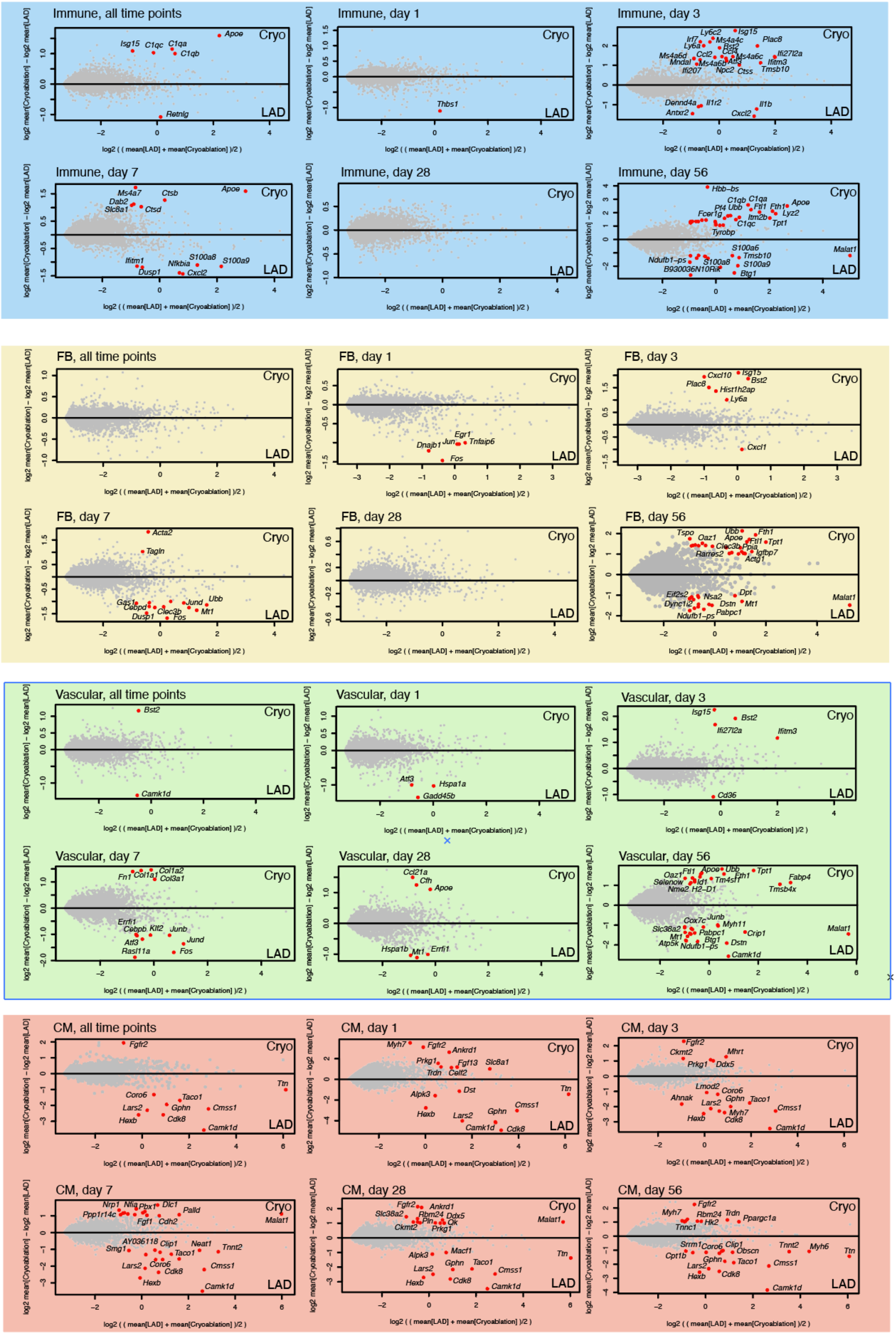

b

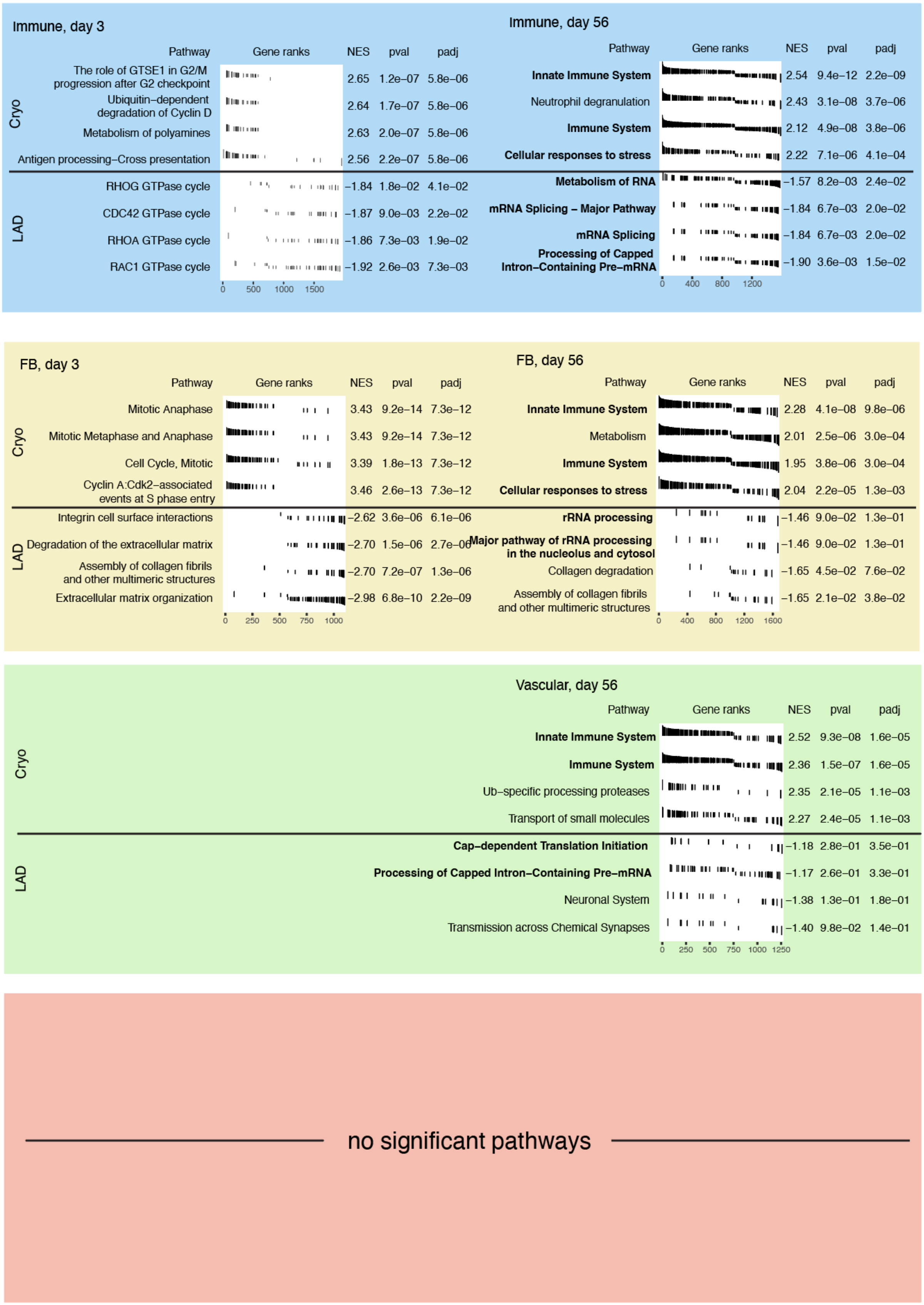

Immune, day 3
Pathway
Gene ranks
NES
pval
padj
Cryo
The role of GTSE1 in G2/M progression after G2 checkpoint 2.65 1.2e−07 5.8e−06
Ubiquitin−dependent degradation of Cyclin D 2.64 1.7e−07 5.8e−06
Metabolism of polyamines 2.63 2.0e−07 5.8e−06
Antigen processing−Cross presentation 2.56 2.2e−07 5.8e−06
LAD
RHOG GTPase cycle −1.84 1.8e−02 4.1e−02
CDC42 GTPase cycle −1.87 9.0e−03 2.2e−02
RHOA GTPase cycle −1.86 7.3e−03 1.9e−02
RAC1 GTPase cycle −1.92 2.6e−03 7.3e−03
0 500 1000 1500
Immune, day 56
Innate Immune System 2.54 9.4e−12 2.2e−09
Neutrophil degranulation 2.43 3.1e−08 3.7e−06
Immune System 2.12 4.9e−08 3.8e−06
Cellular responses to stress 2.22 7.1e−06 4.1e−04
Metabolism of RNA −1.57 8.2e−03 2.4e−02
mRNA Splicing – Major Pathway −1.84 6.7e−03 2.0e−02
mRNA Splicing −1.84 6.7e−03 2.0e−02
Processing of Capped Intron−Containing Pre−mRNA −1.90 3.6e−03 1.5e−02
0 400 800 1200
FB, day 3
Mitotic Anaphase 3.43 9.2e−14 7.3e−12
Mitotic Metaphase and Anaphase 3.43 9.2e−14 7.3e−12
Cell Cycle, Mitotic 3.39 1.8e−13 7.3e−12
Cyclin A:Cdk2−associated events at S phase entry 3.46 2.6e−13 7.3e−12
Integrin cell surface interactions −2.62 3.6e−06 6.1e−06
Degradation of the extracellular matrix −2.70 1.5e−06 2.7e−06
Assembly of collagen fibrils and other multimeric structures −2.70 7.2e−07 1.3e−06
Extracellular matrix organization −2.98 6.8e−10 2.2e−09
0 250 500 750 1000
FB, day 56
Innate Immune System 2.28 4.1e−08 9.8e−06
Metabolism 2.01 2.5e−06 3.0e−04
Immune System 1.95 3.8e−06 3.0e−04
Cellular responses to stress 2.04 2.2e−05 1.3e−03
rRNA processing −1.46 9.0e−02 1.3e−01
Major pathway of rRNA processing in the nucleolus and cytosol −1.46 9.0e−02 1.3e−01
Collagen degradation −1.65 4.5e−02 7.6e−02
Assembly of collagen fibrils and other multimeric structures −1.65 2.1e−02 3.8e−02
0 400 800 1200 1600
Vascular, day 56
Innate Immune System 2.52 9.3e−08 1.6e−05
Immune System 2.36 1.5e−07 1.6e−05
Ub−specific processing proteases 2.35 2.1e−05 1.1e−03
Transport of small molecules 2.27 2.4e−05 1.1e−03
Cap−dependent Translation Initiation −1.18 2.8e−01 3.5e−01
Processing of Capped Intron−Containing Pre−mRNA −1.17 2.6e−01 3.3e−01
Neuronal System −1.38 1.3e−01 1.8e−01
Transmission across Chemical Synapses −1.40 9.8e−02 1.4e−01
0 250 500 750 1000 1250
no significant pathways

c

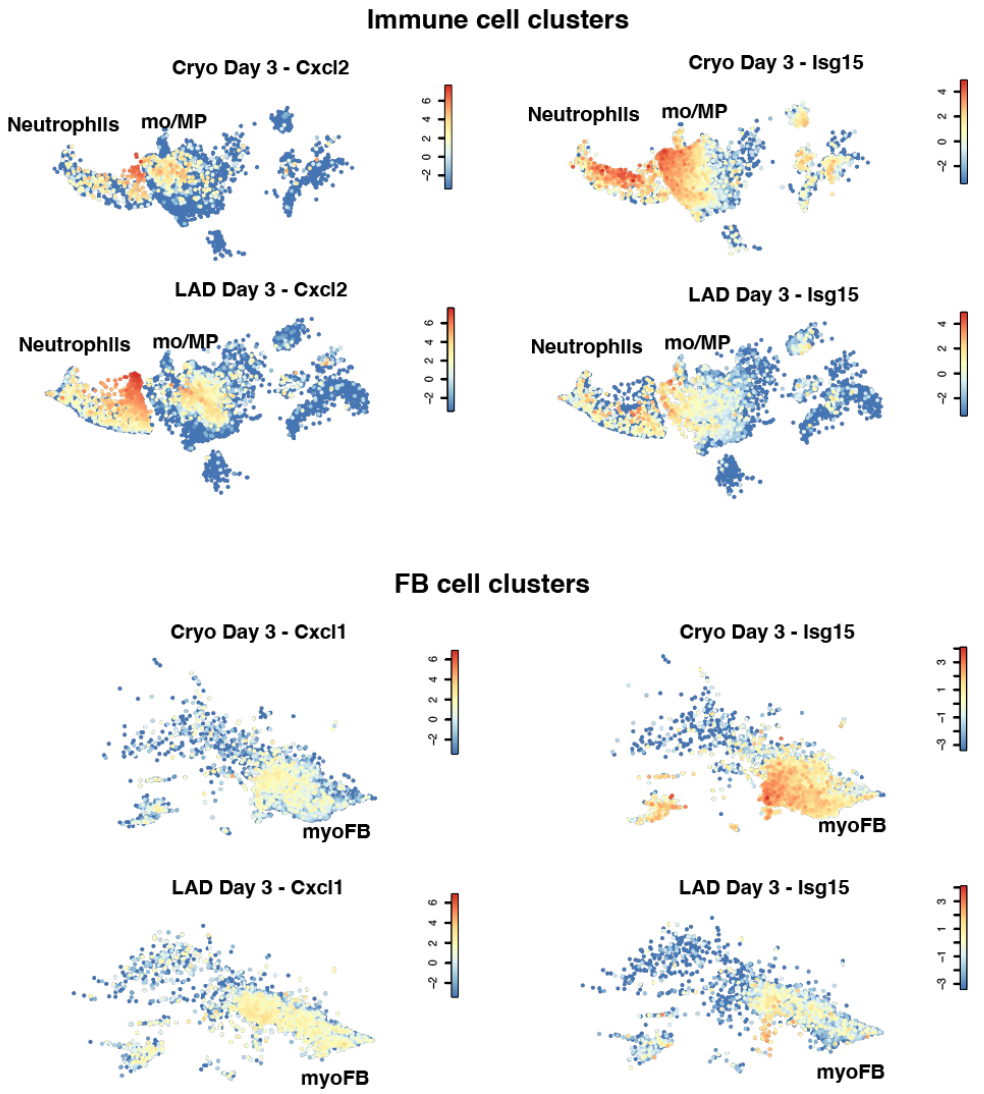

Supplementary Figure 3. **a**, differentially-expressed gene (DEG) analysis, and **b,** gene set enrichment analysis (GSEA) of major cell type components. In (**a**), genes labelled in red have log2FC > 1 and adjusted $p < 0.05$, calculated by negative binomial test with FDR correction In (**b**), Adjusted p-values were calculated by Benjamini-Hochberg method. **c**, representative gene expression UMAPs of immune and FB subclusters for selected genes identified in the DEG analysis, showing their individual expression in Cryo and LAD, respectively.

Supplementary Figure 4

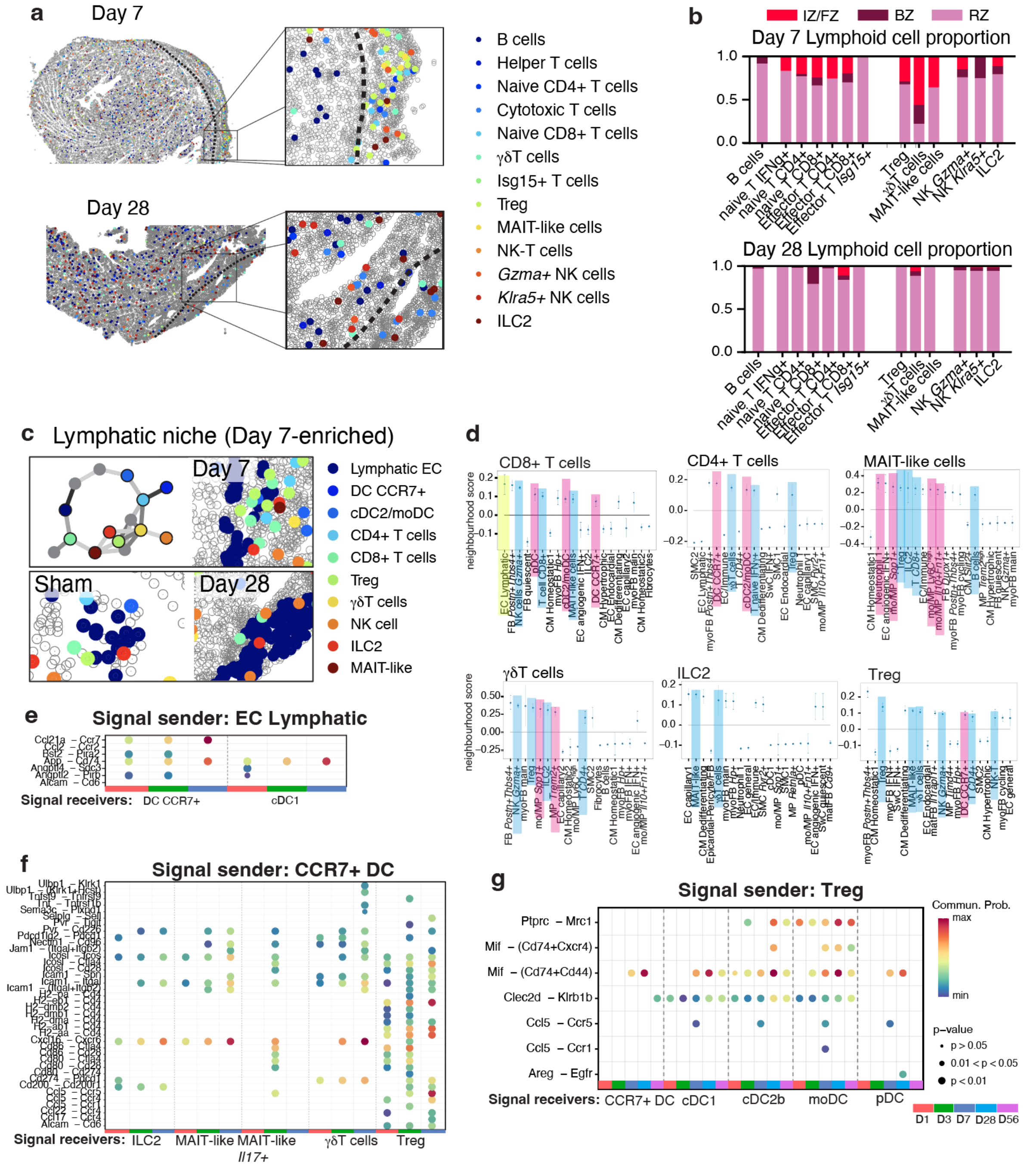

Supplementary Figure 4. **a**, day 7 and day 28 spatial maps highlighting the localization of lymphocytes. **b**, stacked barplots showing spatial distributions of lymphocyte populations. **c**, inferred lymphocyte niches from NiCo neighborhood analysis. The network (upper left panel) indicates lymphocyte proximities on day 7. **d**, NiCo neighborhood co-localization scores of lymphocytes with different cell types. Myeloid cells and lymphocytes, and lymphatic EC are highlighted in red, blue, and yellow, respectively. **e**, CellChat ligand-receptor interaction analysis of scRNA-seq data, showing interaction of DC with lymphatic EC on day 3 (red), 7 (green) and 28 (blue). **f**, CellChat analysis of the same time points, between CCR7+ DC and lymphocytes. **g**,

CellChat analysis of Treg – DC interactions, from day 1 – 56 post-injury. For (**e-g**), p-values were calculated by permutation test.

Supplementary Figure 5

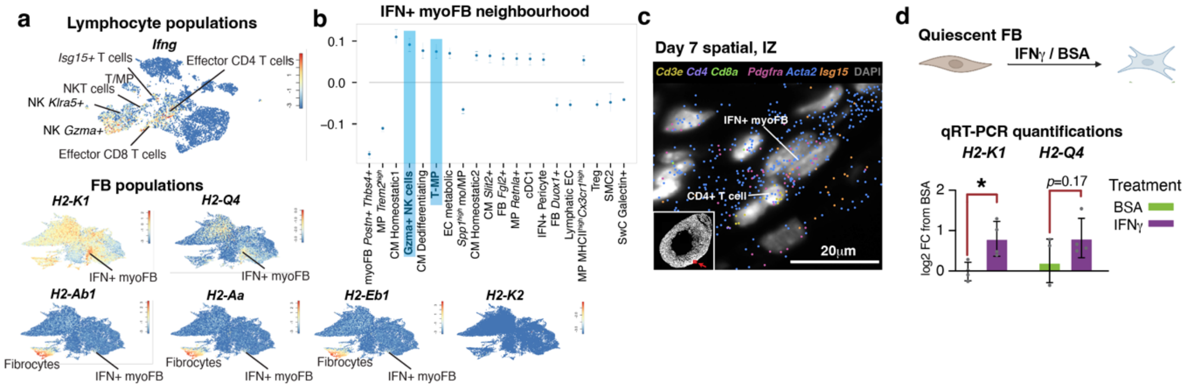

Supplementary Figure 5. **a**, gene expression UMAPs of lymphocyte and FB populations, showing IFN signaling in CD4+ T cells, and antigen presentation genes in IFN+ myoFB. **b**, spatial neighborhood analysis of IFN+ myoFB. Highlighted in blue are lymphocyte cell types, suggesting high proximity between myoFB and these lymphocytes. Neighborhood scores, also known as the niche coefficients, are the regression weights learned when modeling how the composition or state of neighboring cell types predicts a central cell type's identity or latent state. **c**, Xenium day 7 section, showing IFN+ myoFB (*Pdgfra+Acta2+Isg15+*) and CD4+ T cells (*Cd3e+Cd4+Cd8a-*) in close proximity in the IZ. Three independent experiments were performed. **d**, schematic diagram of FB activation experiment by IFNγ, and qPCR quantification of antigen-presenting gene expressions. Statistical significance was calculated by unpaired two-tailed t-test for two experimental groups. * $p < 0.05$, ** $p < 0.01$, *** $p < 0.001$, and **** $p < 0.0001$; ns, not significant. Error bars, standard deviation centered at the mean of datapoints. n = 4 biological samples per group, each data point represents one sample.

Supplementary Figure 6

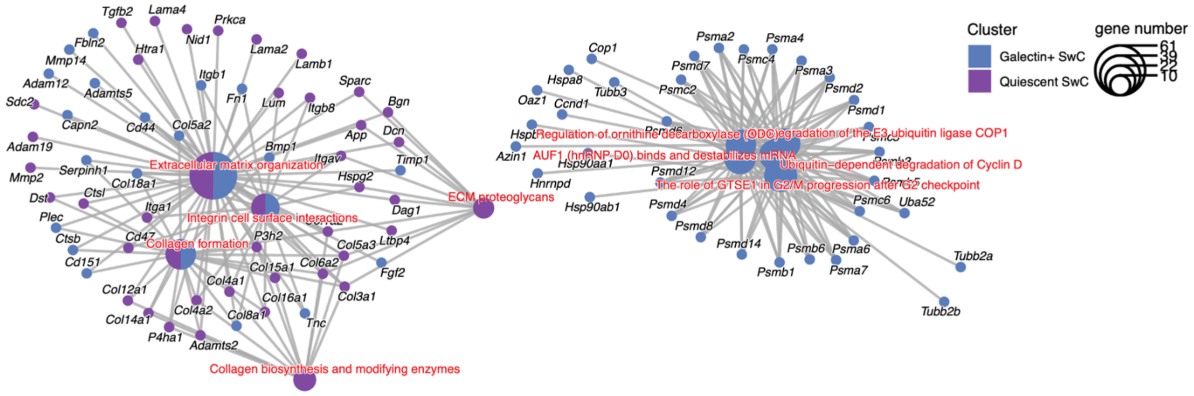

Supplementary Figure 6. Gene concept network plot showing differentially enriched pathways and the contributing genes, comparing between Galectin+ and quiescent SwC in the single cell data.

Supplementary Figure 7

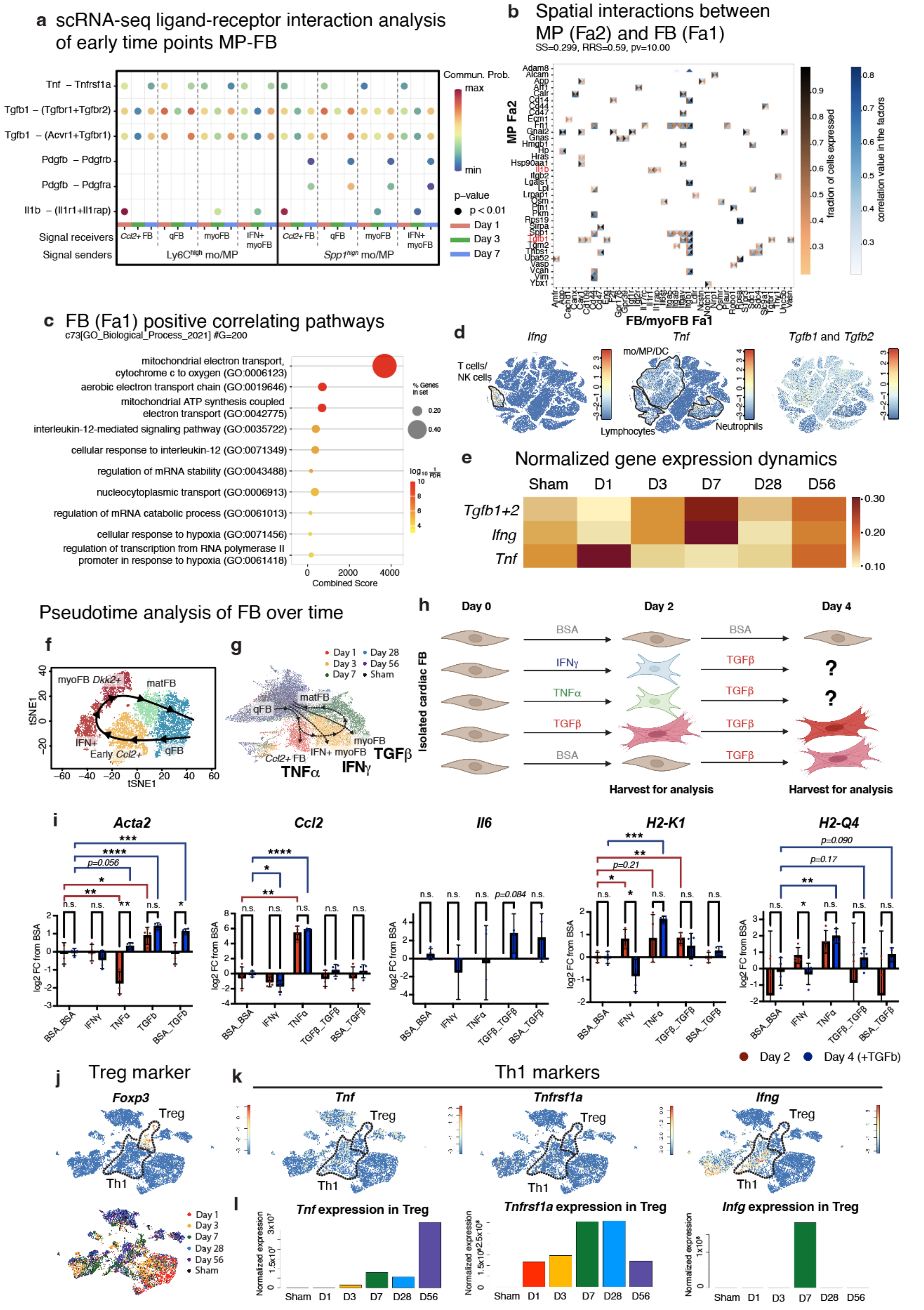

Supplementary Figure 7. a, CellChat ligand-receptor interaction analysis of the day 1, 3 and 7 post-LAD/Cryo scRNA-seq data, focusing on interactions between mo/MP and FB. p-values were calculated by permutation test. **b**, spatial cell-cell interaction analysis

between MP and FB on day 7 Cryo. **c**, enriched pathways deduced from the genes that are positively correlated to "gene-factor 1(Fa1)" of day 7 FB. Adjusted p-values were calculated by Benjamini-Hochberg method. **d**, gene expression UMAPs of all cell types, showing cell types expressing IFNγ, TNFα and TGFβ genes. **e**, heatmap of normalized gene expression of the genes in **d**, showing expression changes throughout different time points post-injury. **f and g**, pseudotime analysis and schematic diagram highlighting potential cell fate changes of FB post-injury. **h**, schematic diagram of experimental strategy to determine how FB cell fates are affected by IFNγ, TNFα and TGFβ, respectively. **i**, qRT-PCR quantifications of FB cell state markers on day 2 and 4 of the culturing experiment. Statistical significance was calculated by unpaired two-tailed t-test for two experimental groups. * $p < 0.05$, ** $p < 0.01$, *** $p < 0.001$; ns, not significant. Error bars, standard deviation centered at the mean of datapoints. n = 4 biological replicates. **j**, UMAPs of lymphocyte populations, indicating gene expression and timepoint and highlighting Treg and Th1 cell types. **k**, gene expression UMAPs of Th1 markers. **l**, barplot showing expression of Th1 markers over time post-injury.

Supplementary Figure 8

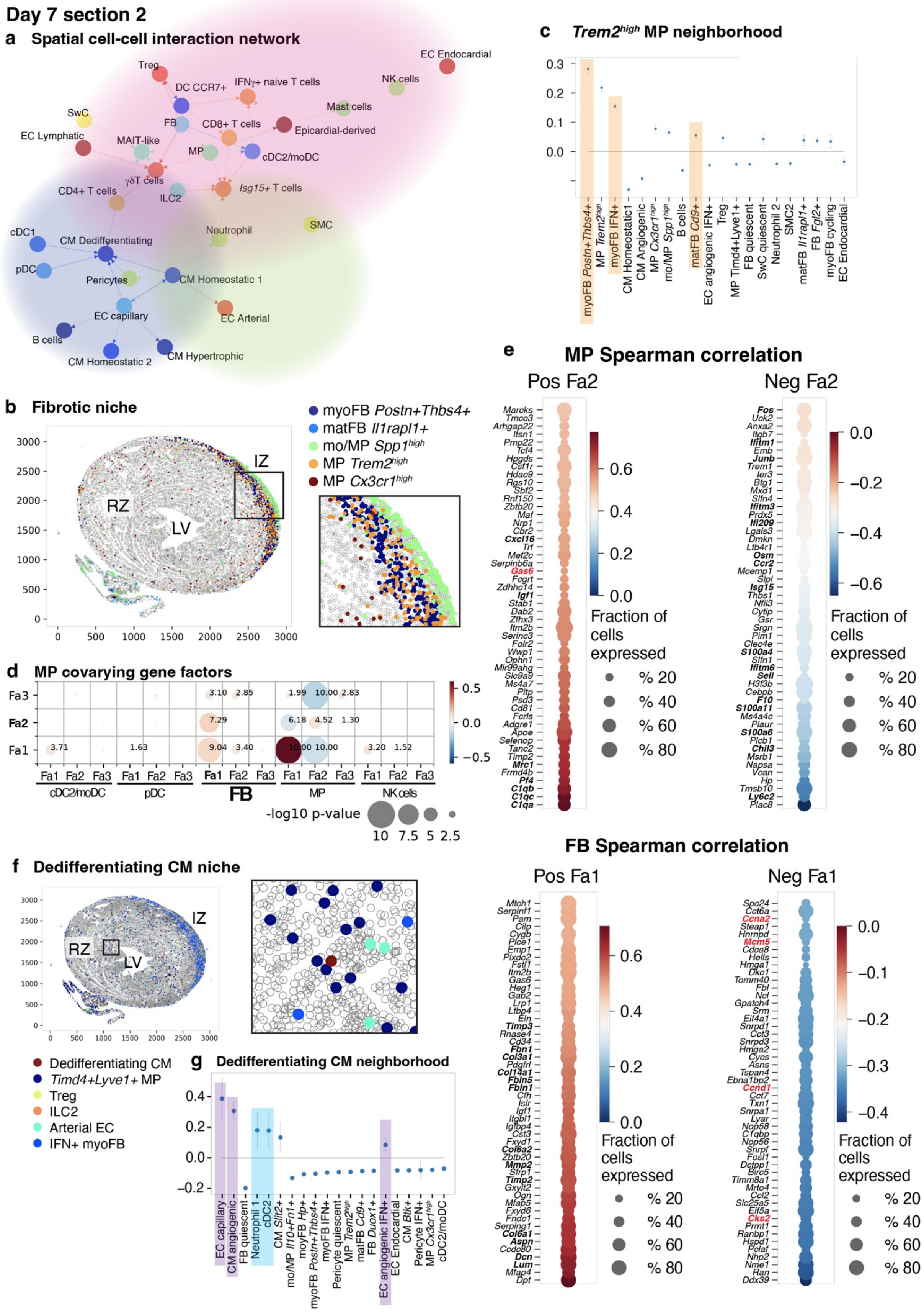

Supplementary Figure 8. NiCo analyses of day 7 heart section that is not presented in the main and extended data figures. **a,** NiCo spatial interaction network prediction.

Thickness of edges indicates interaction strength. Arrowheads point from niche cell to central cell. Red shading, IZ/FZ-enriched; green shading, BZ-enriched; blue shading; RZ-enriched. **b**, Spatial maps showing MP and FB subtypes in the wound, indicating a fibrotic FB-MP niche. **c**, day 7 neighborhoods (NiCo interaction scores) surrounding *Trem2*^{high} MP, with FB subtypes highlighted in yellow. Neighborhood scores, also known as the niche coefficients, are the regression weights learned when modeling how the composition or state of neighboring cell types predicts a central cell type's identity or latent state. **d**, spatial latent factor (Fa) co-variations of MP neighborhood in day 7 heart. Circle size scales linearly with $-\log_{10}$(p-value), and circle color indicates ridge regression coefficients. The multivariate regression p-value was derived from two-tailed t-statistics. **e**, genes positively and negatively correlated with MP Fa3 (left) and FB Fa2 (right). Highlighted genes for MP Fa3, secreted ligands (positive) and early proinflammatory/IFN pathway (negative). Highlighted genes for FB Fa2, ECM genes (positive) and cell cycle (negative). **f**, dedifferentiating CM niche visualization in days 7 myocardium, visualized by the spatial transcriptomic data. **g**, spatial neighborhoods (NiCo interaction scores) of dedifferentiating CM on Day 7.

Supplementary Figure 9

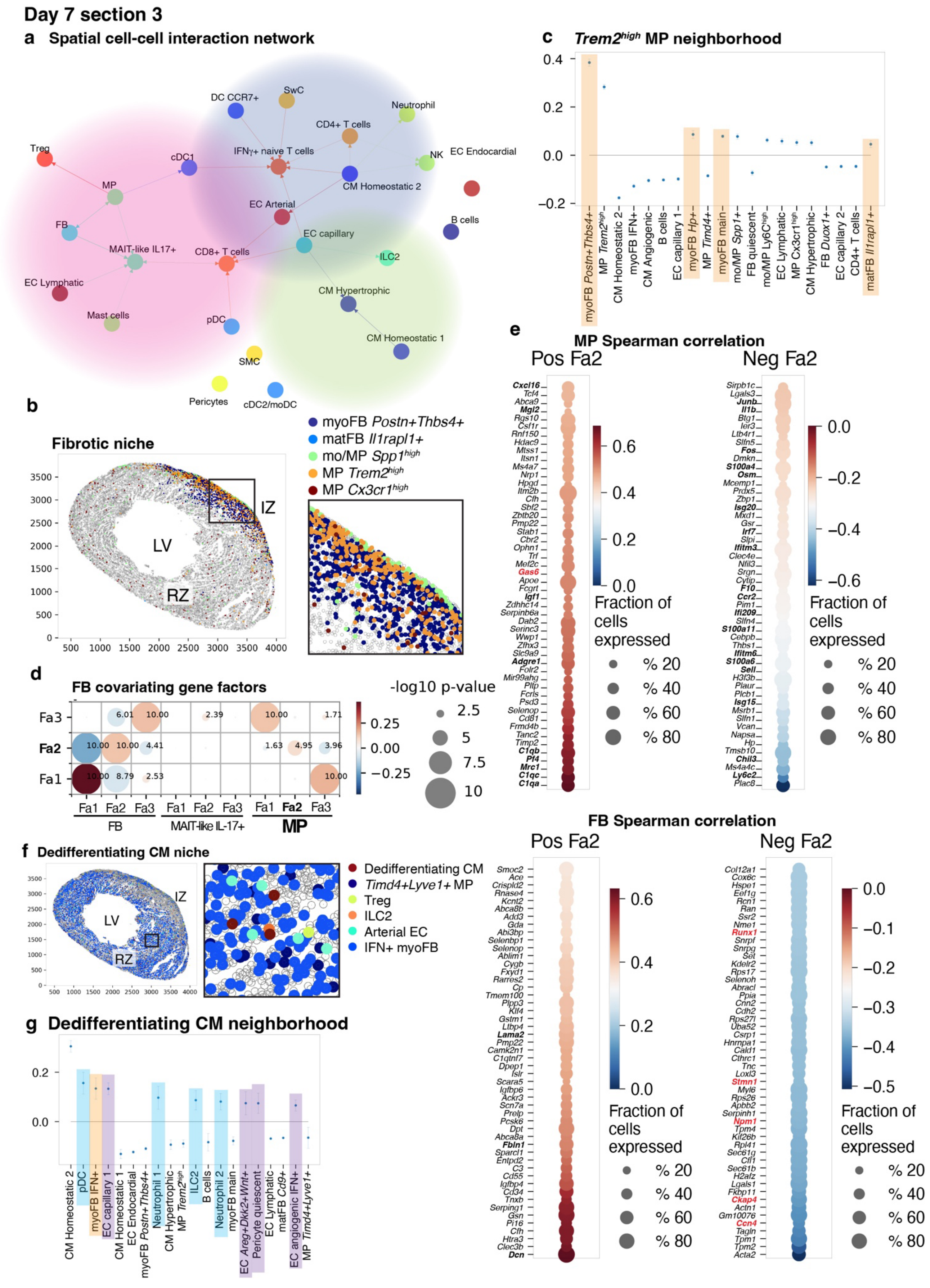

Supplementary Figure 9. NiCo analyses of day 7 heart section that is not presented in the main and extended data figures. **a,** NiCo spatial interaction network prediction. Thickness of edges indicates interaction strength. Arrowheads point from niche cell to central cell. Red shading, IZ/FZ-enriched; green shading, BZ-enriched; blue shading; RZ-enriched. **b**, Spatial maps showing MP and FB subtypes in the wound, indicating a fibrotic FB-MP niche. **c**, day 7 neighborhoods (NiCo interaction scores) surrounding *Trem2*^{high} MP, with FB subtypes highlighted in yellow. Neighborhood scores, also known as the niche coefficients, are the regression weights learned when modeling how the composition or state of neighboring cell types predicts a central cell type's identity or latent state. **d**, spatial latent factor (Fa) co-variations of MP neighborhood in day 7 heart. Circle size scales linearly with $-\log_{10}$(p-value), and circle color indicates ridge regression coefficients. The multivariate regression p-value was derived from two-tailed t-statistics. **e**, genes positively and negatively correlated with MP Fa3 (left) and FB Fa2 (right). Highlighted genes for MP Fa3, secreted ligands (positive) and early proinflammatory/IFN pathway (negative). Highlighted genes for FB Fa2, ECM genes (positive) and cell cycle (negative). **f**, dedifferentiating CM niche visualization in days 7 myocardium, visualized by the spatial transcriptomic data. **g**, spatial neighborhoods (NiCo interaction scores) of dedifferentiating CM on Day 7.

Supplementary Figure 10

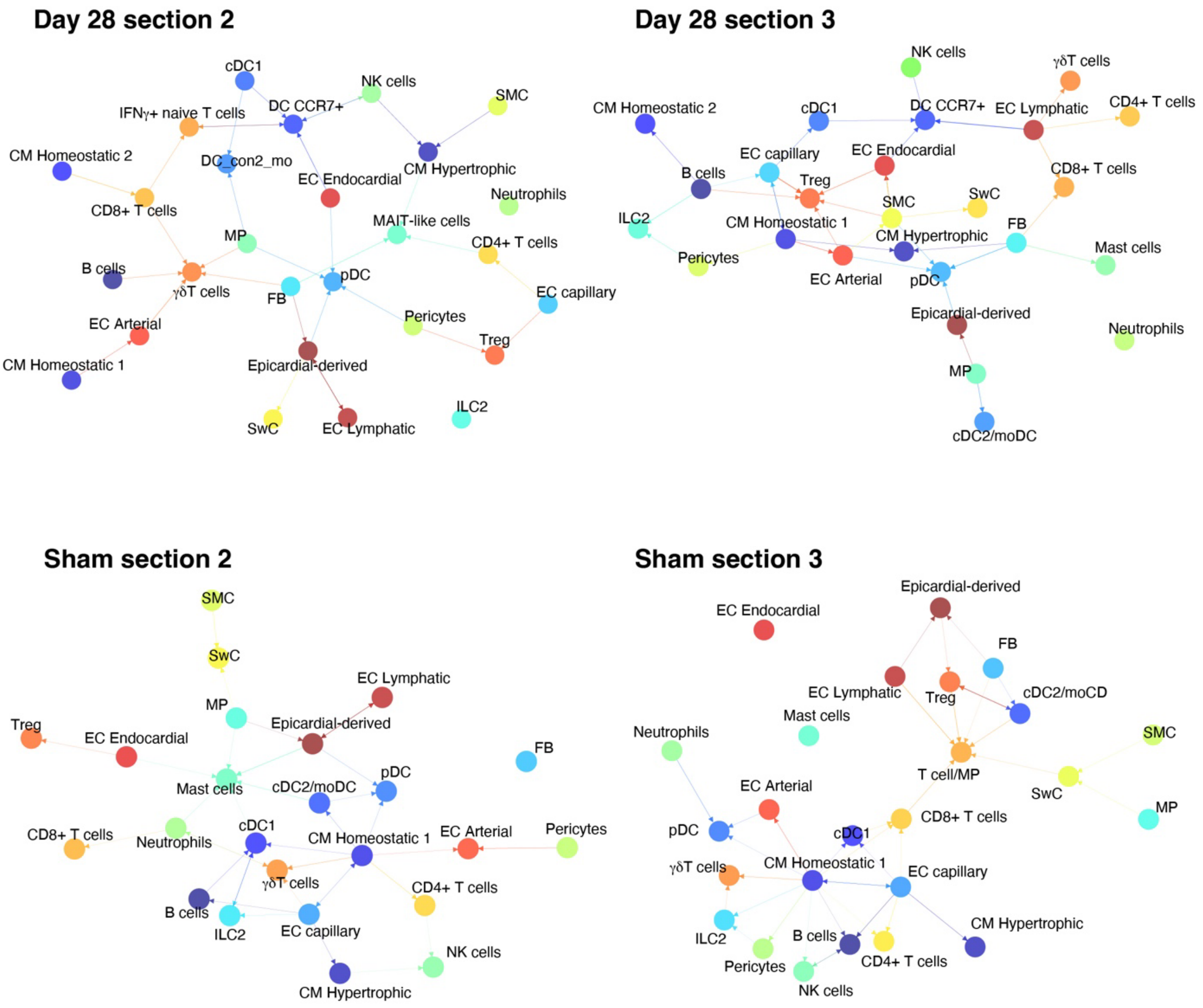

Supplementary Figure 10. NiCo spatial interaction network predictions of day 28 and sham heart sections, that are not presented in the main figures. Thickness of edges indicates interaction strength. Arrowheads point from niche cell to central cell. Red shading, IZ/FZ-enriched; green shading, BZ-enriched; blue shading, RZ-enriched.

Supplementary Figure 11

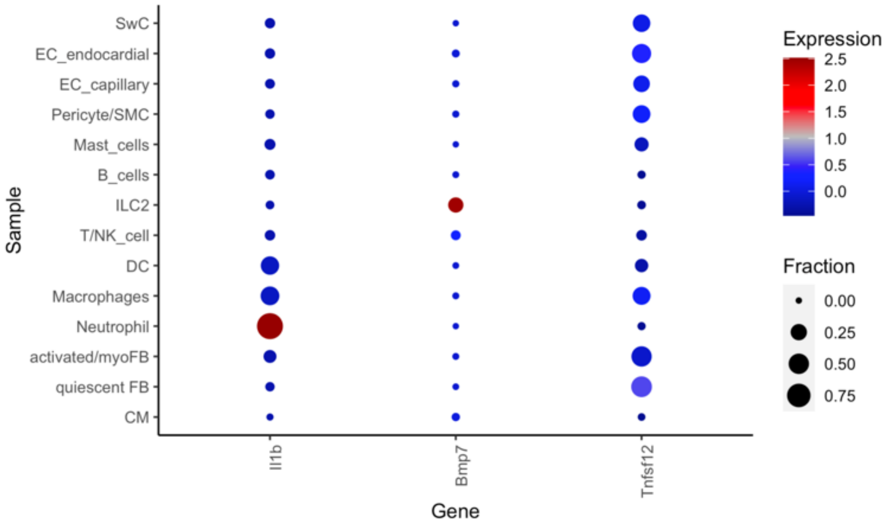

Supplementary Figure 11. Dotplot showing expression of proposed CM dedifferentiation niche ligands' expression among different cell types in the scRNA-seq data.

Supplementary Figure 12

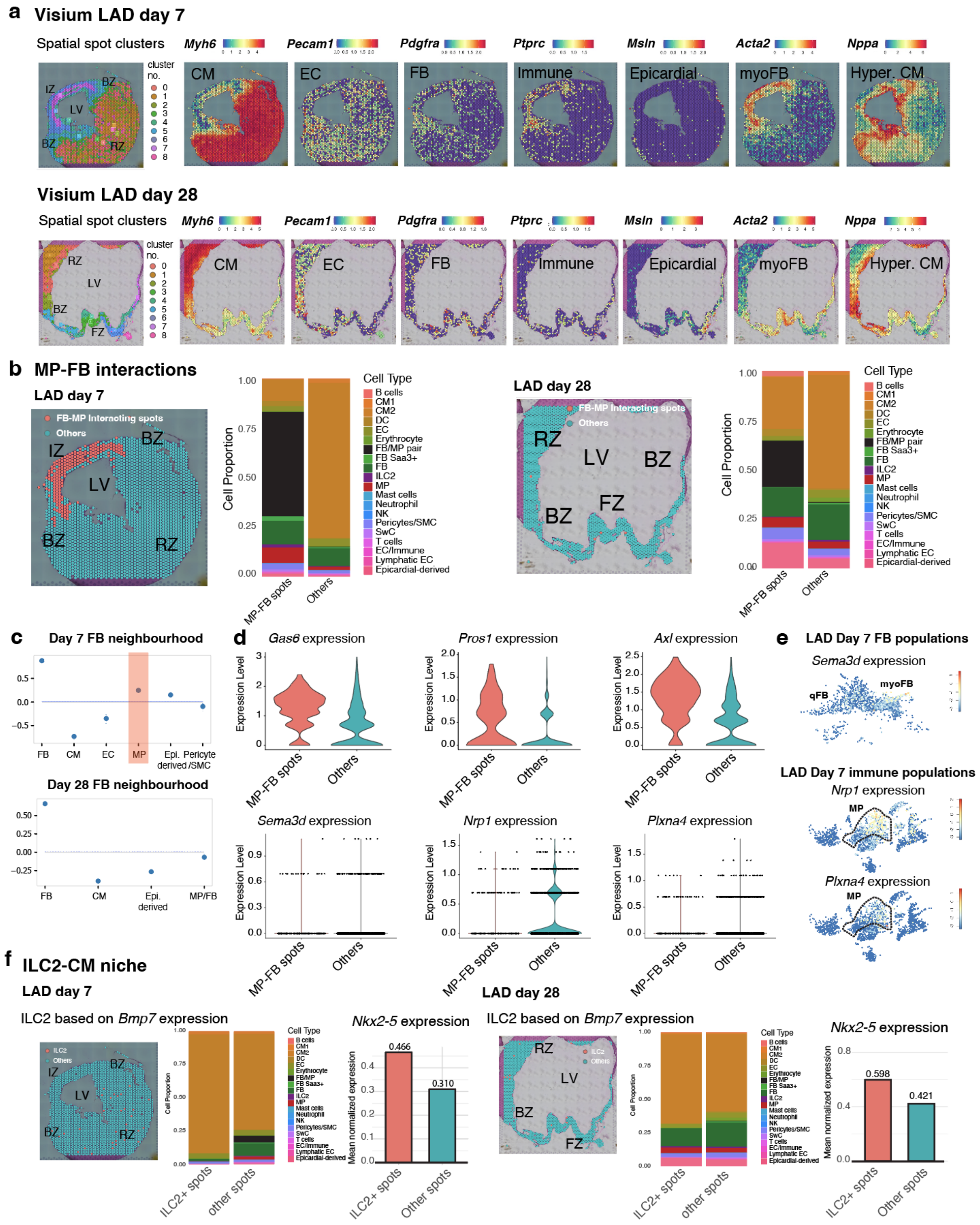

Supplementary Figure 12. Analysis of King group[1] Visium data. **a**, overview of LAD day 7 and day 8 Visium slides, showing tissue zones and major cell type markers. **b**, maps highlighting MP-FB interacting spots on both slides, and showing the cell type composition in these spots versus others. **c**, NiCo neighborhood analysis of the FB niche in LAD day 7 and 28. Neighborhood scores, also known as the niche coefficients, are the regression weights learned when modeling how the composition or state of neighboring cell types predicts a central cell type's identity or latent state. **d**, gene expression of members of the Gas6/Pros1-Axl interaction and Sema3D-Nrp1/Plxna4 interaction

between FB and MP, identified in day 7 Cryo spatial samples. **e**, Sema3D-Nrp1/Plxna4 gene expression in our LAD scRNA-seq data. **f**, ILC2-CM interacting niche. Presence of ILC2 in spots was identified by the expression of ILC2+ marker *Bmp7*. To identify the presence of ILC2-dedifferentiating CM niche, CM progenitor marker *Nkx2-5* expression was compared between ILC2+ versus ILC2- spots.

Supplementary Figure 13

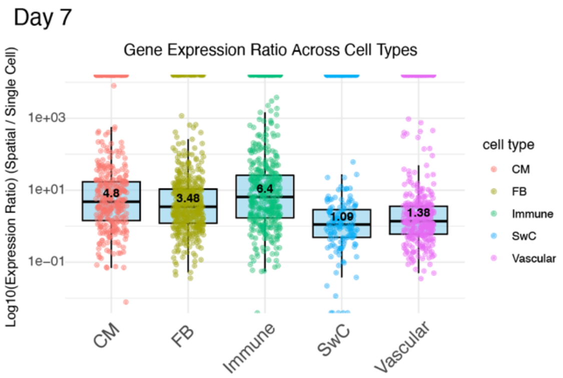

Supplementary Figure 13. Gene expression ratio between spatial / single-cell and single-nucleus RNA-seq data, across major cell types on day 7 and 28, respectively. Box center, median. Box upper and lower bounds, 25 and 75 percentiles. Whisker maxima, 75 percentile value + 1.5 x interquartile range. Whisker minima, 25 percentile value - 1.5 x interquartile range.

Supplementary Figure 14

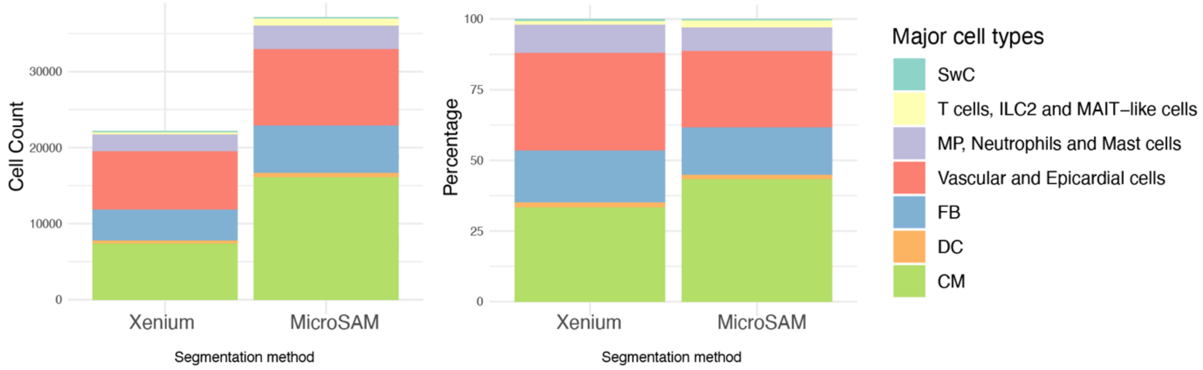

Supplementary Figure 14. Absolute (left) and percentage (right) cell count of different cell types in the day 7 Cryo section, comparing between the standard Xenium and Micro-SAM spatial cell segmentation methods.
