# Supplementary material for: Spatiotemporal dynamics of the cardioimmune niche during lesion repair
Source: Nat Cardiovasc Res. 2025 Nov 3;4(11):1550–72. doi: 10.1038/s44161-025-00739-6 (PMC12611762; doi:10.1038/s44161-025-00739-6)
Supplement: Supplementary file 2 — Reporting Summary [file 44161_2025_739_MOESM2_ESM.pdf]

nature portfolio

Corresponding author(s): Dominic Grün

Last updated by author(s): Aug 20, 2025

Reporting Summary

Nature Portfolio wishes to improve the reproducibility of the work that we publish. This form provides structure for consistency and transparency in reporting. For further information on Nature Portfolio policies, see our Editorial Policies and the Editorial Policy Checklist.

Statistics

For all statistical analyses, confirm that the following items are present in the figure legend, table legend, main text, or Methods section.

n/a	Confirmed	
☐	☒	The exact sample size (*n*) for each experimental group/condition, given as a discrete number and unit of measurement
☐	☒	A statement on whether measurements were taken from distinct samples or whether the same sample was measured repeatedly
☐	☒	The statistical test(s) used AND whether they are one- or two-sided<br>*Only common tests should be described solely by name; describe more complex techniques in the Methods section.*
☒	☐	A description of all covariates tested
☐	☒	A description of any assumptions or corrections, such as tests of normality and adjustment for multiple comparisons
☐	☒	A full description of the statistical parameters including central tendency (e.g. means) or other basic estimates (e.g. regression coefficient) AND variation (e.g. standard deviation) or associated estimates of uncertainty (e.g. confidence intervals)
☐	☒	For null hypothesis testing, the test statistic (e.g. *F*, *t*, *r*) with confidence intervals, effect sizes, degrees of freedom and *P* value noted<br>*Give P values as exact values whenever suitable.*
☒	☐	For Bayesian analysis, information on the choice of priors and Markov chain Monte Carlo settings
☒	☐	For hierarchical and complex designs, identification of the appropriate level for tests and full reporting of outcomes
☒	☐	Estimates of effect sizes (e.g. Cohen's *d*, Pearson's *r*), indicating how they were calculated

Our web collection on statistics for biologists contains articles on many of the points above.

Software and code

Policy information about availability of computer code

Data collection	The raw and processed data are provided under the link in the manuscript.
Data analysis	Data analysis was performed with packages such as VarID and NiCo as described in the methods section

For manuscripts utilizing custom algorithms or software that are central to the research but not yet described in published literature, software must be made available to editors and reviewers. We strongly encourage code deposition in a community repository (e.g. GitHub). See the Nature Portfolio guidelines for submitting code & software for further information.

Data

Policy information about availability of data

All manuscripts must include a data availability statement. This statement should provide the following information, where applicable:

- Accession codes, unique identifiers, or web links for publicly available datasets
- A description of any restrictions on data availability
- For clinical datasets or third party data, please ensure that the statement adheres to our policy

The raw and processed single cell and spatial transcriptomic data are deposited to GEO, where the accession code and web links are indicated in the data availability statement. Accession numbers: GSE280373 and GSE280376.

Research involving human participants, their data, or biological material

Policy information about studies with human participants or human data. See also policy information about sex, gender (identity/presentation), and sexual orientation and race, ethnicity and racism.

Reporting on sex and gender	Male
Reporting on race, ethnicity, or other socially relevant groupings	European
Population characteristics	Not considered in the study design.
Recruitment	Patients with DCM
Ethics oversight	Ethical votes have been provided.

Note that full information on the approval of the study protocol must also be provided in the manuscript.

Field-specific reporting

Please select the one below that is the best fit for your research. If you are not sure, read the appropriate sections before making your selection.

☒ Life sciences ☐ Behavioural & social sciences ☐ Ecological, evolutionary & environmental sciences

For a reference copy of the document with all sections, see nature.com/documents/nr-reporting-summary-flat.pdf

Life sciences study design

All studies must disclose on these points even when the disclosure is negative.

Sample size	Number of biological replicates provided in the respective section of the manuscript.
Data exclusions	No data was excluded.
Replication	Statistical parameters were provided in the figures.
Randomization	Participants were not included in the study.
Blinding	There was no data allocated based on the group.

Reporting for specific materials, systems and methods

We require information from authors about some types of materials, experimental systems and methods used in many studies. Here, indicate whether each material, system or method listed is relevant to your study. If you are not sure if a list item applies to your research, read the appropriate section before selecting a response.

Materials & experimental systems

n/a	Involved in the study	
☐	☒	Antibodies
☒	☐	Eukaryotic cell lines
☒	☐	Palaeontology and archaeology
☐	☒	Animals and other organisms
☒	☐	Clinical data
☒	☐	Dual use research of concern
☒	☐	Plants

Methods

n/a	Involved in the study	
☒	☐	ChIP-seq
☐	☒	Flow cytometry
☒	☐	MRI-based neuroimaging

Antibodies

Antibodies used	rat Mki67-FITC antibody (Biolegend, 151211), rat CD140a-PE (ThermoFisher, 12-1401-81), mouse SMA primary antibody (Invitrogen, 14-9760-80), human Actinin(Sarcomeric)-PE (Miltenyi, 130-123-996), mouse Aurora B primary antibody (BD Bioscience, 611082), goat anti-mouse IgG-AF647 (Biolegend, 405322), mouse PDGFRa (R&D Systems, AF1062-SP), rat CD45 (Biolegend, 103108), goat GAS6 (R&D Systems, AF986-SP), rabbit PROS1 (Invitrogen, PA5-106880), goat AXL (R&D Systems, AF854-SP), mouse BMP-7

(NovusBio, NBP2-52425), rabbit GATA3 (Cell Signaling Technology, 5852T), goat KIT (R&D Systems, AF1356-SP), Rat monoclonal anti-mouse F4/80 (BD, 565614), Rat monoclonal anti-mouse CD45 (BD, 749889), Rat monoclonal anti-mouse/human Arg1 (eBioscience, 368-3697-82),Rat monoclonal anti-mouse CD206 (MMR) (BioLegend, 141717), Rat monoclonal anti-mouse CCR2 (BioLegend, 150605), Rat monoclonal anti-mouse Ly6C (BioLegend, 128033), Rat monoclonal anti-mouse/human CD11b, (BioLegend, 101257), Mouse monoclonal anti-mouse CCL5 (BioLegend, 149103), Rat monoclonal anti-mouse Trem2 (BioLegend, 824805), Rat monoclonal anti-mouse Mgl2 (CD301b), (BioLegend, 146807), Rat monoclonal anti-mouse TimD4, (BioLegend, 130009), Mouse monoclonal anti-mouse CD64, (BioLegend, 139319), Mouse monoclonal anti-human/mouse/rat Ki67 (BD, 561284), neonatal cardiomyocyte isolation antibody cocktail (Miltenyi, 130-100-825), PCM-1 antibody (MERCK, HPA023370), anti-Rabbit IgG secondary antibody (Biolegend, 406419), CD45-Pacific Blue (Biolegend, 103125), Ter119-APC/Cy7 (Biolegend, 116223), CD31-PE (Biolegend, 102507), CD146-PE (Biolegend, 134703), CD14-FITC (Biolegend, 123307) and CD11c-APC (Biolegend, 117309), CD14 (60253-1-IG, Proteintech), rabbit RPS9 (PA5-104493, Invitrogen), anti-mouse Alexa Fluor 568 (A11077, Invitrogen), anti-rabbit Alexa Fluor 488 (A11055, Invitrogen), WGA, BOT-29026-1, Biozol, CD28 (clone 37.51, Bio X Cell, BE0015-1), IFN[illegible] (clone XMG1.2, ThermoScientific, 315-05)

Validation

All antibodies are purchased where the manufacturers have specified the species, application, concentrations and in-house validations, which are available in the manufacturer webpages.

Animals and other research organisms

Policy information about studies involving animals; ARRIVE guidelines recommended for reporting animal research, and Sex and Gender in Research

Laboratory animals	C57B/6J mouse with an age from 1 - 20 weeks, Light 12h:12h (6am-6pm), Temperature: 20-24°C, Humidity: 45-46%. According to §11 Haltungsgenehmigung. sources of animals: Janvier, Charles River and CEMT (internal breeding)
Wild animals	Study did not involve wild animals.
Reporting on sex	Sex was not considered in the study design.
Field-collected samples	Study did not involve sample collected from the field.
Ethics oversight	All animal experiments were approved and conducted under the regulations of the local Government.

Note that full information on the approval of the study protocol must also be provided in the manuscript.

Plants

Seed stocks	Not applicable
Novel plant genotypes	Not applicable
Authentication	Not applicable

Flow Cytometry

Plots

Confirm that:

☒ The axis labels state the marker and fluorochrome used (e.g. CD4-FITC).

☒ The axis scales are clearly visible. Include numbers along axes only for bottom left plot of group (a 'group' is an analysis of identical markers).

☒ All plots are contour plots with outliers or pseudocolor plots.

☒ A numerical value for number of cells or percentage (with statistics) is provided.

Methodology

Sample preparation	Mouse or human cardiac and immune cells are used for the analyses. Sample preparation methods are specified in the methods section.
Instrument	Aurora Flow Cytometer (Cytek) and BD FACSCelesta Cell Analyzer (BD BioSciences, Franklin Lakes, NJ, USA)
Software	FlowJo v10 software (FlowJo LLC).

Cell population abundance	Sorting was performed for scRNA-seq and snRNA-seq experiments. Post-sort cell type proportions are determined in the sequenced and cell type clustered data.
Gating strategy	FSC/SSC gate was chosen such that cells are captured but the debris are depleted. For the gating of all color channels, the levels of "negative" gates have been defined via non-stained (negative control) samples of the corresponding channels.

☒ Tick this box to confirm that a figure exemplifying the gating strategy is provided in the Supplementary Information.
